# Chromone Derivatives and Other Constituents from Cultures of the Marine Sponge-Associated Fungus *Penicillium erubescens* KUFA0220 and Their Antibacterial Activity

**DOI:** 10.3390/md16080289

**Published:** 2018-08-20

**Authors:** Decha Kumla, José A. Pereira, Tida Dethoup, Luis Gales, Joana Freitas-Silva, Paulo M. Costa, Michael Lee, Artur M. S. Silva, Nazim Sekeroglu, Madalena M. M. Pinto, Anake Kijjoa

**Affiliations:** 1 *ICBAS*—Instituto de Ciências Biomédicas Abel Salazar, Universidade do Porto, Rua de Jorge Viterbo Ferreira, 228, 4050-313 Porto, Portugal; Decha1987@hotmail.com (D.K.); jpereira@icbas.up.pt (J.A.P.); lgales@ibmc.up.pt (L.G.); joanafreitasdasilva@gmail.com (J.F.-S.); pmcosta@icbas.up.pt (P.M.C.); 2 Interdisciplinary Centre of Marine and Environmental Research (CIIMAR), Universidade do Porto, Terminal de Cruzeiros do Porto de Lexões, Av. General Norton de Matos s/n, 4450-208 Matosinhos, Portugal; 3 Department of Plant Pathology, Faculty of Agriculture, Kasetsart University, Bangkok 10240, Thailand; tdethoup@yahoo.com; 4 Instituto de Biologia Molecular e Celular (i3S-IBMC), Universidade do Porto, Rua de Jorge Viterbo Ferreira, 228, 4050-313 Porto, Portugal; 5 Department of Chemistry, University of Leicester, University Road, Leicester LE 7 RH, UK; ml34@leicester.ac.uk; 6 Departamento de Química & QOPNA, Universidade de Aveiro, 3810-193 Aveiro, Portugal; artur.silva@ua.pt; 7 Department of Food Engineering, Faculty of Engineering and Architecture, Kilis 7 Aralık University, 79000 Kilis, Turkey; nsekeroglu@gmail.com; 8 Laboratório de Química Orgânica, Departamento de Ciências Químicas, Faculdade de Farmácia, Universidade do Porto, Rua de Jorge Viterbo Ferreira, 228, 4050-313 Porto, Portugal

**Keywords:** *Penicillium erubescens*, Aspergillaceae, marine sponge-associated fungus, *Neopetrosia* sp., chromone derivatives, GKK 1032B, pyranochromone, spirofuranochromone, antibacterial activity

## Abstract

A previously unreported chromene derivative, 1-hydroxy-12-methoxycitromycin (**1c**), and four previously undescribed chromone derivatives, including pyanochromone (**3b**), spirofuranochromone (**4**), 7-hydroxy-6-methoxy-4-oxo-3-[(1*E*)-3-oxobut-1-en-1-yl]-4*H*-chromene-5-carboxylic acid (**5**), a pyranochromone dimer (**6**) were isolated, together with thirteen known compounds: β-sitostenone, ergosterol 5,8-endoperoxide, citromycin (**1a**), 12-methoxycitromycin (**1b**), myxotrichin D (**1d**), 12-methoxycitromycetin (**1e**), anhydrofulvic acid (**2a**), myxotrichin C (**2b**), penialidin D (**2c**), penialidin F (**3a**), SPF-3059-30 (**7**), GKK1032B (**8**) and secalonic acid A (**9**), from cultures of the marine sponge- associated fungus *Penicillium erubescens* KUFA0220. Compounds **1a**–**e**, **2a**, **3a**, **4**, **7**–**9**, were tested for their antibacterial activity against Gram-positive and Gram-negative reference and multidrug-resistant strains isolated from the environment. Only **8** exhibited an in vitro growth inhibition of all Gram-positive bacteria whereas **9** showed growth inhibition of methicillin-resistant *Staphyllococus aureus* (MRSA). None of the compounds were active against Gram-negative bacteria tested.

## 1. Introduction

The fungi of the genus *Penicillium* (Family Aspergillaceae) are the most common fungi occurring in a diverse range of habitats from soil to vegetation to various food products, air, indoor environments, and marine environments. They have a worldwide distribution and a large economic impact on human life [1]. The marine-derived *Penicillium* species can be found to be associated with a variety of marine invertebrates such as marine sponges, corals, and tunicates, as well as with fish, marine algae, mangroves and also from the sediments; although sediments and sponges are their main sources or hosts for producing new marine natural products. Interestingly, marine-derived *Penicillium* species produce diverse structural classes of secondary metabolites such as polyketides, sterols, terpenoids, alkaloids, among others, and more than half of these metabolites exhibited bioactivities [2].

Thus, in our ongoing search for antibiotics from marine-derived fungi from the tropical sea, we investigated secondary metabolites from cultures of *Penicillium erubescens* KUFA 0220, which was isolated from the marine sponge *Neopetrosia* sp., collected from the coral reef at Samaesan Island, Chonburi province, in the Gulf of Thailand.

**Figure 1:**
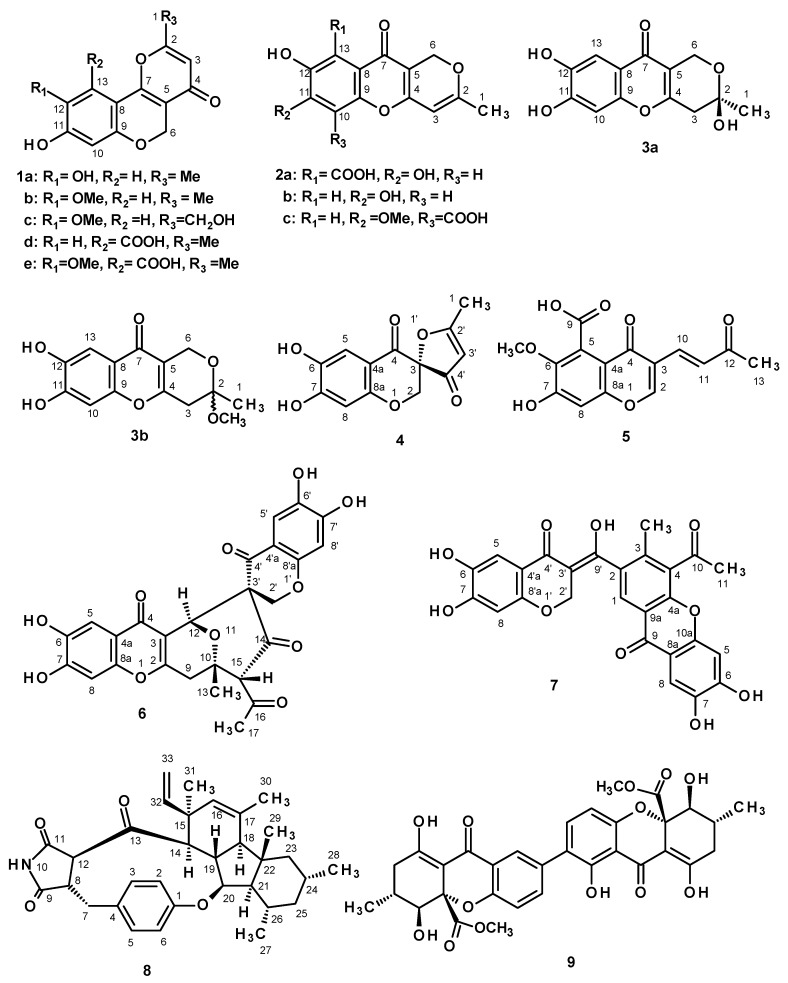
The structures of some secondary metabolites, isolated from cultures of the marine sponge-associated fungus *P. erubescens* KUFA 0220.

Chromatographic fractionation and the further purification of the crude ethyl acetate extract of the cultures of *P. erubescens* KUFA 0220, furnished an unreported chromene derivative, 1-hydroxy-12-methoxycitromycin (**1c**), and four previously undescribed chromone derivatives, including a pyanochromone (**3b**), a spirofuranochromone (**4**), 7-hydroxy-6-methoxy-4-oxo-3-[(1*E*)-3-oxobut-1-en-1-yl]-4*H*-chromene-5-carboxylic acid (**5**), and a pyranochromone dimer (**6**) (Figure 1), in addition to thirteen known compounds: β-sitostenone [3,4], citromycin (**1a**) [5], 12-methoxycitromycin (**1b**) [5], myxotrichin D (**1d**) [6], 12-methoxycitromycetin (**1e**) [5], anhydrofulvic acid (**2a**) [7,8], myxotrichin C (**2b**) [6], penialidin D (**2c**) [9], penialidin F (**3a**) [9,10], SPF-3059-30 (**7**) [11], GKK1032B (**8**) [12,13,14] and secalonic acid A (**9**) [3] (Figure 1). The structures of the previously undescribed compounds were established based on extensive analyses of their 1D and 2D NMR as well as HRMS data while the identity of the known compounds was elucidated by comparison of their ^1^H and ^13^C NMR data with those reported in the literature. The absolute configuration of the stereogenic carbon of the previously unreported **4** was established by an X-ray analysis whereas those of the previously undescribed **6** and penialidin F (**3a**) were determined by comparison of their calculated and experimental ECD spectra.

Compounds **1a**–**e**, **2a**, **3a**, **4**, **7**–**9** were tested for their antibacterial activity against five reference bacterial strains consisting of three Gram-positive (*Staphylococcus aureus* ATCC 29213, *Enterococcus faecium* ATCC 19434 and *Enterococcus faecalis* ATCC 29212) and two Gram-negative bacteria (*Escherichia coli* ATCC 25922 and *Pseudomonas aeruginosa* ATCC 27853), three multidrug-resistant isolates from the environment (MRSA *S. aureus* 66/1, VRE *E. faecium* 1/6/63 and *E. faecalis* B3/101) and a clinical isolate ESBL *E. coli* SA/2. Some of the isolated compounds were also investigated for their capacity to inhibit biofilm formation in the four reference strains as well as for their potential synergism with the clinically used antibiotics against multidrug-resistant isolates from the environment.

## 2. Results and Discussion

The structure of β-sitostenone [3], ergosterol 5,8-endoperoxide [4] (Figure S1), citromycin (**1a**) [5], 12-methoxycitromycin (**1b**) [5], myxotrichin D (**1d**) [6], 12-methoxycitromycetin (**1e**) [5], anhydrofulvic acid (**2a**) [7,8], myxotrichin C (**2b**) [6], penialidin D (**2c**) [9], penialidin F (**3a**) [9,10], SPF-3059-30 (**7**) [11], GKK1032B (**8**) [12,13,14], and secalonic acid A (**9**) [3,15] (Figure 1) were elucidated by analysis of their 1D and 2D NMR spectra as well as HRMS data, and also by comparison of their spectral data (Figures S2–S11, S15–S29, S45–S50, S52 and S53) to those reported in the literature. In the case of GKK1032B (**8**), the X-ray analysis was also performed to confirm the absolute configurations of all the stereogenic centers (Figure S51).

Compound **1c** was isolated as a white solid (mp 232–233 °C), and its molecular formula C_14_H_12_O_6_ was established based on its (+)-HRESIMS *m*/*z* 277.0715 [M + H]^+^, (calculated 277.0712 for C_14_H_13_O_6_), indicating nine degrees of unsaturation. The IR spectrum showed absorption bands for the hydroxyl (3420 cm^−1^), conjugated ketone carbonyl (1662 cm^−1^), aromatic (1627, 1555 cm^−1^), and ether (1270 cm^−1^) groups. The ^13^C NMR spectrum of **1c** (Table 1, Figure S11) displayed fourteen carbon signals which, according to DEPTs and HSQC spectra (Table 1, Figure S12), can be classified as one conjugated ketone carbonyl (δ_C_ 174.8), seven quaternary sp^2^ (δ_C_ 167.3, 155.2, 152.2, 151.9, 143.6, 111.2, 105.9), three methine sp^2^ (δ_C_ 110.7, 106.5, 104.1), two oxymethylene sp^3^ (δ_C_ 62.5 and 59.5), and one methoxyl (δ_C_ 56.4) carbon. The ^1^H NMR spectrum (Table 1, Figure S10) showed two aromatic singlets at δ_H_ 7.15 and 6.44, another singlet of one olefinic proton at δ_H_ 6.25, two singlets of oxymethylene protons at δ_H_ 5.02 (2H) and 4.41 (2H), and a singlet of methoxyl protons at δ_H_ 3.80 (3H). The general features of the ^1^H and ^13^C NMR spectra of **1c** resembled those of 12-methoxycitromycin (**1b**), which was previously isolated from the Australian marine-derived and terrestrial *Penicillium* spp. [5], and also isolated in this work. The only difference between the two compounds is the methyl group in **1b** (δ_H_ 2.34, d, *J* = 0.6 Hz; δ_C_ 19.2) is replaced by a hydroxymethyl group (δ_H_ 4.41; δ_C_ 59.5) in **1c**. The position of the methoxyl group was also confirmed by the NOESY correlation from the methoxyl protons to H-13 (δ_H_ 7.15, s) (Figure S14). Therefore, **1c** is 1-hydroxy-12-methoxycitromycin. The literature search revealed that **1c** has never been previously reported.

**Figure 2:**
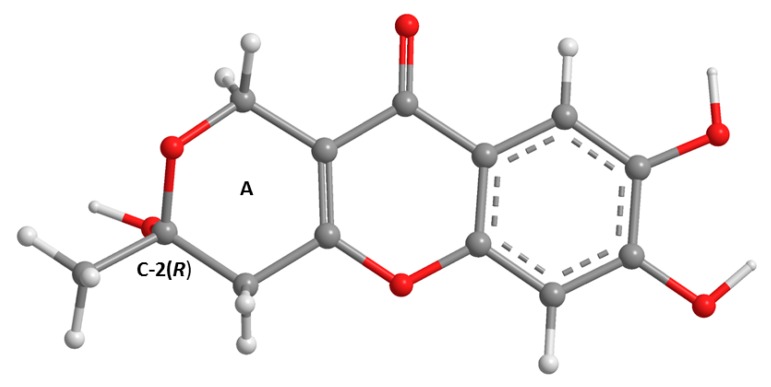
The most stable APFD/6-311+G(2d,p) conformation of **3a** (C-2*R*). The asymmetric carbon is presented with the hydroxyl group facing straight down.

The analysis of the ^1^H, ^13^C NMR (Table 2, Figures S28 and S29) and the (+)-HRESIMS spectra of **3a** revealed that its planar structure was the same as that of penialidin F, previously isolated from the culture of *Penicillium janthinellum* DT-F29, collected from marine sediments [9]. Curiously, even though the authors reported the optical rotation of penialidin F as levorotatory ([α]${}_{D}^{25}$ −4.13, *c* = 1.0, MeOH), they did not determine the absolute configuration of its stereogenic carbon (C-2). Similarly, we have also found the optical rotation of the **3a** levorotatory, ([α]${}_{D}^{25}$ −7.5, *c* = 0.04, MeOH). Since **3a** was not isolated as a suitable crystal for X-ray analysis, its calculated ECD spectrum was performed to compare with the experimental ECD spectrum. Therefore, the conformational analysis of **3a** by molecular mechanics (MM2 and MMFF95 force fields) focused on combinations of hydroxyl 120° rotations and two 3,6-dihydro-2*H*-pyran-2-ol ring conformations. A total of 30 conformations were energetically minimized and ranked using a faster DFT model (smaller basis set, APFD/6-31G). The lowest three of these, representing 99% of the model Boltzmann population, were then further energetically minimized with a larger basis set (APFD/6-311+G(2d,p)). The most stable conformation is depicted in Figure 2 and represents 64% of the Boltzmann population while the other two amount to 25% and 11%.

**Figure 3:**
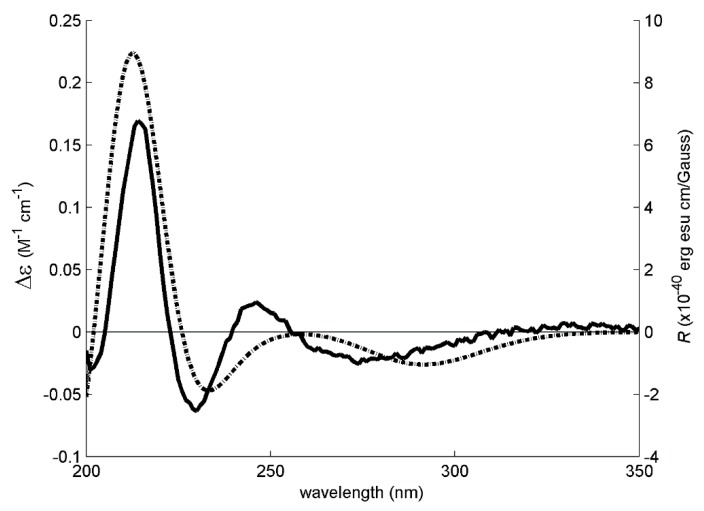
The experimental (solid line, left axis) and simulated (dotted line, right axis) ECD spectra of 3a/C-2(*R*). The ECD experimental signal was very weak, requiring the use of 40 accumulations, increased digital integration time and post-acquisition noise filtering (moving mean).

These three models were then used to calculate the expected Boltzmann-averaged ECD spectrum of **3a**’s *R* enantiomer. The good fit between the calculated and experimental ECD spectra shown in Figure 3 is enough evidence to conclude that **3a** is the *R* enantiomer. However, the weak experimental ECD signal of **3a** could indicate that this compound does not exist as a pure *R* enantiomer but as an enantiomeric mixture with an excess of the *R* enantiomer.

Compound **3b** was isolated as a 1:2 mixture (estimated from the integration of the proton signals in the ^1^H NMR spectrum) with myxotrichin C (**2b**). Based on the (+)-HRESIMS *m*/*z* 279.0878 [M + H]^+^, (calculated 277.0869 for C_14_H_15_O_6_), the molecular formula C_14_H_14_O_6_ was attributed to **3b**. The ^1^H and ^13^C NMR spectra of **3b** (a minor compound) resembled those of penialidin F (**3a**) (Table 2). The ^13^C NMR spectrum of **3b** (Table 2, Figure S22) exhibited fourteen carbon signals which, in combination with DEPTs and HSQC spectra (Figure S24), can be categorized as one conjugated ketone carbonyl (δ_C_ 173.2), six quaternary sp^2^ (δ_C_ 157.9, 152.1, 150.8, 144.4, 115.4, 113.0), two methine sp^2^ (δ_C_ 107.4 and 102.7), one ketal (δ_C_ 97.7), one oxymethylene sp^3^ (δ_C_ 57.1), one methylene sp^3^ (δ_C_ 37.1), one methyl (δ_C_ 22.4), and one methoxyl (δ_C_ 48.3) carbon. The ^1^H NMR spectrum (Figure S21) displayed two aromatic singlets at δ_H_ 7.26 (H-13) and 6.83 (H-10), two pairs of geminally coupled methylene protons at δ_H_ 4.52, dd (*J* = 14.9, 0.9 Hz)/4.22, dt, (*J* = 4.9, 2.1 Hz) and 2.63, dd (*J* = 17.6, 1.5 Hz)/2.96, dd (*J* = 1.76, 2.6 Hz), a methyl singlet at δ_H_ 1.44 and a methoxyl singlet at δ_H_ 3.21. Comparison of the ^1^H and ^13^C data of **3b** with those of **3a** (Table 2) led to the conclusion that **3b** is a methyl ketal of **3a**. This hypothesis is confirmed not only by the molecular formula of **3b**, which is 14 *amu* more than that of **3a**, but also by the HMBC correlation (Figure S25) of the singlet of the methoxyl protons to the ketal carbon at δ_C_ 97.7. Therefore, **3b** was named penialidin G.

Surprisingly, the ECD spectrum of the mixture of myxotrichin C (**2b**) and **3b** did not exhibit any Cotton effects. Consequently, we concluded that **3b** is a mixture of both enantiomers.

**Figure 4:**
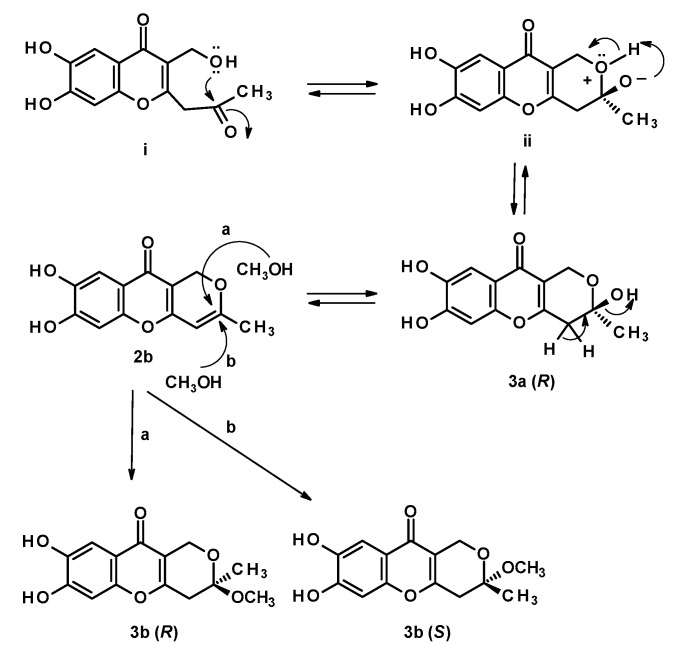
The formation of **3a**, **2b** and a pair of enantiomers of **3b** by nucleophilic addition of methanol to **2b**.

The biogenesis of **2b**, **3a**, and **3b** can be hypothesized as originated from the hexaketide intermediate (i) (Figure 4). Enzyme-catalyzed nucleophilic addition of the primary hydroxyl group to the ketone carbonyl led to a cyclization to form a 2-methyl-3,6-dihydro-2*H*-pyran-2-ol ring, through an intermediate (ii), in **3a** (2*R*). Dehydration of the hemiketal in **3a** furnished myxotrichin C (**2b**), which underwent a nucleophilic addition of methanol (chromatographic solvent) at C-2 to form an enantiomeric mixture of **3b**. Therefore, **3b** can be an artifact and not a natural product. The co-occurrence of **2b** and **3b** can be a concrete proof of this hypothesis.

Compound **4** was isolated as white crystals (mp 150–152 °C), and its molecular formula was established as C_13_H_10_O_6_ on the basis of its (+)-HRESIMS *m*/*z* 263.0569 [M + H]^+^, (calculated 263.0556 for C_13_H_11_O_6_), indicating nine degrees of unsaturation. The IR spectrum showed absorption bands for hydroxyl (3491, 3376 cm^−1^), conjugated ketone carbonyls (1679, 1661 cm^−1^), olefin (1648 cm^−1^), aromatic (1587, 1523 cm^−1^), and ether (1276 cm^−1^) groups. The ^13^C NMR spectrum (Table 3, Figure S31) displayed thirteen carbon signals which were categorized, according to the DEPTs and HSQC spectra (Figure S33), as two conjugated ketone carbonyls (δ_C_ 198.3 and 181.6), four oxyquaternary sp^2^ (δ_C_ 191.4, 156.9, 155.9, 141.9), one quaternary sp^2^ (δ_C_ 111.1), three methine sp^2^ (δ_C_ 110.3, 103.8, 103.2), one quaternary sp^3^ (δ_C_ 86.3), one oxymethylene sp^3^ (δ_C_ 69.8) and one methyl (δ_C_ 16.4) carbons. The ^1^H NMR spectrum (Table 3, Figure S31) showed two aromatic singlets at δ_H_ 7.05 and 6.41, a doublet of an olefinic proton at δ_H_ 5.68 (*J* = 0.6 Hz), a pair of doublets of the oxymethylene protons at δ_H_ 4.49 (*J* = 12.4 Hz)/4.63 (*J* = 12.4 Hz), and a methyl singlet at δ_H_ 2.31, in addition to a broad signal of the hydroxyl protons at δ_H_ 10.01. The presence of the 6,7-dihydroxy-2,3-dihydro-4*H*-1-benzopyran-4-one moiety was corroborated by the HMBC correlations (Table 3, Figure S34) from H-5 (δ_H_ 7.05, s) to C-4 (δ_C_ 181.6), C-6 (δ_C_ 141.9), C-7 (δ_C_ 155.9) and C-8a (δ_C_ 156.9); H-8 (δ_H_ 6.41, s) to C-4, C-4a (δ_C_ 111.1), C-6, C-7 and C-8a, and from H_2_-2 (δ_H_ 4.49, d, *J* = 12.4 Hz/4.63, d, 12.4 Hz) to C-4 and C-8a. That another portion of the molecule was a 5-methylfuran-3(2*H*)-one ring was substantiated by the COSY correlation (Table 3, Figure S32) from the methyl singlet at δ_H_ 2.31 to H-3′ (δ_H_ 5.68, d, *J* = 0.6 Hz), as well as the HMBC correlations (Table 3, Figure S34) from H-3′ to C-3 (δ_C_ 86.3), C-2′ (δ_C_ 191.4), C-4′ (δ_C_ 198.3), and from the methyl singlet at δ_H_ 2.31 to C-2′ and C-3′ (δ_C_ 103.8). Finally, the 5-methylfuran-3(2*H*)-one moiety and the 6,7-dihydroxy-2,3-dihydro-4*H*-1-benzopyran-4-one were connected through C-3 since the HMBC spectrum exhibited correlations from H-2 (δ_H_ 4.49, d, *J* = 12.4 Hz) to C-3 and C-4′, and from H-3′ to C-3. Therefore, the planar structure of **4** corresponds to 5′-methyl-2*H*,3′*H*,4*H*-spiro [1-benzopyran-3,2′-furan]-3′,4-dione. Since **4** was obtained as a suitable crystal, an X-ray analysis was carried out to determine the absolute configuration of the stereogenic carbon (C-3).

**Figure 5:**
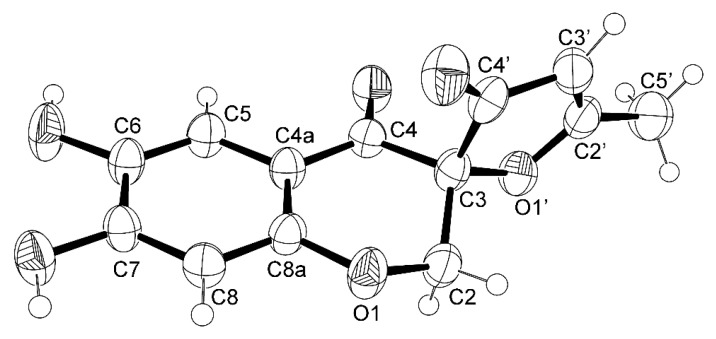
The Ortep view of **4**.

The ORTEP view, shown in Figure 5, not only confirmed the proposed structure for **4** but also determined unequivocally the absolute configuration of C-3 as 3*S*. Therefore, the absolute structure of **4** is (3*S*)-6,7-dihydroxy-5′-methyl-3′*H*,4*H*-spiro[chromene-3,2′-furan]-3′,4-dione, which was named erubescenschromone A.

Compound **5** was isolated as a white solid (mp 276–277 °C), and displayed its (+)-HRESIMS *m*/*z* at 305.0667 [M + H]^+^, (calculated 305.0661 for C_15_H_13_O_7_). Therefore, its molecular formula was established as C_15_H_12_O_7_, indicating ten degrees of unsaturation. The IR spectrum exhibited absorption bands for hydroxyl (3446 cm^−1^), conjugated ketone carbonyls (1719, 1646 cm^−1^), aromatic (1560, 1541 cm^−1^), olefin (1618 cm^−1^) and ether (1276 cm^−1^) groups. However, its ^13^C NMR spectrum (Table 4, Figure S36) displayed only fourteen carbon signals which, in combination with DEPTs and HSQC spectra (Figure S37), can be classified as two ketone carbonyls (δ_C_ 198.2 and 173.4), one conjugated carboxyl carbonyl (δ_C_ 167.1), three oxyquaternary sp^2^ (δ_C_ 157.1, 152.8, 143.2), two quaternary sp^2^ (δ_C_ 117.4 and 112.0), one oxymethine sp^2^ (δ_C_ 158.9), three methine sp^2^ (δ_C_ 134.9, 128.7, 104.0), one methoxyl (δ_C_ 61.0) and one methyl (δ_C_ 27.5) carbons. The ^1^H NMR spectrum (Table 4, Figure S35) exhibited four singlets of aromatic/olefinic protons at δ_H_ 8.73 (1H), 7.35 (2H), 7.03 (1H), one methoxyl singlet at δ_H_ 3.75 and one methyl singlet at δ_H_ 2.29. That **5** consists of a 7-hydroxy-6-methoxy-4-oxo-4*H*-chromene-5-carboxylic acid nucleus, with a substituent on C-3, was supported by the HMBC correlations (Table 4, Figure S38) from H-2 (δ_H_ 8.73) to C-4 (δ_C_ 173.4), C-8a (δ_C_ 152.8) and C-3 (δ_C_ 117.4); H-8 (δ_H_ 7.03) to C-4a (δ_C_ 112.0), C-6 (δ_C_ 143.2), C-7 (δ_C_ 157.1), and C-8a, from OCH_3_-6 (δ_H_ 3.75) to C-6, as well as the carbon chemical shift value of OCH_3_-6 (δ_C_ 61.0), characteristic of the methoxyl group flanked by one oxygenated substituent and one carboxyl group. Like many other quaternary sp^2^ carbon linked to the carboxyl substituent, the intensity of the signal of C-5 was not strong enough to be observed in the ^13^C NMR spectrum. Moreover, since there is no proton two or three bonds away from C-5, it was not possible to localize the C-5 signal in the HMBC spectrum. The existence of a 3-oxobut-1-en-1-yl substituent was supported by the presence of a singlet of two protons at δ_H_ 7.35 (H-10 and H-11) which, through the HSQC spectrum, connected to the two methine sp^2^ carbons at δ_C_ 134.9 (C-10) and δ_C_ 128.7 (C-11), as well as the HMBC correlations from H-10/H-11 to the ketone carbonyl carbon at δ_C_ 198.2 (C-12), and from the methyl singlet at δ_H_ 2.29 (H_3_-13) to C-12 and C-11. That the 3-oxobut-1-en-1-yl substituent was on C-3 was also supported by the HMBC correlations (Table 4, Figure S38) from H-10 to C-2 and C-4 as well as from H-2 to C-10. Therefore, the structure of **5** was elucidated as 7-hydroxy-6-methoxy-4-oxo-3-[3-oxobut-1-en-1-yl]-4*H*-chromene-5-carboxylic acid. The literature search revealed that **5** has never been previously reported; however its structure and NMR data were very similar to those of PI-4, a fungal metabolite first isolated by Arai et al. [16] from the mycelium of *Penicillium italicum*, a phyotoxic fungus which causes the blue-mold rot of fruits, and later by Lu et al. [17] from the crude extract of the fungus *Chaetomium indicum* (CBS.860.68). The only difference between PI-4 and **5** is the substituent on C-6 which is a hydroxyl group in the former and a methoxyl group in the latter. Therefore, **5** is identified as 7-hydroxy-6-methoxy-4-oxo-3-[(1*E*)-3-oxobut-1-en-1-yl]-4*H*-chromene-5-carboxylic acid.

**Figure 6:**
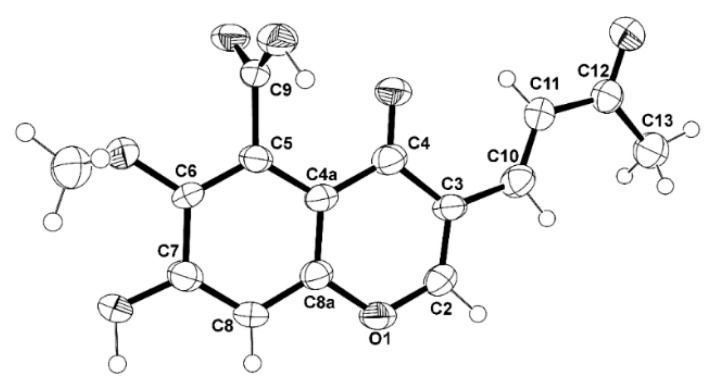
The Ortep view of **5**.

The structure of **5** and the *trans* double bond between C-10 and C-11 are confirmed by X-ray analysis, as shown in the ORTEP view in Figure 6.

Compound **6** was isolated as a pale yellow viscous oil, and its molecular formula C_26_H_20_O_11_ was established based on its (+)-HRESIMS *m*/*z* 509.1085 [M + H]^+^, (calculated 509.1084 for C_26_H_21_O_11_), indicating twelve degrees of unsaturation. The IR spectrum showed absorption bands for hydroxyl (3443 cm^−1^), ketone carbonyls (1731, 1715 cm^−1^), conjugated ketone carbonyls (1697, 1648 cm^−1^), aromatic (1634, 1556, 1596 cm^−1^), and ether (1261 cm^−1^) groups. The ^13^C NMR spectrum (Table 5, Figure S40) exhibited twenty six carbon signals which can be classified, according to the DEPTs and HSQC spectra (Table 5, Figure S42) as two ketone carbonyls (δ_C_ 204.6, 200.9), two conjugated ketone carbonyls (δ_C_ 185.3, 172.2), ten quaternary sp^2^ (δ_C_ 161.3, 156.0, 155.4, 152.5, 150.2, 144.7, 141.4, 115.1, 112.3, 109.8), four methine sp^2^ (δ_C_ 111.1, 108.5, 102.8, 102.6), two oxyquatermary sp^3^ (δ_C_ 61.9 and 78.2), two methine sp^3^ (δ_C_ 71.4 and 69.8), two methylene sp^3^ (δ_C_ 67.2 and 33.4), and two tertiary methyl (δ_C_ 32.7 and 29.3) carbons. The ^1^H NMR spectrum (Table 5, Figure S39), in combination with the HSQC spectrum, displayed four singlets of aromatic protons at δ_H_ 7.26, 7.17, 6.84 and 6.37, two methine singlets at δ_H_ 5.41 and 5.23, two doublets of the magnetically inequivalent oxymethylene protons at δ_H_ 4.36 (*J* = 12.8 Hz) and 3.59 (*J* = 12.8 Hz), two doublets of the magnetically inequivalent methylene protons at δ_H_ 3.47 (*J* = 19.2 Hz) and 2.89 (*J* = 19.2 Hz), in addition to two methyl singlets at δ_H_ 2.16 and 1.51. The presence of the 7,8-dihydroxy-3-methyl-3,4-dihydro-1*H*,10*H*-pyrano[4,3-*b*]chromen-10-one moiety was substantiated by the HMBC correlations (Table 5, Figure S43) from H-5 (δ_H_ 7.26, s; δ_C_ 108.0) to C-4 (δ_C_ 172.2), C-8a (δ_C_ 152.5), C-7 (δ_C_ 150.2), and C-6 (144.7); H-8 (δ_H_ 6.84, s; δ_C_ 102.8) to C-4, C-8a, C-7, C-6, C-4a (δ_C_ 115.1); H-12 (δ_H_ 5.41, s; δ_C_ 71.4) to C-4, C-2 (δ_C_ 161.3), C-3 (δ_C_ 112.3), C-10 (δ_C_ 78.2), and from Me-13 (δ_H_ 1.51, s; δ_C_ 29.3) to C-2, C-10, and C-9 (δ_C_ 33.4). Another portion of the molecule was identified as 3,3-disubstituted 6,7-dihydroxy-2,3-dihydro-4*H*-1-benzopyran-4-one, based on the HMBC correlations from H-5′ (δ_H_ 7.17, s; δ_C_ 111.1) to C- 4′ (δ_C_ 185.3), C-8′a (δ_C_ 156.0), C-7′ (δ_C_ 155.4), C-6′ (δ_C_ 141.4); H-8′ (δ_H_ 6.37, s; δ_C_ 102.6) to C-4′, C-8′a, C-6′ and C-4′a (δ_C_ 109.8), as well as from H_2_-2′ (δ_H_ 4.36, *J* = 12.8 Hz/3.59, *J* = 12.8 Hz) to C-4′, C-8′a and C-3′ (δ_C_ 61.9). That the disubstituted 6,7-dihydroxy-2,3-dihydro-4*H*-1-benzopyran-4-one was connected to the 7,8-dihydroxy-3-methyl-3,4-dihydro-1*H*,10*H*-pyrano[4,3-*b*]chromen-10-one moiety, through C-3′ of the former and C-12 of the latter, was confirmed by the HMBC correlations from H-12 to C-3′ and H_2_-2′ to C-12. Moreover, since the HMBC spectrum also exhibited correlations from H-12 and H_2_-2′ to the ketone carbonyl carbon at δ_C_ 200.9 (C-14), from H-15 (δ_H_ 5.23, s; δ_C_ 69.8) to C-9, C-10 (δ_C_ 78.2), C-14, and from Me-13 to C-15, the 7,8-dihydroxy-3-methyl-3,4-dihydro-1*H*,10*H*-pyrano[4,3-*b*]chromen-10-one moiety was connected through C-10 and C-15 of the oxan-4-one ring. The acetyl group on C-15 was corroborated by the HMBC correlations from Me-17 (δ_H_ 2.16, s; δ_C_ 32.7) to C-15 and the carbonyl carbon at δ_C_ 204.6 (C-16), as well as from H-15 to C-16. Taking together the molecular formula, the NMR data, and the HMBC correlations, the planar structure of **6** was unambiguously established. In order to determine the relative configurations of the stereogenic carbons C-10, C-12, C-15 and C-3′, the ROESY spectrum was obtained. The ROESY spectrum (Figure S44) exhibited strong correlations from Me-13 (δ_H_ 1.51, s) to H-15 (δ_H_ 5.23, s) and the methylene proton at δ_H_ 2.89, d (*J* = 19.2 Hz), implying that these three protons are on the same face. Additionally, H-15 also shows a correlation with Me-17 (δ_H_ 2.16, s). Since the pyran ring and the oxan-4-one ring of the 9-oxabicyclo[3.3.1]nonan-3-one ring system are in a rigid half-chair conformation, Me-13 must be in a pseudoequatorial position while the methylene proton at δ_H_ 2.89, d (*J* = 19.2 Hz) and H-15 are in a pseudoaxial position. Therefore, the acetyl group on C-15 must be in a pseudoequatorial position. This was confirmed by the higher chemical shift value (δ_H_ 3.47, d, *J* = 19.2 Hz) of the pseudoequatorial H-9 as it is in the deshielding zone of the carbonyl (C-16) of the acetyl group. On the other hand, H-12 (δ_H_ 5.41, s) showed a weak correlation to one of H-2′ at δ_H_ 3.59, d (*J* = 12.9 Hz). Therefore both of these protons should be in the pseudoequatorial position since the pseudoaxial H-2′ (δ_H_ 4.36, d, *J* = 12.9 Hz) is under the anisotropic effect (deshielding) of the carbonyl at C-14 of ring D. With these ROESY correlations, the relative configurations of C-10, 12, 15, and 3′ were proposed as 10S*, 12S*, 15S*, and 3′S*. However, it is necessary to determine the absolute configurations of these stereogenic carbons.

**Figure 7:**
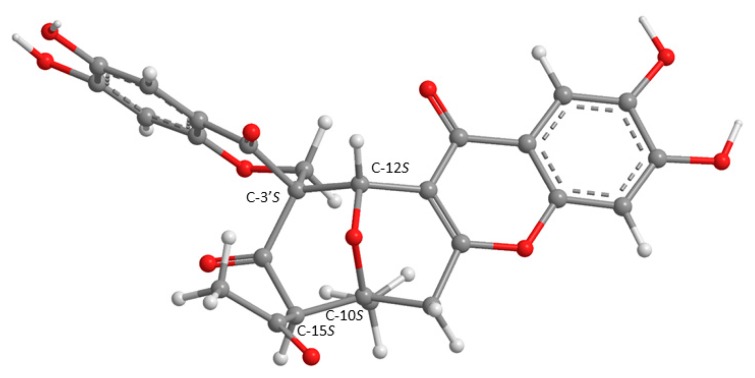
The most stable APFD/6-31G conformation of **6**, presented with the absolute configuration found by spectrometric methods.

Since **6** could not be obtained as a suitable crystal for an X-ray analysis, the determination of its stereogenic carbons had to be carried out by comparison of the calculated and experimental ECD spectra. Although the ROESY correlations pointed to the relative configuration of C-10 and C-15 as 10*S*, 15*S*, it is possible that it can be 10*R*, 15*R*, thus reducing its number of possible configurations from 16 (eight pairs of diastereoisomers) to eight (four pairs of diastereoisomers). Hence, four computational models were constructed by combining the two configurations of C-3′ with the two of C-12. The conformational analysis of **6** by molecular mechanics (MM2 and MMFF95 force fields) focused on combinations of hydroxyl 120° rotations and rings conformations. Most diastereoisomers did not show ring conformational freedom which limited the number of models to compute. The most stable APFD/6-31G conformation of **6** whose absolute configurations of C-10, C-12, C-15, and C-3′ are 10*S*, 12*S*, 15*S*, 3′*S*, as deduced from ROESY correlations, is shown in Figure 7.

**Figure 8:**
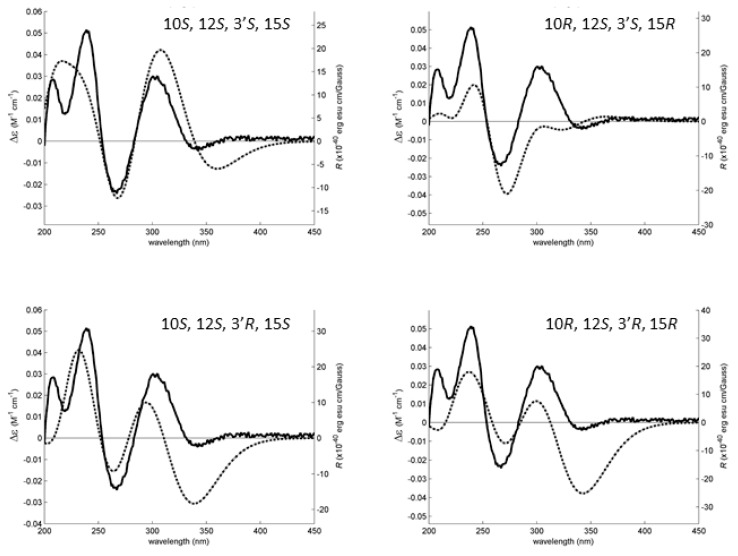
The experimental (solid line, left axes) and simulated (dotted line, right axes) ECD spectra of four diastereoisomers of **6**. The best experimental-simulated fit belongs to the diastereoisomer with the absolute configuration 10*S*, 12*S*, 3′*S*, 15*S*. The theoretical ECD spectra of the enantiomers of the presented diastereoisomers are the exact inversions of the ones depicted here and do not fit the experimental data.

All conformations were energetically minimized and ranked using a DFT model. The lowest energy ones, representing at least 95% of the model Boltzmann population, were used to calculate the expected Boltzmann-averaged ECD spectra of the four **6** diastereoisomers. The fitting between the experimental and calculated spectra is presented in Figure 8, showing that the **6** is the C-10*S*, C-12*S*, C-3′*S*, C-15*S* enantiomer.

The literature search revealed that **6** has never been previously reported and therefore it is a new compound which was named erubescenschromone B.

Compound **7** was isolated as a pale yellow oil and the (+)-HRESIMS showed the [M + H]^+^ peak at *m*/*z* 509.1085 (calculated 509.1084 for C_26_H_21_O_11_). Therefore, its molecular formula is C_26_H_20_O_11_, indicating eighteen degrees of unsaturation. The IR spectrum showed absorption bands for hydroxyl (3491, 3376 cm^−1^), conjugated ketone carbonyls (1679, 1661 cm^−1^), olefin (1648 cm^−1^), aromatic (3108, 1578, 1523 cm^−1^), and ether (1206 cm^−1^) groups. The ^13^C NMR spectrum (Table 6, Figure S46) exhibited twenty six carbon signals which, in combination with DEPTs and HSQC spectra (Table 6, Figure S47), can be classified as three carbonyls (δ_C_ 202.8, 183.6, 173.5), fifteen quaternary sp^2^ (δ_C_ 172.6, 155.9, 154.9, 154.2, 152.1, 150.7, 144.3, 141.6, 138.0, 132.4, 129.5, 118.6, 113.5, 1119, 103.9), five methine sp^2^ (δ_C_ 125.7, 110.5, 108.7, 103.3, 103.1), one methylene sp^3^ (δ_C_ 66.2) and two methyl (δ_C_ 32.4 and 16.6) carbons. The ^1^H NMR spectrum (Table 6, Figure S45) exhibited five singlets of aromatic protons at δ_H_ 8.00, 7.45, 7.19, 6.93, and 6.34; a singlet of oxymethylene protons at δ_H_ 4.67 (2H) and two methyl singlets at δ_H_ 2.71 and 2.32. The presence of the 6,7-dihydroxy-2,3-dihydro-4*H*-chromen-4-one moiety was corroborated by the HMBC correlations (Table 6, Figure S48) from H-5′ (δ_H_ 7.19, s; δ_C_ 110.5) to C-4′ (δ_C_ 183.6), C-6′(δ_C_ 155.9), C-8′a (δ_C_ 154.9) and C-7′ (δ_C_ 141.6); H-8′ (δ_H_ 6.34, s; δ_C_ 103.3) to C-4′a (δ_C_ 111.9), C-6′, C-7′, C-8′a, and H_2_-2′ (δ_H_ 4.67, s; δ_C_ 66.2) to C-3′ (δ_C_ 103.9) and C-4′ (δ_C_ 183.6). That the substituent on C-3′ was an enolic exocyclic double bond was substantiated by the HMBC correlations from H_2_-2′ to C-3′ (δ_C_ 103.9) and the enolic carbon (C-9′, δ_C_ 172.6). Another moiety was established as 7-substituted 5-acetyl-2,3-dihydroxy-6-methyl-9*H*-xanthen-9-one since the HMBC spectrum showed correlations from H-5 (δ_H_ 6.93, s; δ_C_ 103.1) to C-7 (δ_C_ 143.3), C-8a (δ_C_ 113.5), C-10a (δ_C_ 154.2); H-8 (δ_H_ 7.45, s; δ_C_ 108.7) to C-6 (δ_C_ 150.7), C-7, C-9 (δ_C_ 173.5), C-10a; H-1 (δ_H_ 8.00, s; δ_C_ 125.7) to C-3 (δ_C_ 138.0) and C-4a (δ_C_ 152.1); Me-11 (δ_H_ 2.32, s; δ_C_ 16.6) to C-2 (δ_C_ 129.5), C-3, C-4 (δ_C_ 132.2). Since H-1 also showed the HMBC correlation to C-9′, the 5-acetyl-2,3-dihydroxy-6-methyl-9*H*-xanthen-9-one was linked to the 6,7-dihydroxy-2,3-dihydro-4*H*-chromen-4-one moiety through C-9′. An extensive literature search revealed that the structure of **7** is the same as that of the enol tautomer of the compound, named SPF-3059-30, isolated from the acetone extract of the mycelium of *Penicillium* sp. SPF-3050 (FERM BB-7663), cultured in the liquid medium [11]. However, the authors claimed that SPF-3050-30 was isolated as a mixture of keto-enol tautomers, as supported by the duplication of the ^1^H and ^13^C chemical shift values but without an assignment. The ^13^C NMR data of SPF-3050-30 displayed forty one carbon signals, e.g., four signals for the methyl groups, three signals for the oxymethylene carbons, two signals for the carbonyl carbon of the acetyl group, two signals for the carbonyl of the chromone nucleus and two signals of the carbonyl of the xanthone moiety, etc., while its ^1^H NMR data presented two methyl signals of the methyl group on the xanthone nucleus, two methyl signals for the acetyl group and nine signals of aromatic protons. On the contrary, the ^1^H and ^13^C NMR spectra of **7** in DMSO (Table 6, Figures S45 and S46) showed that it was present only in an enolic form. This is supported by the fact that the enolic form is stabilized by the hydrogen bonding between OH-9′ and the carbonyl of the chromone moiety (C-4′).

Compound **7** can be considered as a decarboxylated derivative of xanthofulvin, a semaphorin inhibitor isolated from the culture broth of the fungus *Penicillium* sp. SPF-3059 [18].

Compounds **1a**–**e**, **2a**, **3a**, **4**, **7**–**9** were evaluated for their antibacterial activity against Gram-negative and Gram-positive bacteria by disc diffusion method, and the MIC and MBC of several reference strains and multidrug-resistant isolates from the environment were also determined. In the disc diffusion assay, a halo of growth inhibition for all Gram-positive bacteria exposed to **8** (Table 7) and for methicillin-resistant *Staphylococcus aureus* (MRSA) 66/1 exposed to **9** was detected. However, in the range of concentrations tested, it was only possible to determine MICs for **8** (Table 7), with MIC values of 8 mg/mL for *E. faecalis* ATCC 29212 and vancomycin-resistant *E. faecalis* (VRE) B3/101, 16 mg/mL for *E. faecium* ATCC 19434, and 32 mg/mL for *E. faecium* 1/6/63 (VRE) and *S. aureus* ATCC 29213. While it was not possible to determine the MBC for the other Gram-positive strains, the MBC for *S. aureus* ATCC 29213 was 64 mg/mL (Table 7). These results suggested that **8** might have a bacteriostatic effect.

The ability of the tested compounds to prevent biofilm formation was evaluated on four reference strains by measuring the total biomass. For **8**, four concentrations ranging from 2 × MIC to ¼ MIC were tested against *E. faecalis* ATCC 29212, *E. faecium* ATCC 19434 and *S. aureus* ATCC 29213. For the other compounds, since it was not possible to determine their MIC values, the highest concentration tested in the previous assays was used. The results were interpreted using a comparative classification that divides adherence capability of tested strains into four categories: (i) non-adherent, (ii) weakly adherent, (iii) moderately adherent, and (iv) strongly adherent [19]. OThe optical density cut-off value (ODc) for each microtiter plate was defined as three standard deviations above the mean OD of the negative control. The use of this classification, which uses the negative control as the starting point instead of using the positive control as a reference, reduces the risk of inconsistencies due to external factors that influence biofilm production [20]. The tested compounds did not inhibit the biofilm formation of *S. aureus* ATCC 29213, *E. coli* ATCC 25922, and *P. aeruginosa* ATCC 27853. However, the biofilm forming ability of *E. faecalis* ATCC 29212, which is classified as a strong biofilm producer, was impaired by **8** (MIC and 2 × MIC) and **9** (Table 8). On the other hand, **8** was able to increase the biofilm production of a weak biofilm producer *E. faecium* ATCC 19434.

The screening of a potential synergy between the tested compounds and clinically relevant antimicrobial drugs revealed a slight synergy, as determined by the disc diffusion assay (Table 9). Compound **1b**, in combination with cefotaxime (CTX), resulted in a small synergistic effect, as seen by a small increment in the zone of inhibition when compared to the inhibition halo of CTX alone in *E. coli* SA/2, an extended-spectrum β-lactamase producer (ESBL). A similar effect was observed for VRE *E. faecalis* B3/101 when **8** was combined with VAN. These results were confirmed by the checkerboard method or by determining the MIC for each antibiotic in the presence of a fixed concentration of each compound when it was not possible to determine a MIC value for the test compound. In the latter, the concentration of each compound used was the highest concentration tested in previous assays which did not inhibit the growth of the four multidrug-resistant strains under study. The effects observed using the disc diffusion assay were not replicated, however, when VRE *E. faecalis* B3/101 was exposed to **1d**, **3a** and **9**, there was a two-fold reduction in the MIC of VAN. On the other hand, when ESBL *E. coli* SA/2 was exposed to **1c** and **7**, there was at least a two-fold increase in the MIC of CTX. When VRE *E. faecium* 1/6/63 was exposed to **9**, there was a two-fold reduction in the MIC of VAN. On the contrary, when it was exposed to **1e**, there was at least a two-fold increase in the MIC of VAN (Table 9). The differences in the results obtained using both techniques may be explained by different diffusion rates of each compound in the agar plates.

Thus, in terms of antibacterial activity, **8** is the most promising. Even though no synergy with VAN or OXA was found, this compound alone exhibited an antibiofilm activity against *E. faecalis* and antibacterial activity against the reference *S. aureus*, *E. faecalis*, and *E. faecium* strains. Most importantly, **8** showed antibacterial activity against both vancomycin-resistant *E. faecalis* and vancomycin-resistant *E. faecium* strains, a pathogen classified by WHO as high priority for the research and development of new antibiotics [21]. These results call for a more in-depth study of this compound.

## 3. Experimental Section

### 3.1. General Experimental Procedures

The melting points were determined on a Stuart Melting Point Apparatus SMP3 (Bibby Sterilin, Stone, Staffordshire, UK) and are uncorrected. Optical rotations were measured on an ADP410 Polarimeter (Bellingham + Stanley Ltd., Tunbridge Wells, Kent, UK). Infrared spectra were recorded in a KBr microplate in an FTIR spectrometer Nicolet iS10 from Thermo Scientific (Waltham, MA, USA) with a Smart OMNI-Transmission accessory (Software 188 OMNIC 8.3, Thermo Scientific, Waltham, MA, USA). ^1^H and ^13^C NMR spectra were recorded at ambient temperature on a Bruker AMC instrument (Bruker Biosciences Corporation, Billerica, MA, USA) operating at f300 or 500 and 75 or 125 MHz, respectively. High resolution mass spectra were measured with a Waters Xevo QToF mass spectrometer (Waters Corporations, Milford, MA, USA) coupled to a Waters Aquity UPLC system. A Merck (Darmstadt, Germany) silica gel GF_254_ was used for preparative TLC, and a Merck Si gel 60 (0.2–0.5 mm) was used for column chromatography.

### 3.2. Fungal Material

The fungus was isolated from the marine sponge *Neopetrosia* sp. which was collected, by scuba diving at a depth of 5–10 m, from the coral reef at Samaesan Island (12°34′36.64″ N, 100°56′59.69″ E), Chonburi province, Thailand, in April 2014. The sponge was washed with 0.01% sodium hypochlorite solution for 1 min, followed by sterilized seawater three times, and then dried on sterile filter paper under sterile aseptic condition. The sponge was cut into small pieces (5 mm × 5 mm) and placed on Petri dish plates containing 15 mL potato dextrose agar (PDA) medium mixed with 300 mg/L of streptomycin sulfate, and incubated at 28 °C for 7 days. The hyphal tips emerging from sponge pieces were individually transferred onto PDA slants and maintained as pure cultures at Kasetsart University Fungal Collection, Department of Plant Pathology, Faculty of Agriculture, Kasetsart University, Bangkok, Thailand, for further identification. The fungal strain KUFA0220 was identified as *Penicillium erubescens*, based on morphological characteristics such as colony growth rate and growth pattern on standard media, namely Czapek’s agar, Czapek yeast autolysate agar, and malt extract agar. Microscopic characteristics including size, shape and ornamentation of conidiophores and spores were examined under a light microscope. This identification was confirmed by molecular techniques using Internal Transcribed Spacer (ITS) primers. DNA was extracted from young mycelia following a modified Murray and Thompson method [22]. Primer pairs ITS1 and ITS4 [23] were used for ITS gene amplification. PCR reactions were conducted on Thermal Cycler and the amplification process consisted of the initial denaturation at 95 °C for 5 min, 34 cycles at 95 °C for 1 min (denaturation), at 55 °C for 1 min (annealing) and at 72 °C for 1.5 min (extension), followed by final extension at 72 °C for 10 min. The PCR products were examined by agarose gel electrophoresis (1% agarose with 1 × TBE buffer) and visualized under UV light after staining with ethidium bromide. DNA sequencing analyses were performed using the dideoxyribonucleotide chain termination method [24] by Macrogen Inc. (Seoul, Korea). The DNA sequences were edited using the FinchTV software (version 1.4, Geospiza Inc, Seattle, WA, USA) and submitted into the BLAST program for alignment and compared to fungal species in the NCBI database (http://www.ncbi.nlm.nih.gov/). Its gene sequences were deposited in GenBank with accession number KY041867.

### 3.3. Extraction and Isolation

The fungus was cultured for one week at 28 °C in five Petri dishes (i.d. 90 mm) containing 20 mL of potato dextrose agar per dish. The mycelial plugs (5 mm in diameter) were transferred to two 500 mL Erlenmeyer flasks containing 200 mL of potato dextrose broth, and incubated on a rotary shaker at 120 rpm at 28 °C for one week. Fifty 1000 mL Erlenmeyer flasks, each containing 300 g of cooked rice, were autoclaved at 121 °C for 15 min. After cooling to room temperature, 20 mL of a mycelial suspension of the fungus was inoculated per flask and incubated at 28 °C for 30 days, after which 500 mL of ethyl acetate was added to each flask of the moldy rice and macerated for 7 days, and then filtered with Whatman No. 1 filter paper (GE Healthcare UK Limited, Buckinghamshire, UK). The ethyl acetate solutions were combined and concentrated under reduced pressure to yield 160 g of crude ethyl acetate extract which was dissolved in 500 mL of CHCl_3_ and then filtered with Whatman No. 1 filter paper. The chloroform solution was then washed with H_2_O (3 × 500 mL) and dried with anhydrous Na_2_SO_4_, filtered and evaporated under reduced pressure to give 112 g of the crude chloroform extract which was applied on a column of silica gel (450 g), and eluted with mixtures of petrol-CHCl_3_ and CHCl_3_-Me_2_CO, wherein 250 mL fractions were collected as follow: Frs 1–147 (petrol-CHCl_3_, 1:1), 148–223 (petrol-CHCl_3_, 3:7), 224–230 (petrol-CHCl_3_, 1:9), 231–238 (CHCl_3_), 239–452 (CHCl_3_-Me_2_CO, 9:1), 453–512 (CHCl_3_-Me_2_CO, 7:3), 512–546 (Me_2_CO, 7:3). Frs 75–117 were combined (1.18 g) and applied on a column of silica gel (35 g) and eluted with mixtures of petrol-CHCl_3_ and CHCl_3_-Me_2_O, wherein 100 mL sfrs were collected as follow: Sfrs 1–20 (petrol), 21–33 (petrol-CHCl_3_, 9:1), 34–48 (petrol-CHCl_3_, 7:3), 49–59 (petrol-CHCl_3_, 1:9), 60–65 (petrol-CHCl_3_), 66–80 (CHCl_3_-Me_2_CO, 9:1), 81–106 CHCl_3_-Me_2_CO, 7:3), 107–120 (Me_2_CO). Sfrs 35–46 were combined (103.0 mg) and purified by TLC (Silica gel G_254_, CHCl_3_:Me_2_CO:HCO_2_H, 97:3:0.01) to give 50.2 mg of β-sitostenone [3]. Frs 238–245 were combined (1.75 g) and precipitated in MeOH to give 202.1 mg of **8** [12,13,14]. Frs 246–251 were combined (2.67 g) and precipitated in MeOH to give 472.2 mg of ergosterol 5,8-endoperoxide [4]. Frs 252–286 were combined (493.0 mg) and crystallized in MeOH to give further 367.1 mg of ergosterol 5,8-endoperoxide. Frs 287–299 were combined (580.4 mg) and crystallized in a mixture of CHCl_3_-Me_2_CO to give 78 mg of **2a** [7,8], and the mother liquor was combined with frs 300–319 (837.2 mg) and precipitated in Me_2_CO to give 10.0 mg of **1b** [5]. The mother liquor (855 mg) was applied on a column chromatography of silica gal (30 g) and eluted with petrol-CHCl_3_, CHCl_3_, CHCl_3_-Me_2_CO, and MeOH, wherein 100 mL fractions were collected as follows: Sfrs 1–11 (petrol-CHCl_3_, 1:1), 12–28 (petrol-CHCl_3_, 3:7), 29–86 (petrol-CHCl_3_, 1:9), 87–126 (CHCl_3_), 127–135 (CHCl_3_-Me_2_CO, 9:1), 136–138 (Me_2_CO). Sfrs 100–126 were combined (71.3 mg) and purified by TLC (Silica gel G_254_, CHCl_3_:Me_2_CO:HCO_2_H, 9:1:0.01) to give further 10.0 mg of **1b**. Sfrs 127 (48 mg) was crystallized in a mixture of CHCl_3_-Me_2_CO to give further 30.0 mg of **2a**. Frs 343–366 were combined (1.46 g) and crystallized in MeOH to give 98.3 mg of **4**, and the mother liquor was combined with frs 367–386 (1.77 g) and recrystallized in MeOH to give further 8.0 mg of **1a** [5]. The mother liquor of the combined frs 343–386 (1.36 g) was applied on a column chromatography of silica gel (40 g), and eluted with petrol-CHCl_3_, CHCl_3_, CHCl_3_-Me_2_CO and Me_2_CO, wherein 100 mL fractions were collected as follows, Sfrs 1–50 (petrol-CHCl_3_, 1:1), 51–88 (petrol-CHCl_3_, 3:7), 89–110 (petrol-CHCl_3_; 1:9), 111–139 (CHCl_3_), 140–197 (CHCl_3_-Me_2_CO, 9:1), 198–201 (CHCl_3_-Me_2_CO, 7:3), 202–215 (Me_2_CO). Sfrs 140–143 were combined (361.0 mg) and applied on a Sephadex LH-20 column (10 g), and eluted with MeOH to give 12.0 mg of **1a** and 10.1 mg of **9** [3]. Sfrs 147–151 were combined (221.0 mg) and applied on a Sephadex LH-20 column (10 g) and eluted with MeOH to give 10.0 mg of a mixture of **2b** (major component) [6] and **3b**. Sfrs 189–201 were combined (72.3 mg) and purified by TLC (Silica gel G_254_, CHCl_3_:MeOH:HCO_2_H, 95: 5: 0.1) to give 7.1 mg of **3a** [9]. Frs 387–444 were combined (2.73 g) and applied on a column of Sephadex LH-20 (20 g) and eluted with MeOH, wherein 20 mL of 30 fractions were collected. Sfrs 11–30 were combined (472.3 mg) and crystallized in Me_2_CO to give further 19 mg of **9**. The mother liquor was applied on a Sephadex LH-20 column (20 g) and eluted with a 1:1 mixture of MeOH-CHCl_3_ to give 15 mg of **7** [11]. Frs 517–529 were combined (1.40 g) and crystallized in MeOH to give 26.6 mg of **1e** [5], and the mother liquor was combined with frs 445–516 (6.90 g) and applied on a column of Sephadex LH-20 column (30 g) and eluted with MeOH, wherein 20 mL fractions were collected. Sfrs 21–30 were combined (106.2 mg) and purified by TLC (Silica gel G_254_, CHCl_3_:MeOH:HCO_2_H, 9:1:0.01) to give 10 mg of **3a** [9,10] and 12 mg of **6**. Sfrs 31–60 were combined (5.90 g) and applied on a column chromatography of silica gal (110 g) and eluted with petrol-CHCl_3_, CHCl_3_, CHCl_3_-Me_2_CO and Me_2_CO, wherein 100 mL fractions were collected as follows, Sfrs 1–26 (petrol-CHCl_3_, 1:1), 27–56 (petrol-CHCl_3_, 3:7), 57–98 (petrol-CHCl_3_, 1:9), 99–200 (CHCl_3_), 201–297 (CHCl_3_-Me_2_CO, 9:1), 298–320 (CHCl_3_-Me_2_CO, 7:3), 321–332 (CHCl_3_-Me_2_CO, 1:9), 333–358 (Me_2_CO). Sfrs 156–184 were combined (112.0 mg) and crystallized in Me_2_CO to give further 26.1 mg of **9**. Sfrs 252–294 were combined (464.9 mg) and applied on a Sephadex LH-20 column (20 g) and eluted with MeOH to give 23.0 mg of **1c**. Sfrs 295–344 were combined (3.0 g), applied on a Sephadex LH-20 column (20 g) and eluted with a 1:1 mixture of MeOH-CHCl_3_, wherein 20 mL fractions were collected. Sfrs 31–72 were combined (262.9 mg) and purified by TLC (Silica gel G_254_, CHCl_3_:MeOH:HCO_2_H, 9:1:0.01) to give 12.1 mg **1d** [6]. Sfrs 73–96 were combined (90.6 mg) and purified by TLC (Silica gel G_254_, CHCl_3_:MeOH:HCO_2_H, 9:1:0.01) to give 10.6 mg of **2c** [9]. Sfrs 97–115 were combined (644.8 mg) and precipitated in MeOH to give 12 mg of a mixture of **2b** and **3b**, and the mother liquor was dried (619.7 mg) and applied on a Sephadex LH-20 column (10 g) and eluted with a 1:1 mixture of CHCl_3_:MeOH, wherein 70 sub-fractions (2 mL each) were collected. Sfrs 25–32 were combined (40.8 mg) and precipitated in MeOH to give 10 mg of **1e** [5]. Sfrs 33–45 were combined (70.1 mg) and purified by TLC (Silica gel G_254_, CHCl_3_:MeOH:HCO_2_H, 9:1:0.01) to give 6 mg of **5**. Sfrs 46–56 were combined (65.3 mg) and precipitated in MeOH to give further 7 mg of **5**.

#### 3.3.1. 1-Hydroxy-12-methoxycitromycin (**1c**)

White solid, mp 232–233 °C (CHCl_3_/MeOH); IR (KBr) ν_max_ 3420 (br), 2921, 1662, 1627, 1594, 1555, 1517, 1453, 1270 cm^−1^; For ^1^H and ^13^C spectroscopic data (DMSO-*d*_6_, 500.13 and 125.4 MHz), see Table 1; (+)-HRESIMS *m*/*z* 277.0715 [M + H]^+^ (calculated for C_14_H_13_O_6_, 277.0712).

#### 3.3.2. Erubescenschromone A [(3*S*)-6,7-Dihydroxy-5′-methyl-3′*H*,4*H*-spiro[chromene-3,2′-furan]-3′,4-dione (**4**)]

White crystal, mp 150–152 °C (CHCl_3_/MeOH); [α${]}_{D}^{23}$ −40.0 (*c* 0.05, CDCl_3_); IR (KBr) ν_max_ 3491, 3376, 3108, 2969, 1679, 1661, 1648, 1578, 1523, 1479, 1276 cm^−1^; For ^1^H and ^13^C spectroscopic data (DMSO-*d*_6_, 500.13 and 125.4 MHz), see Table 3; (+)-HRESIMS *m*/*z* 263.0596 [M + H]^+^ (calculated for C_13_H_11_O_6_, 263.0556).

#### 3.3.3. 7-Hydroxy-6-methoxy-4-oxo-3-[(1*E*)-3-oxobut-1-en-1-yl]-4*H*-chromene-5-carboxylic Acid (**5**)

White crystal, mp 276–277 °C (CHCl_3_/MeOH); IR (KBr) ν_max_ 3446, 2922, 1719, 1646, 1618, 1560, 1541, 1521, 1276 cm^−1^; For ^1^H and ^13^C spectroscopic data (DMSO, 500.13 and 125.4 MHz), see Table 4; (+)-HRESIMS *m*/*z* 305.0667 [M + H]^+^ (calculated for C_15_H_13_O_7_, 305.0661).

#### 3.3.4. Erubescenschromone B (**6**)

Yellowish oil; [α${]}_{D}^{23}$ −150.0 (*c* 0.04, MeOH); IR (KBr) ν_max_ 3443 (br), 2922, 1731, 1715, 1697, 1648, 1634, 1556, 1540, 1506, 1261 cm^−1^; For ^1^H and ^13^C spectroscopic data (DMSO-*d*_6_, 500.13 and 125.4 MHz), see Table 5; (+)-HRESIMS *m*/*z* 509.1085 [M + H]^+^ (calculated for C_26_H_21_O_11_, 509.1084).

#### 3.3.5. SPF-3059-30 (**7**)

Yellowish oil; IR (KBr) ν_max_ 3491, 3376, 3108, 2969, 1679, 1661, 1648, 1578, 1523, 1479, 1276 cm^−1^; For ^1^H and ^13^C spectroscopic data (DMSO-*d*_6_, 500.13 and 125.4 MHz), see Table 6; (+)-HRESIMS *m*/*z* 491.0974 [M + H]^+^ (calculated for C_26_H_19_O_10_, 491.0978).

### 3.4. Electronic Circular Dichroism (ECD)

#### Electronic Circular Dichroism (ECD) of **3a** and **6**

The ECD spectra of **3a** and **6** (1.5 mM in methanol) were obtained in a Jasco J-815 CD spectropolarimeter (Jasco, Mary’s Court, Easton, MD, USA) with a 0.01 mm cell (40 accumulations for **3a**). The dihedral driver and MMFF95 minimizations were done in Chem3D Ultra (Perkin-Elmer Inc., Waltham, MA, USA). All DFT minimizations with model chemistries APFD/6-31G and APFD/6-311+G(2d,p) [25] as well as ECD spectral calculations (TD-APFD) were performed with Gaussian 16W (Gaussian Inc., Wallingford, CT, USA) using an IEFPCM solvation model for methanol. The simulated spectral lines for **3a** (Figure 3) and **6** (Figure 8) were obtained by summation of Gaussian curves, as recommended in Reference [26]. A line broadening of 0.3 eV was applied to all transitions to generate the calculated line.

### 3.5. X-ray Crystal Structures

#### 3.5.1. X-ray Crystal Structure of **4**

A single crystal of **4** was mounted on a cryoloop using paratone. X-ray diffraction data were collected at 290 K with a Gemini PX Ultra equipped with CuK_α_ radiation (λ = 1.54184 Å). The crystal was monoclinic, space group *P*2_1_/*n*, cell volume 1245.43(7) Å^3^ and unit cell dimensions *a* = 12.3445(4) Å, *b* = 7.8088(3) Å and *c* = 12.9397(5) Å and angle β = 93.165(3)° (uncertainties in parentheses). There are two molecules in the asymmetric unit, one Erubescenschromone A molecule and one water molecule, and the calculated crystal density is 1.495 g/cm^−3^. The structure was solved by direct methods using SHELXS-97 and refined with SHELXL-97 [27]. Carbon and oxygen atoms were refined anisotropically. Hydrogen atoms were directly found from difference Fourier maps and were refined freely with isotropic displacement parameters. The refinement converged to R (all data) = 6.32% and wR2 (all data) = 11.26%.

Full details of the data collection and refinement and tables of atomic coordinates, bond lengths and angles, and torsion angles have been deposited with the Cambridge Crystallographic Data Centre (CCDC 1856735).

#### 3.5.2. X-ray Crystal Structure of **5**

A single crystal of **5** was mounted on a cryoloop using paratone. X-ray diffraction data were collected at 290 K with a Gemini PX Ultra equipped with CuK_α_ radiation (λ = 1.54184 Å). The crystal was monoclinic, space group *P*2_1_/*c*, cell volume 1324.77(16) Å^3^ and unit cell dimensions *a* = 11.6888(8) Å, *b* = 7.7695(4) Å and *c* = 14.9560(12) Å and angle β = 102.748(7)° (uncertainties in parentheses). The calculated crystal density was 1.525 g·cm^−3^. The structure was solved by direct methods using SHELXS-97 and refined with SHELXL-97 [27]. Carbon and oxygen atoms were refined anisotropically. Hydrogen atoms from one of the methyl groups were placed at their idealized positions using appropriate HFIX instructions in SHELXL and included in subsequent refinement cycles, all the others were directly found from difference Fourier maps and were refined freely with isotropic displacement parameters. The refinement converged to R (all data) = 12.24% and wR2 (all data) = 14.96%.

Full details of the data collection and refinement and tables of atomic coordinates, bond lengths and angles, and torsion angles have been deposited with the Cambridge Crystallographic Data Centre (CCDC 1859409).

### 3.6. Antibacterial Activity Bioassays

#### 3.6.1. Bacterial Strains and Growth Conditions

Gram-positive bacteria included *Staphylococcus aureus* ATCC 29213, *Enterococcus faecium* ATCC 19434, *Enterococcus faecalis* ATCC 29212, methicillin-resistant *Staphylococcus aureus* (MRSA) 66/1 isolated from public buses [28], and vancomycin-resistant enterococci (VRE) *Enterococcus faecium* 1/6/63 and *Enterococcus faecalis* B3/101 isolated from river water [29]. Gram-negative strains comprised *Escherichia coli* ATCC 25922, *Pseudomonas aeruginosa* ATCC 27853 and the clinical isolate SA/2, an extended-spectrum β-lactamase producer (ESBL). All strains were kept in Trypto-Casein Soy agar (TSA—Biokar Diagnostics, Allone, Beauvais, France) slants, at room temperature, in the dark. Before each assay, all strains were cultured in Mueller-Hinton agar (MH-Biokar Diagnostics, Allone, Beauvais, France) and incubated overnight at 37 °C. Stock solutions of the compounds were prepared in dimethyl sulfoxide (DMSO—Alfa Aesar, Kandel, Germany) and kept at −20 °C. With the exception of **1e**, 10 mg/mL stock solutions were prepared. Compound **1e** was less soluble in DMSO than other compounds, so a 2 mg/mL stock solution was prepared. In the experiments, the final concentration of DMSO in the medium was below 1%, as recommended by the Clinical and Laboratory Standards Institute [30].

#### 3.6.2. Antimicrobial Susceptibility Testing

The antimicrobial activity of the compounds was screened using the Kirby-Bauer method, as recommended by the CLSI [31]: 6 mm blank paper discs (Liofilchem, Roseto degli Abruzzi TE, Italy) were impregnated with 15 µg of each compound, and the blank paper discs impregnated with DMSO were used as negative control. MH inoculated plates were incubated for 18–20 h at 37 °C. The results were evaluated by measuring the inhibition halos. The minimal inhibitory concentration (MIC) was performed in accordance with the recommendations of the CLSI [32]. Two-fold serial dilutions of the compounds were prepared in cation-adjusted Mueller-Hinton broth (CAMHB—Sigma-Aldrich, St. Louis, MO, USA) within the concentration range 64–0.063 mg/L, except for **1e**, for which the highest concentration tested was 32 mg/L. Colony forming unit counts of the inoculum were conducted in order to determine the initial inoculum size (which should be approximately 5 × 10^5^ CFU/mL). The 96-well U-shaped untreated polystyrene microtiter plates were incubated for 16–20 h at 37 °C and the MIC was determined as the lowest concentration of compound that prevented visible growth. The minimal bactericidal concentration (MBC) was determined by spreading 100 µL of the content of the wells with no visible growth on the MH plates. The MBC was determined as the lowest concentration of compound that killed 99.9% of the initial inoculum after overnight incubation at 37 °C [33]. These assays were conducted for reference and multidrug-resistant strains.

#### 3.6.3. Biofilm Formation Inhibition Assay

The effect of all compounds on biofilm formation was evaluated using the crystal violet method, as follows: the highest concentration of the tested compound in the MIC assay was added to bacterial suspensions of 1 × 10^6^ CFU/mL prepared in unsupplemented Tryptone Soy broth (TSB—Biokar Diagnostics, Allone, Beauvais, France) or TSB supplemented with 1% (*p*/*v*) glucose [d-(+)-Glucose anhydrous for molecular biology, PanReac AppliChem, Barcelona, Spain] for Gram-positive strains. When it was possible to determine a MIC, four concentrations of compound were tested, i.e., 2 × MIC, MIC, ½ MIC and ¼ MIC. A control with appropriate concentration of DMSO, as well as a negative control (TSB alone), was included. Sterile 96-well flat-bottomed untreated polystyrene microtiter plates were used. After a 24 h incubation at 37 °C, the biofilms were heat-fixed for 1 h at 60 °C and stained with 0.5% (*v*/*v*) crystal violet (Química Clínica Aplicada, Amposta, Spain) for 5 min. The stain was solubilized with 33% (*v*/*v*) acetic acid (Acetic acid 100%, AppliChem, Darmstadt, Germany) and the biofilm biomass was quantified by measuring the absorbance of each sample at 570 nm in a microplate reader (Thermo Scientific Multiskan^®^ EX, Thermo Fisher Scientific, Waltham, MA, USA) [20,34]. This assay was performed for reference strains.

#### 3.6.4. Antibiotic Synergy Testing

The potential synergy between the compounds and clinically relevant antimicrobial drugs was screened using the Kirby-Bauer method, as previously described [35]. A set of antibiotic discs (Oxoid, Basingstoke, UK) to which the isolates were resistant was selected: cefotaxime (CTX, 30 µg) for *E. coli* SA/2, vancomycin (VAN, 30 µg) for *E. faecalis* B3/101 and *E. faecium* 1/6/63, and oxacillin (OXA, 1 μg) for *S. aureus* 66/1. Antibiotic discs impregnated with 15 µg of each compound were placed on seeded MH plates. The controls used included antibiotic discs alone, blank paper discs impregnated with 15 µg of each compound alone and blank discs impregnated with DMSO. Plates with CTX were incubated for 18–20 h and plates with VAN and OXA were incubated for 24 h at 37 °C [30]. The potential synergy was considered when the inhibition halo of an antibiotic disc impregnated with the compound was greater than the inhibition halo of the antibiotic or compound-impregnated blank disc alone. The combined effect of the compounds and clinical relevant antimicrobial drugs was also evaluated by determining the antibiotic MIC in the presence of each compound. Briefly, when it was not possible to determine a MIC value for the test compound, the MIC of CTX (Duchefa Biochemie, Haarlem, The Netherlands), VAN (Oxoid, Basingstoke, UK), and OXA (Sigma-Aldrich, St. Louis, MO, USA) for the respective multidrug-resistant strain was determined in the presence of the highest concentration of each compound tested in previous assays. In the case of **1e**, the concentration used was 32 mg/L while it was 64 mg/L for the other compounds. The antibiotic tested was serially diluted whereas the concentration of each compound was kept fixed. Antibiotic MICs were determined as described above. For **7**, it was possible to determine the MIC for *E. faecalis* B3/101 and *E. faecium* 1/6/63, so the checkerboard method was used instead, as previously described [34]. The fractional inhibitory concentrations (FIC) were calculated as follows: FIC of compound = MIC of compound combined with antibiotic/MIC compound alone, and FIC antibiotic = MIC of antibiotic combined with a compound/MIC of antibiotic alone. The FIC index (FICI) was calculated as the sum of each FIC and interpreted as follows: FICI ≤ 0.5, ‘synergy’; 0.5 < FICI ≤ 4, ‘no interaction’; FICI > 4, ‘antagonism’ [36].

## 4. Conclusions

Marine-derived fungi have proved to be important sources of bioactive secondary metabolites, many of which exhibit cytotoxic and antibiotic activities. One of the most studied marine-derived fungi is of the genus *Penicillium*. In the past ten years, the *Penicillium* species from the marine environment received more attention than other fungal genera since compounds isolated from members of the *Penicillium* genus accounted for more than 25% of compounds of marine fungal origin. Although polyketides are the major secondary metabolites isolated from marine-derived *Penicillium* species, other structural classes of secondary metabolites such as alkaloids, terpenoids, and sterols are also isolated. In this work, we have described isolation and structure elucidation of two common fungal sterol derivatives: β-sitostenone and ergosterol 5,8-endoperoxide, fifteen polyketides, five of which have not been previously described, and a macrocyclic ether containing 1,4-disubstituted phenyl and succinamide moiety called GKK1032B, from the culture of the fungus *P. erubescens* strain KUFA 0220, which was isolated from the marine sponge *Neopetrosia* sp., collected from the Gulf of Thailand. From the compounds evaluated for their antibacterial activity against Gram-positive and Gram-negative bacteria of reference strains and multidrug-resistant isolates, their capacity to inhibit biofilm formation and synergistic effect, only GKK1032B displayed significant activities in all assays. Although the rest of the compounds, including those which have not been previously described, did not show significant antibacterial activity, it does not mean that they are void of bioactivities. Therefore, it is necessary to test these compounds in other bioassay platforms to explore their potential. Finally, it is worth mentioning that this is the first report of the chemical study of the marine-derived *P. erubescens*.

## Reference

## Figures and Tables

**Table 1:** The ^1^H and ^13^C NMR (DMSO-*d*_6_, 500.13 and 125.4 MHz) and HMBC assignment for **1c**.

Position	δ_C_, Type	δ_H_, (*J* in Hz)	HMBC
1	59.5, CH_2_	4.41, brs	C-2, 5
2	167.3, C	-	-
3	104.1, CH	6.25, s	C-1, 2, 5
4	174.8, CO	-	-
5	111.2, C	-	-
6	62.6, CH_2_	5.02, s	C-4, 5, 7, 9
7	155.2, C	-	-
8	105.9, C	-	-
9	152.2, C		-
10	106.5, CH	6.44, s	C-7, 8, 9, 11, 12
11	151.9, C	-	-
12	143.6, C	-	-
13	110.7, CH	7.15, s	C-7, 8, 9, 11, 12
OCH_3_-12	56.4, CH_3_	3.80	C-12

**Table 2:** The ^1^H and ^13^C NMR of **3a** (DMSO-*d*_6_, 300.13 and 75.4 MHz) and **3b** (DMSO, 500.13 and 125.4 MHz).

3a			3b	
Position	δ_C_, Type	δ_H_, (*J* in Hz)	δ_C_, Type	δ_H_, (*J* in Hz)
1	28.4, CH_3_	1.45, s	22.4, CH_3_	1.44, s
2	94.2, C	-	97.7, C	-
3	37.5, CH_2_	2.55, d (17.5)	37.1, CH_2_	2.63, dd (17.6, 2.6)
		2.87, d (17.5)		2.96, dd (17.6, 2.6)
4	158.7, C	-	157.9, C	-
5	113.5, C	-	113.0, C	-
6	56.3, CH_2_	4.45, s	52.7, CH_2_	4.22, dt (14.9, 0.9)
		-		4.52, dd (14.9, 2.1)
7	173.4, CO	-	173.2, CO	-
8	115.4, C	-	115.4, C	-
9	152.1, C	-	152.1, C	-
10	102.7, CH	6.83, s	102.7, CH	6.83, s
11	150.8, C	-	150.8, C	-
12	144.3, C	-	144.4, C	-
13	107.4, CH	7.26, s	107.4, CH	7.26, s
OCH_3_	-	-	48.3, CH_3_	3.21, s

**Table 3:** The ^1^H and ^13^C NMR (DMSO-*d*_6_, 300.13 and 75.4 MHz) and HMBC assignment for **4**.

Position	Δ_c_, Type	δ_H_, (*J* in Hz)	COSY	HMBC
2a	69.8, CH_2_	4.49, d (12.4)	2b	C-4, 4′, 8a
2b		4.63, d (12.4)	2a	C-3, 4, 4′, 8a-
3	86.3, C	-	-	-
4	181.6, CO	-	-	-
4a	111.1, C	-	-	-
5	110.3, CH	7.05, s	-	C-4, 6, 7, 8a
6	141.9, C	-	-	-
7	155.9, C	-	-	-
8	103.2, CH	6.41, s	-	C-4, 4a, 6, 7, 8a
8a	156.9, C	-	-	-
2′	191.4, C	-	-	-
3′	103.8, CH	5.68, d (0.8)	5′	C-2′, 3, 4′
4′	198.3, CO	-	-	-
5′	16.4, CH_3_	2.31, s	3′	C-2′, 3′
OH	-	10.01, brs	-	-

**Table 4:** The ^1^H and ^13^C NMR (DMSO-*d*_6_, 500.13 and 125.4 MHz) and HMBC assignment for **5**.

Position	δ_C_, Type	δ_H_, (*J* in Hz)	HMBC
2	158.9, CH	8.73, s	C-3, 4, 8a, 10
3	117.4, C	-	-
4	173.4, CO	-	-
4a	112.0, C	-	-
5	*	-	-
6	143.2, C	-	-
7	157.1	-	-
8	104.0, CH	7.03, s	-
8a	152.8, C	-	-
9	167.1, CO	-	-
10	134.9, CH	7.35, s	2, 4, 12
11	128.7, CH	7.35, s	3
12	198.2, CO	-	-
13	17.5, CH_3_	2.29, s	11, 12
OCH_3_-6	61.0, CH_3_	3.75, s	6

* not observed.

**Table 5:** The ^1^H and ^13^C NMR (DMSO-*d*_6_, 500.13 and 125.4 MHz) and HMBC assignment for **6**.

Position	δ_C_, Type	δ_H_, (*J* in Hz)	COSY	HMBC
2	161.3, C	-	-	-
3	112.3, C	-	-	-
4	172.2, CO	-	-	-
4a	115.1, C	-	-	-
5	108.0, CH	7.26, s	-	C-4, 6, 7, 8a
6	144.7, C	-	-	-
7	150.2, C	-	-	-
8	102.8, CH	6.84, s	-	C-4, 4a, 6, 7, 8a
8a	152.5, C		-	-
9α	33.4 CH_2_	3.47, d (19.2)	H-9β	C-2, 3, 10, 13, 15
9β		2.98, d (19.2)	H-9α	C-2, 3, 10, 13, 15
10	78.2, C	-	-	-
12	71.4, CH	5.41, s	-	C-2, 3, 3′,4, 4′, 10, 14
13	29.3, CH_3_	1.51, s	-	C-2, 9, 10, 14, 15
14	200.9, CO	-	-	-
15	69.8, CH	5.23, s	H-17	C-9, 10, 13, 14, 16
16	204.6, CO	-	-	-
17	32.7, CH_3_	2.16, s	H-15	C-15, 16
2′α	67.7, CH_2_	4.36, d (12.8)	H-2′β	C-3′, 4, 8′a, 12, 14
2′β		3.59, d (12.8)	H-2′α, 15	C-3′, 4, 8′a, 12, 14
3′	61.9, C	-	-	-
4′	185.3, CO	-	-	-
4′a	109.8, C	-	-	-
5′	111.1, CH	7.17, s	-	C-4′, 6′, 7′, 8′a
6′	141.1, C	-	-	-
7′	155.4, C	-	-	-
8′	102.6, CH	6.37, s	-	C-4′, 4′a, 6′, 8′a
8′a	156.0, C	-	-	-

**Table 6:** The ^1^H and ^13^C NMR (DMSO-*d*_6_, 500.13 and 125.4 MHz) and HMBC assignment for **7**.

Position	δ_C_, Type	δ_H_, (*J* in Hz)	HMBC
1	125.7, CH	8.00, s	C-3, 4a, 9′
2	129.5, C	-	-
3	138.0, C	-	-
4	132.2, C	-	-
4a	152.1, C	-	-
5	103.1, CH	6.93, s	C-7, 8a, 10a
6	150.9, C	-	-
7	144.3, C	-	-
8	108.7, C	7.45, s	C-6, 7, 9, 10a
8a	113.5, C	-	-
9	173.5, CO	-	-
9a	118.6, C	-	-
10a	154.2, C	-	-
11	16.6, CH_3_	2.32, s	C-2, 3, 4
12	202.8, CO	-	-
13	32.4, CH_3_	2.71, s	C-12
2′	66.2, CH_2_	4.67, s	C-3′, 4′, 8′a, 9′
3′	103.9, C	-	-
4′	183.6, CO	-	-
4′a	111.9, C	-	-
5′	110.5, CH	7.19, s	C-4′, 6′, 7′, 8′a
6′	155.9, C	-	-
7′	141.6, C	-	-
8′	103.3, CH	6.34, s	C-4′a, 6′, 7, 8′a
8′a	154.9, C	-	-
9′	172.6, C	-	-

**Table 7:** The antibacterial activity of **8** against a Gram-positive reference and multidrug-resistant strains. MIC and MBC are expressed in mg/mL.

Strains	*E. faecalis* ATCC29212	*E. faecium* ATCC19434	*S. aureus* ATCC29213	*E. faecalis* B3/101 (VRE)	*E. faecium* 1/6/63 (VRE)	*S. aureus* 66/1 (MRSA)
Disc diffusion	+	+	+	+	+	+
MIC	8	16	32	8	32	>64
MBC	>64	>64	64	>64	>64	>64

MIC, minimal inhibitory concentration; MBC, minimal bactericidal concentration; VRE, vancomycin-resistant *Enterococcus*; MRSA, methicillin-resistant *Staphylococcus aureus*; (-), no inhibition halo; (+), 7–9 mm inhibition halo.

**Table 8:** The classification of the ability of *E. faecalis* ATCC 29212 to adhere to and form biofilm after exposure to **1a**–**e**, **2a**, **3a**, **4**, **7**–**9**, in comparison to the untreated control.

Compound	Concentration (mg/L)	OD ± SD	Classification
**1a**	64	1.205 ± 0.025	strong
**1b**	64	1.547 ± 0.218	strong
**1c**	64	1.673 ± 0.308	strong
**1d**	64	1.522 ± 0.308	strong
**1e**	32	1.378 ± 0.378	strong
**2a**	64	1.136 ± 0.138	strong
**3a**	64	2.128 ± 0.248	strong
**4**	64	0.867 ± 0.280	strong
**7**	64	1.192 ± 0.239	strong
**8**	16 (2 × MIC)	0.089 ± 0.002	weak
**8**	8 (MIC)	0.099 ± 0.006	weak
**8**	4 (1/2 MIC)	1.884 ± 0.220	strong
**8**	2 (1/4MIC)	2.358 ± 0.416	strong
**9**	64	0.263 ± 0.014	moderate
None	0	0.080 ± 0.002	strong

OD = optical density; SD = standard deviation; The classification used is based on criteria in [19], Average OD value for negative control was found to be 0.055 ± 0.002, therefore the optical cut-off value (ODc) is equal to 0.055 + (3 × 0.002) = 0.061; 2 × ODc = 0.122; 4 × ODc = 0.244.

**Table 9:** The combined effect of clinically used antibiotics with **1a**–**e**, **2a**, **3a**, **4**, **7**–**9** against multidrug-resistant strains. MICs are expressed in mg/mL.

Compound	*E. coli* SA/2		*E. faecalis* B3/101		*E. faecium* 1/6/63		*S. aureus* 66/1	
	CTX		VAN		VAN		OXA	
	Disc Diffusion	MIC	Disc Diffusion	MIC	Disc Diffusion	MIC	Disc Diffusion	MIC
Antibiotic	+	512	-	1024	-	1024	-	64
Antibiotic + **1a**	-	512	-	1024	-	1024	-	64
Antibiotic + **1b**	+	512	-	1024	-	1024	-	64
Antibiotic + **1c**	-	>512	-	1024	-	1024	-	64
Antibiotic + **1d**	-	512	-	512	-	1024	-	64
Antibiotic + **1e**	-	512	-	1024	-	>1024	-	64
Antibiotic + **2a**	-	512	-	1024	-	1024	-	64
Antibiotic + **3a**	-	512	-	512	-	1024	-	64
Antibiotic + **4**	-	512	-	1024	-	1024	-	64
Antibiotic + **7**	-	>512	-	1024	-	1024	-	64
Antibiotic + **8**	-	512	+	*	-	*	-	64
Antibiotic + **9**	-	512	-	512	-	512	-	64

MIC = minimal inhibitory concentration; (-) = no inhibition halo or no increase in the inhibition halo; (+) = halo of inhibition or increase of the inhibition halo by 2 mm; CTX = cefotaxime; VAN = vancomycin; OXA = oxacillin. * For this compound, the checkerboard assay was performed and, with FICI = 0.7 for *E. faecalis* B3/101 and FICI = 2 for *E. faecium* 1/6/63, no interaction between **8** and VAN was found (0.5 < FICI ≤ 4, ‘no interaction’).

## Supplementary Materials

The following are available online at http://www.mdpi.com/1660-3397/16/8/289/s1, Figure S1: Structures of β-sitosteanone and ergosterol-5,8-endoperoxide, isolated from the marine sponge-associated fungus Penicillium reubescens KUFA0220, Figures S2–S50 and S52–S53: 1D and 2D NMR spectra of isolated compounds, Figure S51: Ortep view of GKK1032B (**8**).

## References

[1] Visagle C.M., Houbraken J., Frisvad J.C., Hong S.B., Klaassen C.H.W., Perrone G., Seifert K.A., Vatga J., Yaguchi T., Samson R.A. (2014). Identification and nomenclature of the genus Penicillium. Sud. Mycol..

[2] Ma H.-G., Liu Q., Zhu G.-L., Liu H.-S., Zhu W.-M. (2016). Marine Natural Products sources from marine-derived *Penicillium* fungi. J. Asian Nat. Prod. Res..

[3] Kumla D., Aung T.S., Buttachon S., Dethoup T., Gales L., Pereira J.A., Inácio A., Costa P.M., Lee M., Sekeroglu N. (2017). A New Dihydrochromone Dimer and Other Secondary Metabolites from Cultures of the Marine Sponge-Associated Fungi Neosartorya fennelliae KUFA 0811 and Neosartorya tsunodae KUFC 9213. Mar. Drugs.

[4] May Zin W.W., Buttachon S., Dethoup T., Pereira J.A., Gales L., Inácio A., Costa P.M., Lee M., Sekeroglu N., Silva A.M.S. (2017). Antibacterial and antibiofilm activities of the metabolites isolated from the culture of the mangrove-derived endophytic fungus Eurotium chevalieri KUFA0006. Phytochemistry.

[5] Capon R.J., Stewart M., Ratnayake R., Lacey E., Gill J.H. (2007). Citromycetins and Bilains A–C: Newaromatic polyketides and diketopiperazines from Australian Marine-Derived and Terrestrial Penicillium spp.. J. Nat. Prod..

[6] Yuan C., Wang H.-Y., Wu C.-S., Jiao Y., Li M., Wang Y.-Y., Wang S.-Q., Zhao Z.-T., Lou H.-X. (2013). Austdiol, fulvic acid and citromycetin derivative from an endolichenic fungus, Myxotrichum sp.. Phytochem. Lett..

[7] Fujita K.-I., Nagamine Y., Ping X., Taniguchi M. (1999). Mode of Action of anhydrofulvic acid against Candida utilis ATCC 42402 under acid condition. J. Antibiot..

[8] Lee D.-S., Jang J.-H., Ko W., Kim K.-S., Sohn J.H., Kang M.-S., Ahn J.S., Kim Y.-C., On H. (2013). PTP1B inhibitory and anti-inflamatory effects of secondary metabolites isolated from the marine-derived fungus Penicillium sp. JF-55. Mar. Drugs.

[9] Cheng X., Yu L., Wang Q., Ding W., Chen Z., Ma Z. (2018). New brefeldins and penialidins from marine fungus Penicillium janthinellum DT-F29. Nat. Prod. Res..

[10] Jouda J.B., Kusari S., Lamshoft M., Moufo Taontsi F., Douala Meli C., Wandji J., Spitteller M. (2014). Penialidins A–C with strong bacterial acivity from Penicillium sp., an endophytic fungus harboring leaves of Garcinia nobilis. Fitoterapia.

[11] Kimura T., Kikuchi K., Kumagai K., Hosotani N., Kishino A. Nerve Regeneration Promoters Containing Semaphorin Inhibitor as the Active Ingredient. European Patent EP 1 306 093 B1. Date of Publication and Mention of the Grant of the Patent 03.10. 2007. Bulletin 2007/40. https://data.epo.org/publication-server/rest/v1.0/publication-dates/20071003/patents/EP1306093NWB1/document.html.

[12] Song T., Chen M., Chai W., Zhang Z., Lian X.-Y. (2018). New bioactive pyrrospirones C-I from a marine-derived fungus Penicillium sp. ZZ380. Tetrahedron.

[13] Rukachaisirikul V., Satpradit S., Klaiklay S., Phongpaichit S., Borwornwiriyapan K., Sakayaroj J. (2014). Polyketide anthraquinone, diphenyl ether, and xanthone derivatives from the soil fungus Penicillium sp. PSU-RSPG99. Tetrahedron.

[14] Pastre R., Marinho A.M.R., Rodrigues-Filho E., Souza A.Q.L., Pereira J.O. (2007). Diversity of polyketides produced by Penicillium species isolated from Melia azedarach and Murraya paniculata. Quim. Nova.

[15] Noinart J., Buttachon S., Dethoup T., Gales L., Pereira J.A., Urbatzka R., Freitas S., Lee M., Silva A.M.S., Pinto M.M.M. (2017). A new ergosterol analog, a new bis-anthraquinone and anti-obesity activity of anthraquinones from the marine sponge-associated fungus Talaromyces stipitatus KUFA 0207. Mar. Drugs.

[16] Arai K., Miyajima H., Mushiroda T., Yamamoto Y. (1989). Metabolites of Penicillium italicum Wehmer. Isolation and structures of new metabolites including naturally occurring 4-ylidene-acyltetronic acids, italicinic acid and italicic acid. Chem. Pharm. Bull..

[17] Lu K., Zhang Y., Li L., Wang X., Ding G. (2013). Chaetochromones A and B, two new polyketides from the fungus Chaetomium indicum (CBS.860.68). Molecules.

[18] Kumagai K., Hosotani N., Kikuchi K., Kimuran T., Saji I. (2003). Xanthofulvin, a novel semaphorin inhibitor produced by a strain of Penicillium. J. Antibiot..

[19] Stepanović S., Vuković D., Dakic I., Savić B., Švabic-Vlahović M. (2000). A modified -plate test for quantification of staphylococcal biofilm formation. J. Microbiol. Methods.

[20] Stepanović S., Vuković D., Hola V., Di Bonaventura G., Djukić S., Ćirković I., Ruzicka F. (2007). Quantification of biofilm in microtiter plates: Overview of testing conditions and practical recommendations for assessment of biofilm production by staphylococci. Apmis.

[21] Tacconelli E., Carrara E., Savoldi A., Harbarth S., Mendelson M., Monnet D.L., Pulcini C., Kahlmeter G., Kluytmans J., Carmeli Y. (2017). Discovery, research, and development of new antibiotics: The WHO priority list of antibiotic-resistant bacteria and tuberculosis. Lancet Infect. Dis..

[22] Murray M.G., Thompson W.F. (1980). Rapid isolation of high molecular weight plant DNA. Nucleic Acids Res..

[23] White T.J., Bruns T., Lee S., Taylor J., Innis M.A., Gelfand D.H., Sninsky J.J., White T.J. (1990). Amplification and direct sequencing of fungal ribosomal RNA genes for phylogenetics. PCR Protocols: A Guide to Methods and Applications.

[24] Sanger F., Nicklen S., Coulson A.R. (1977). DNA sequencing with chain-terminating inhibitors. Proc. Natl. Acad. Sci. USA.

[25] Austin A., Petersson G.A., Frisch M.J., Dobek F.J., Scalmani G., Throssel K. (2012). A density functional with Spherical atom dispersion terms. J. Chem. Theory Comput..

[26] Stephens P.J., Harada N. (2010). ECD Cotton effect approximated by the Gaussian curve and other methods. Chirality.

[27] Sheldrick G.M. (2008). A short story of SHELX. Acta Cryst..

[28] Simões R.R., Aires-de-Sousa M., Conceicao T., Antunes F., da Costa P.M., de Lencastre H. (2011). High prevalence of EMRSA-15 in Portuguese public buses: A worrisome finding. PLoS ONE.

[29] Bessa L.J., Barbosa-Vasconcelos A., Mendes A., Vaz-Pires P., Martins da Costa P. (2014). High prevalence of multidrug-resistant *Escherichia coli* and *Enterococcus* spp. in river water, upstream and downstream of a wastewater treatment plant. J. Water Health.

[30] Clinical and Laboratory Standards Institute (CLSI) (2017). Performance Standards for Antimicrobial Susceptibility Testing.

[31] Clinical and Laboratory Standards Institute (CLSI) (2012). Performance Standards for Antimicrobial Disk Susceptibility Tests.

[32] Clinical and Laboratory Standards Institute (CLSI) (2015). Methods for Dilution Antimicrobial Susceptibility Tests for Bacteria That Grow Aerobically.

[33] Clinical and Laboratory Standards Institute (CLSI) (1999). Methods for Determining Bactericidal Activity of Antimicrobial Agents.

[34] Gomes N.M., Bessa L.J., Buttachon S., Costa P.M., Buaruang J., Dethoup T., Silva A.M.S., Kijjoa A. (2014). Antibacterial and antibiofilm activities of tryptoquivalines and meroditerpenes isolated from the marine-derived fungi Neosartorya paulistensis, N. laciniosa, N. tsunodae, and the soil fungi N. fischeri and N. siamensis. Mar. Drugs.

[35] Buttachon S., Ramos A.A., Inácio Â., Dethoup T., Gales L., Lee M., Costa P.M., Silva A.M.S., Sekeroglu N., Rocha E. (2018). Bis-indolyl benzenoids, hydroxypyrrolidine derivatives and other constituents from cultures of the marine sponge-associated fungus Aspergillus candidus KUFA0062. Mar. Drugs.

[36] Odds F.C. (2003). Synergy, antagonism, and what the chequerboard puts between them. J. Antimicrob. Chemother..

