# Supplementary material for: Chromone Derivatives and Other Constituents from Cultures of the Marine Sponge-Associated Fungus Penicillium erubescens KUFA0220 and Their Antibacterial Activity
Source: Mar Drugs. 2018 Aug 20;16(8):289. doi: 10.3390/md16080289 (PMC6117697; doi:10.3390/md16080289)

Supplementary Materials

Chromone Derivatives and Other Constituents from Cultures of the Marine Sponge-Associated Fungus *Penicillium erubescens* KUFA0220 and Their Antibacterial Activity

Decha Kumla [1,2], José A. Pereira [1,2], Tida Dethoup [3], Luís Gales [1,4], Joana Freitas-Silva[1,2], Paulo M. Costa [1,2], Michael Lee [5], Artur M. S. Silva [6], Nazim Sekeroglu[7], Madalena M. M. Pinto [2,8]* and Anake Kijjoa [1,2,]*

[1] *ICBAS*-Instituto de Ciências Biomédicas Abel Salazar, Rua de Jorge Viterbo Ferreira, 228, 4050-313 Porto, Portugal. E-mail: Decha1987@hotmail.com (D.C.), jpereira@icbas.up.pt (J. A. P.); lgales@ibmc.up.pt (L.G.); pmcosta@icbas.up.pt (P.M.C.).

[2] Interdisciplinary Centre of Marine and Environmental Research (CIIMAR), Terminal de Cruzeiros do Porto de Lexões, Av. General Norton de Matos s/n, 4450-208 Matosinhos, Portugal. E-mail: joanafreitasdasilva@gmail.com.

[3] Department of Plant Pathology, Faculty of Agriculture, Kasetsart University, Bangkok 10240, Thailand. E-mail: tdethoup@yahoo.com.

[4] Instituto de Biologia Molecular e Celular (i3S-IBMC), Universidade do Porto, Rua de Jorge Viterbo Ferreira, 228, 4050-313 Porto, Portugal

[5] Department of Chemistry, University of Leicester, University Road, Leicester LE 7 RH, UK. E-mail: ml34@leicester.ac.uk.

[6] Departamento de Química & QOPNA, Universidade de Aveiro, 3810-193 Aveiro, Portugal. E-mail: artur.silva@ua.pt.

[7] Medicinal and Aromatic Plant Programme, Plant and Animal Sciences Department, Vocational School, Kilis 7 Aralık University, 79000, Kilis, Turkey. E-mail: nsekeroglu@gmail.com.

[8] Laboratório de Química Orgânica, Departamento de Ciências Químicas, Faculdade de Farmácia, Universidade do Porto, Rua de Jorge Viterbo Ferreira, 228, 4050-3 13 Porto, Portugal.

*Correspondence: ankijjoa@icbas.up.pt (A. K.); madalena@ff.up.pt (M. P.); Tel.: +351-22-042-8331 (A.K.) and +351-22-042-8331 (M. P.); Fax: +351-22-206-2232 (A.K.) and +351-22-206-2232 (M. P.)

Figure S1. Structures of β-sitosteanone and ergosterol 5,8-endoperoxide isolated from *Penicilium erubescens* KUFA 0220.

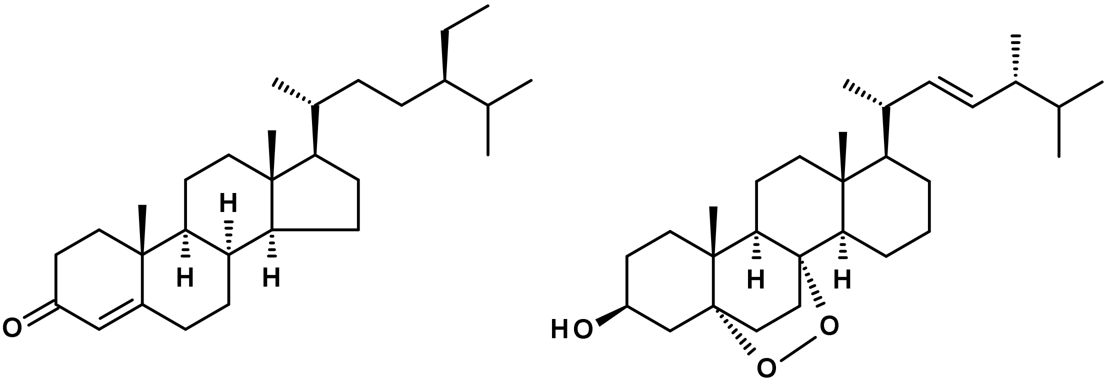

β-Sitostenone   Ergosterol-5,8-endoperoxide

Figure S2. ^{1}H NMR spectrum of β-sitostenone ($CDCl_3$, 300.13 MHz).

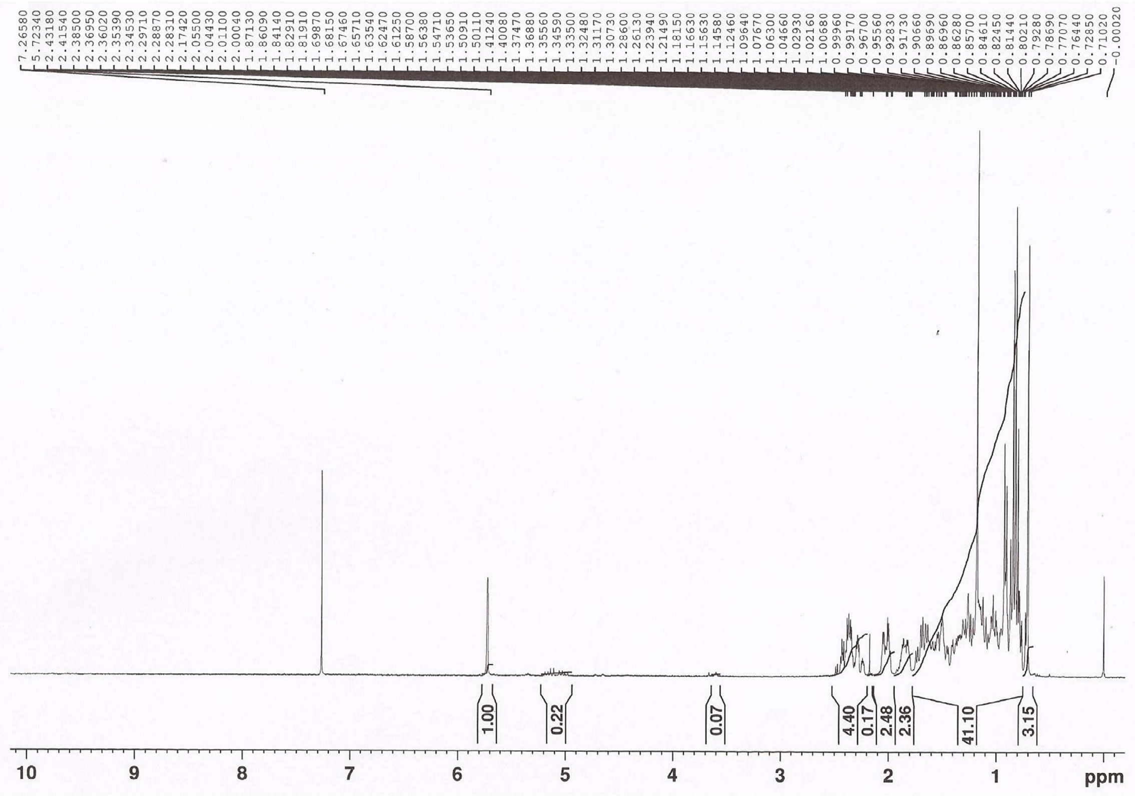

Figure S3. ^{13}C NMR spectrum of β-sitostenone ($CDCl_3$, 75.4 MHz).

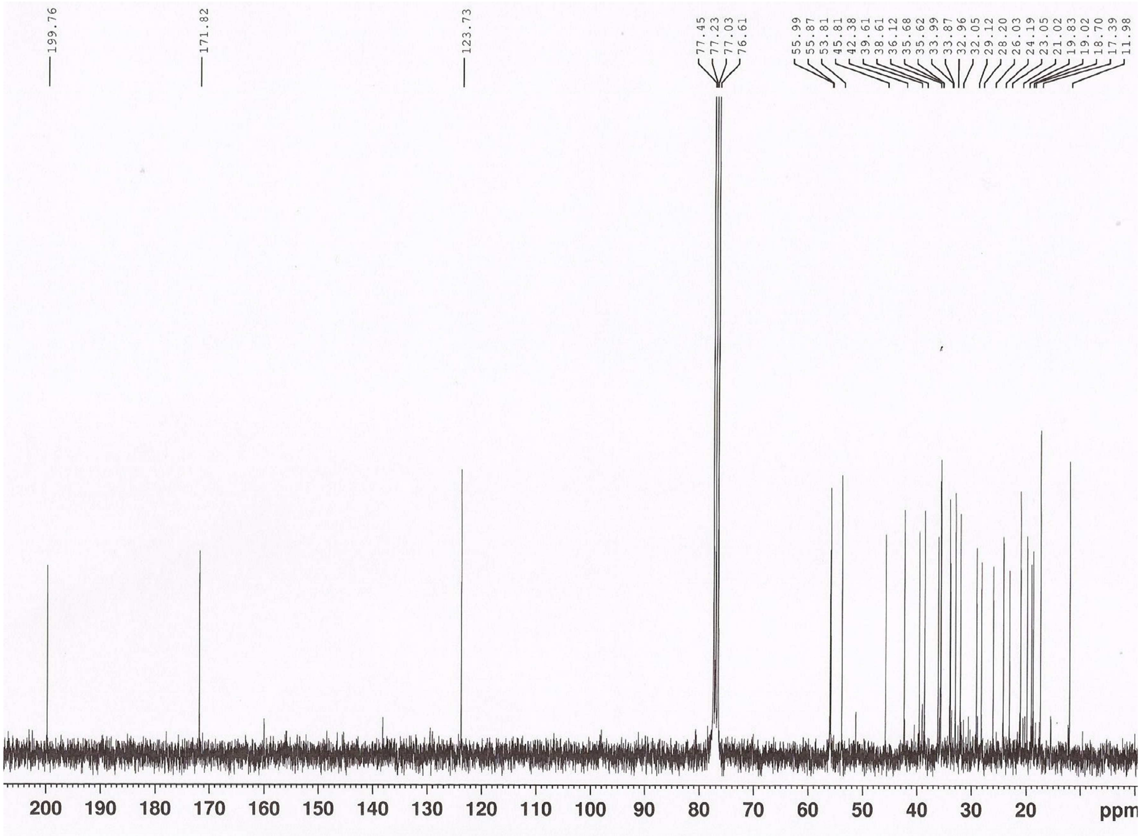

Figure S4. ^{1}H NMR spectrum of ergosterol-5, 8-endoperoxide ($CDCl_3$, 300.13 MHz).

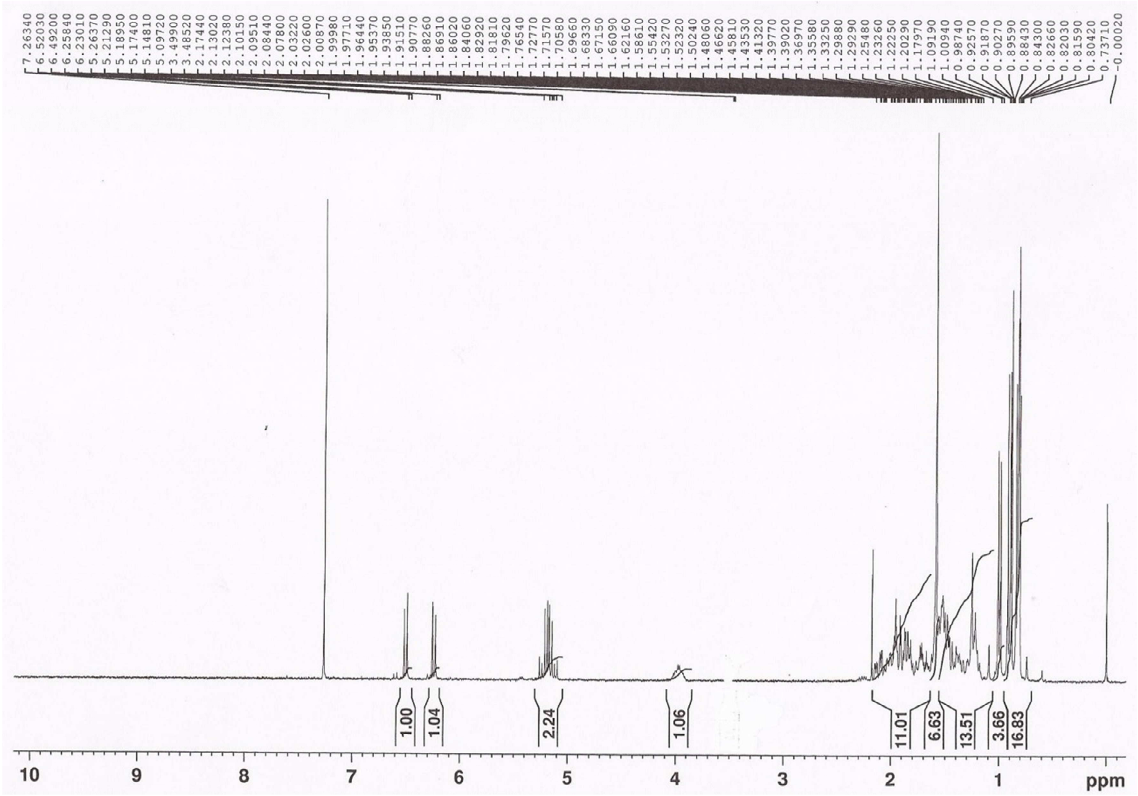

Figure S5. ^{13}C NMR spectrum of ergosterol-5, 8-endoperoxode ($CDCl_3$, 75.4 MHz).

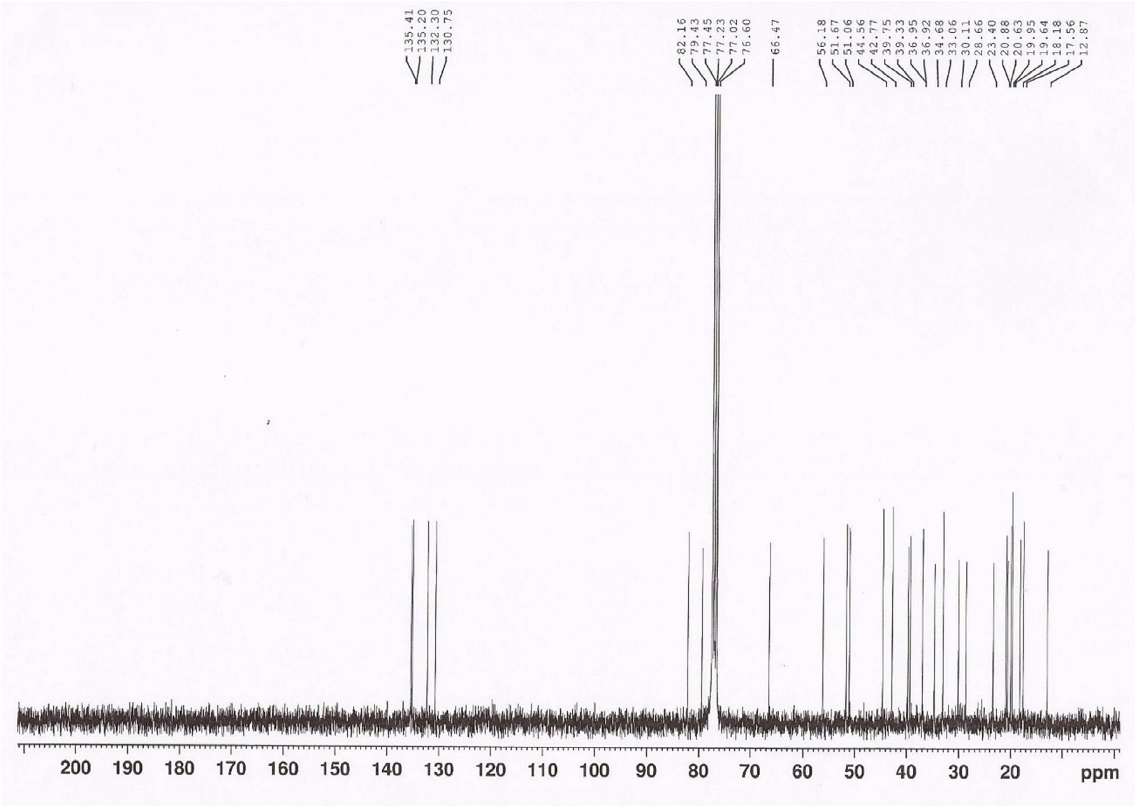

Figure S6. ^{1}H NMR spectrum of **1a** (DMSO-d_6, 300.13 MHz).

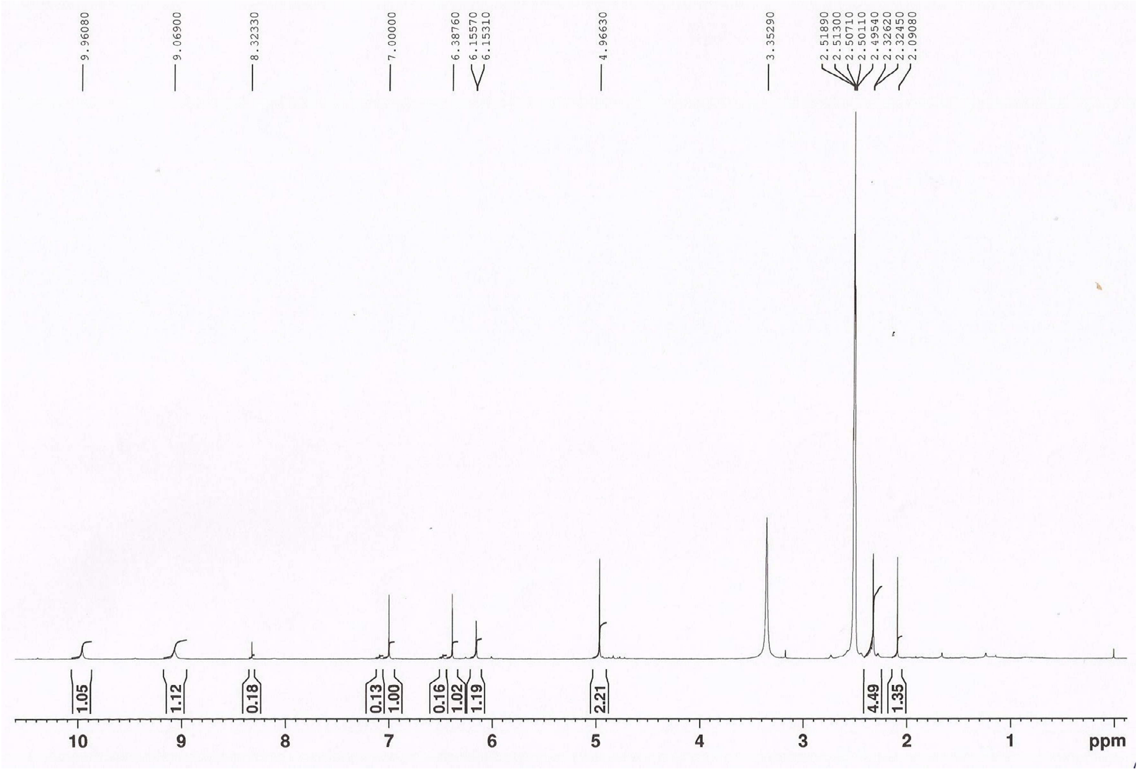

Figure S7. ^{13}C NMR spectrum of **1a** (DMSO-d$_6$, 75.4 MHz).

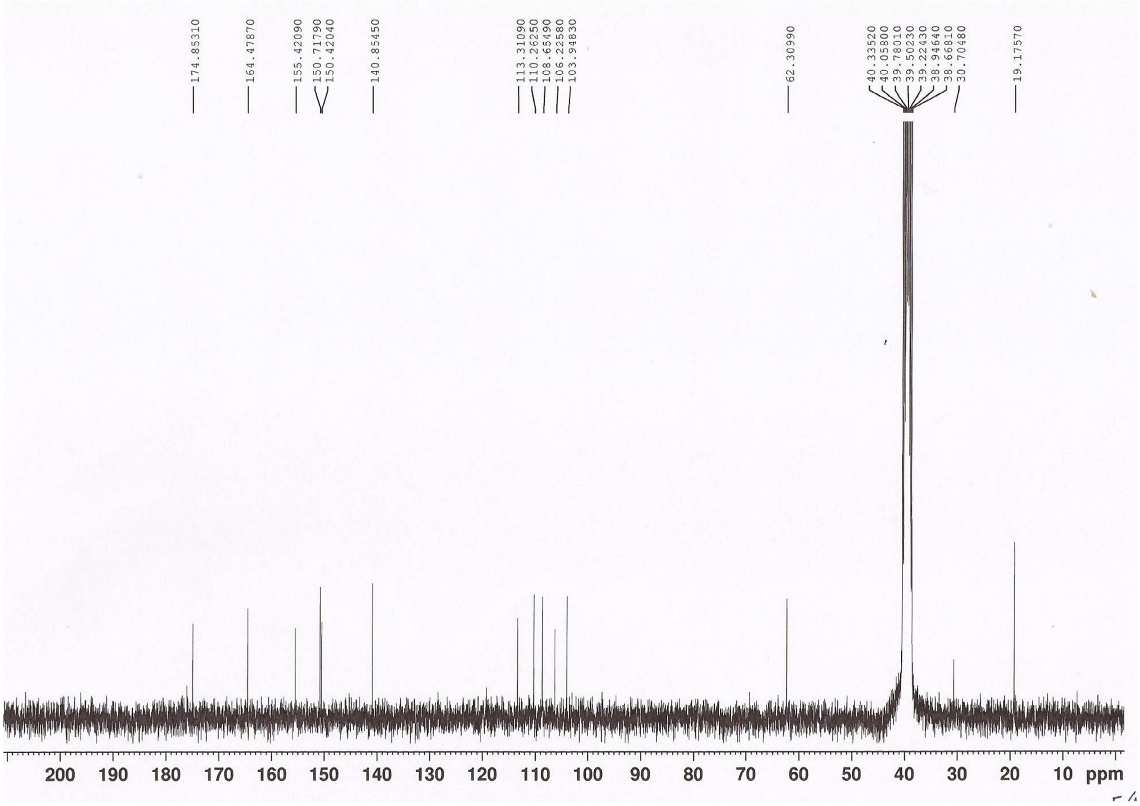

Figure S8. ^{1}H NMR spectrum of **1b** (DMSO-d$_6$, 300.13 MHz).

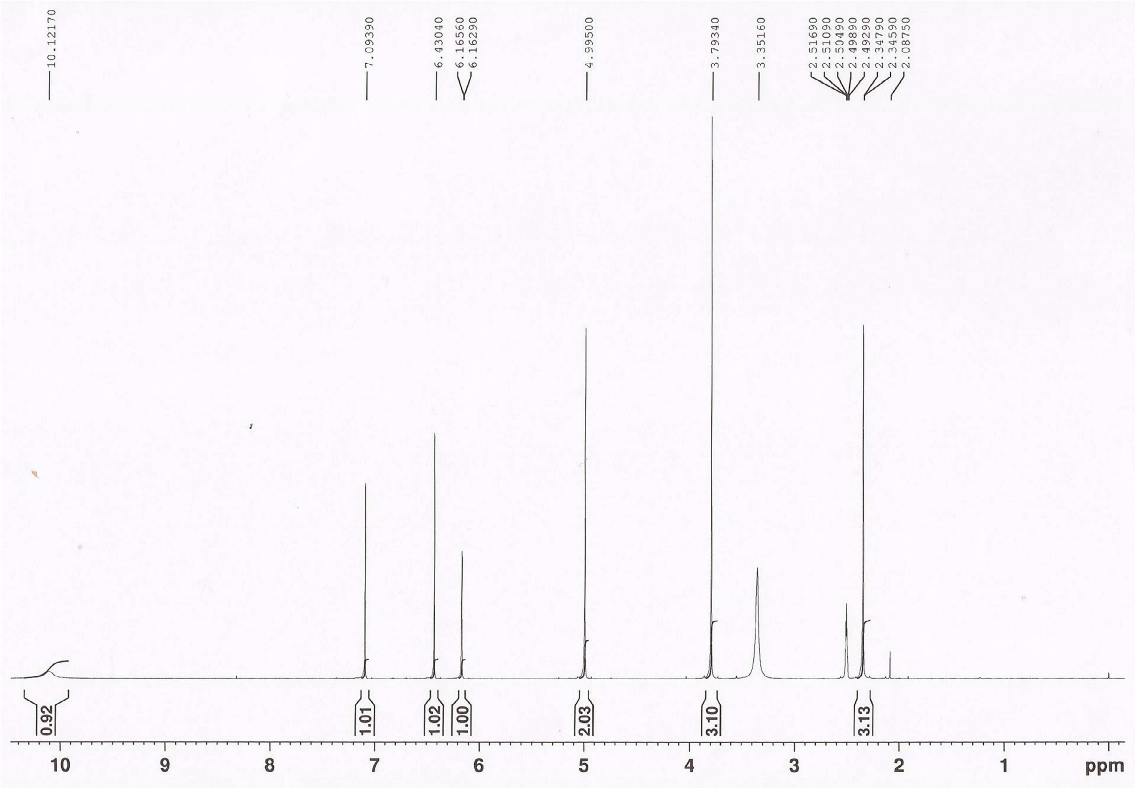

Figure S9. ^{13}C NMR spectrum of **1b** (DMSO-d$_6$, 75.4 MHz).

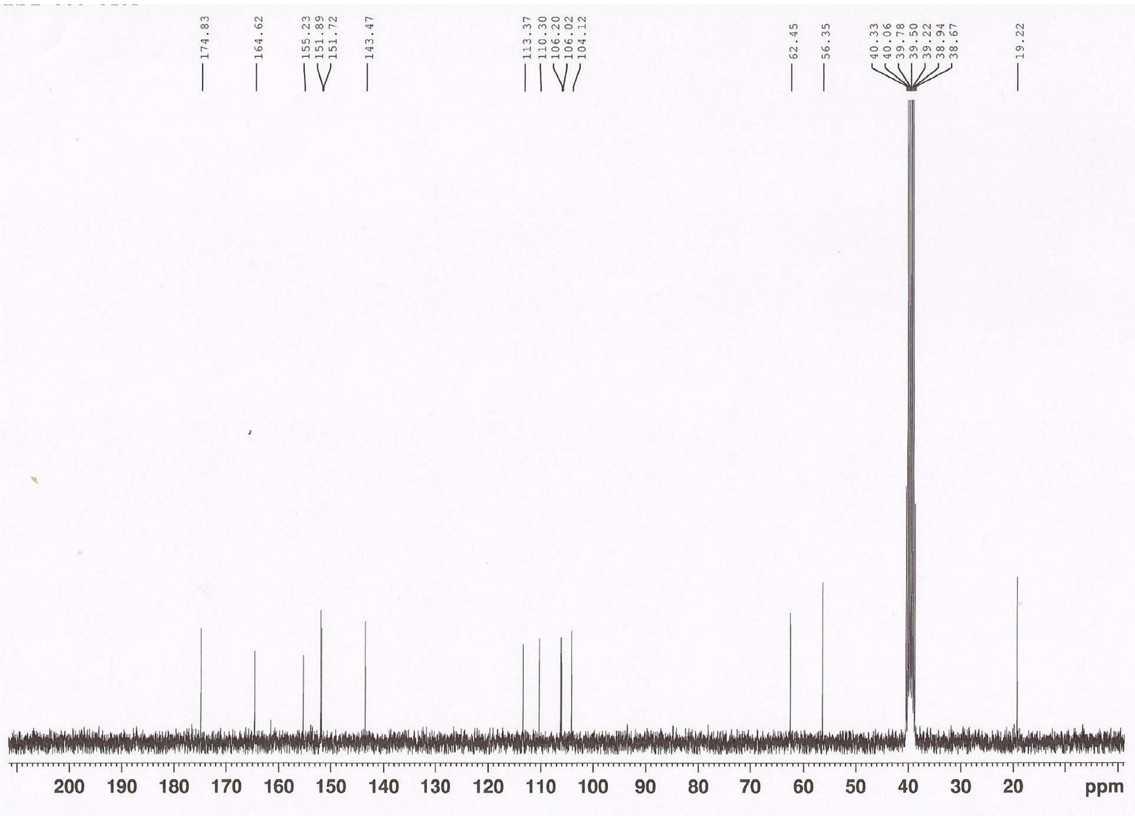

Figure S10. ^{1}H NMR spectrum of **1c** (DMSO-d$_6$, 500.13 MHz).

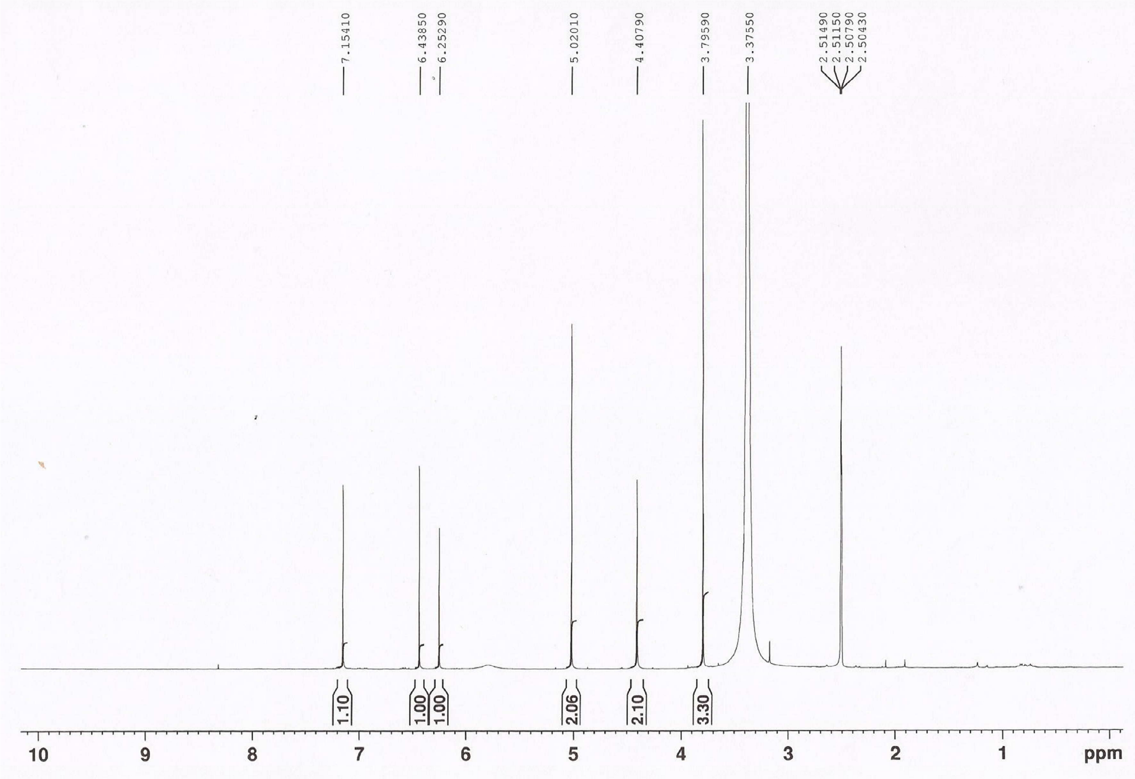

Figure S11. ^{13}C NMR spectrum of **1c** (DMSO-d$_6$, 125.4 MHz).

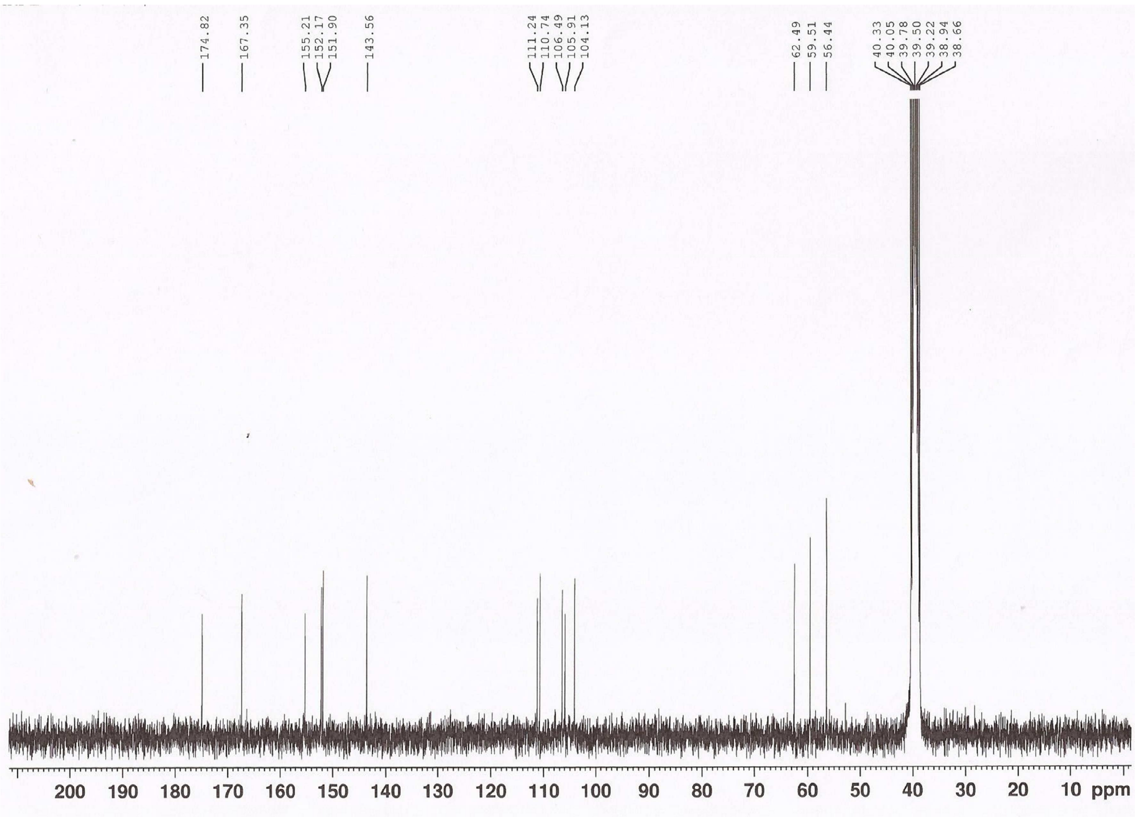

Figure S12. HSQC spectrum of **1c** (DMSO-d$_6$, 500.13 MHz).

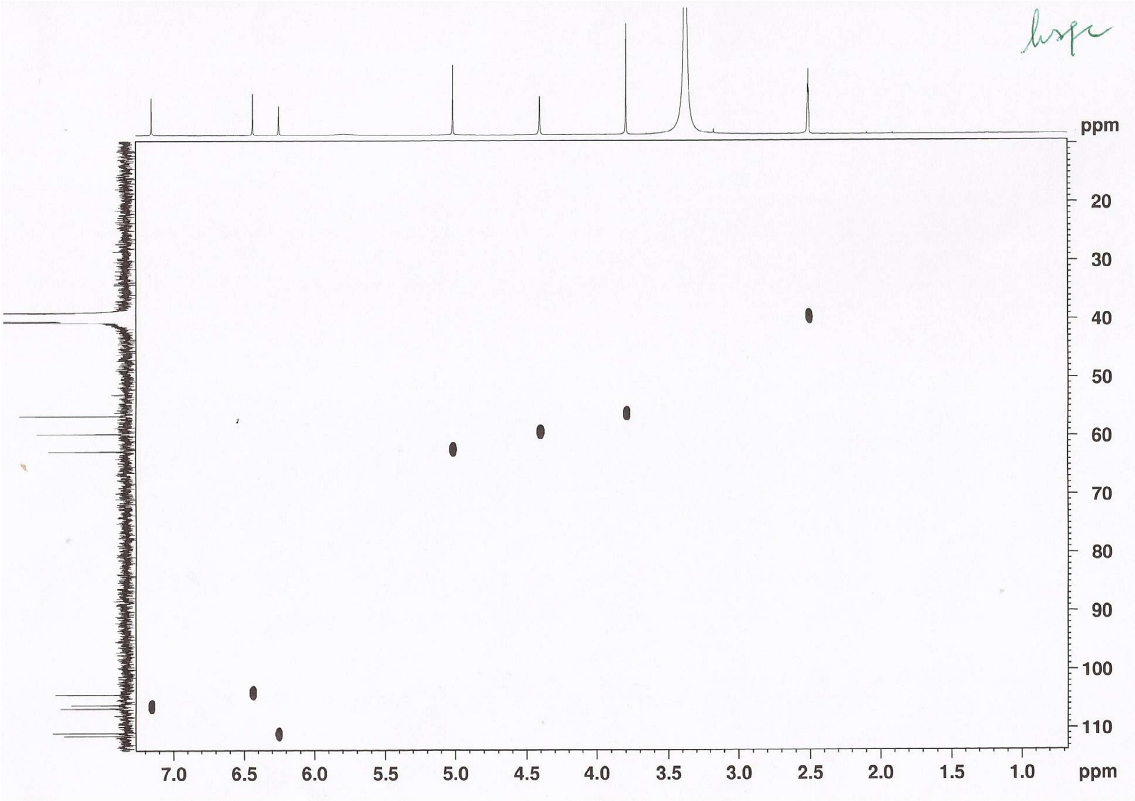

Figure S13. HMBC spectrum of **1c** (DMSO-d$_6$, 500.13 MHz).

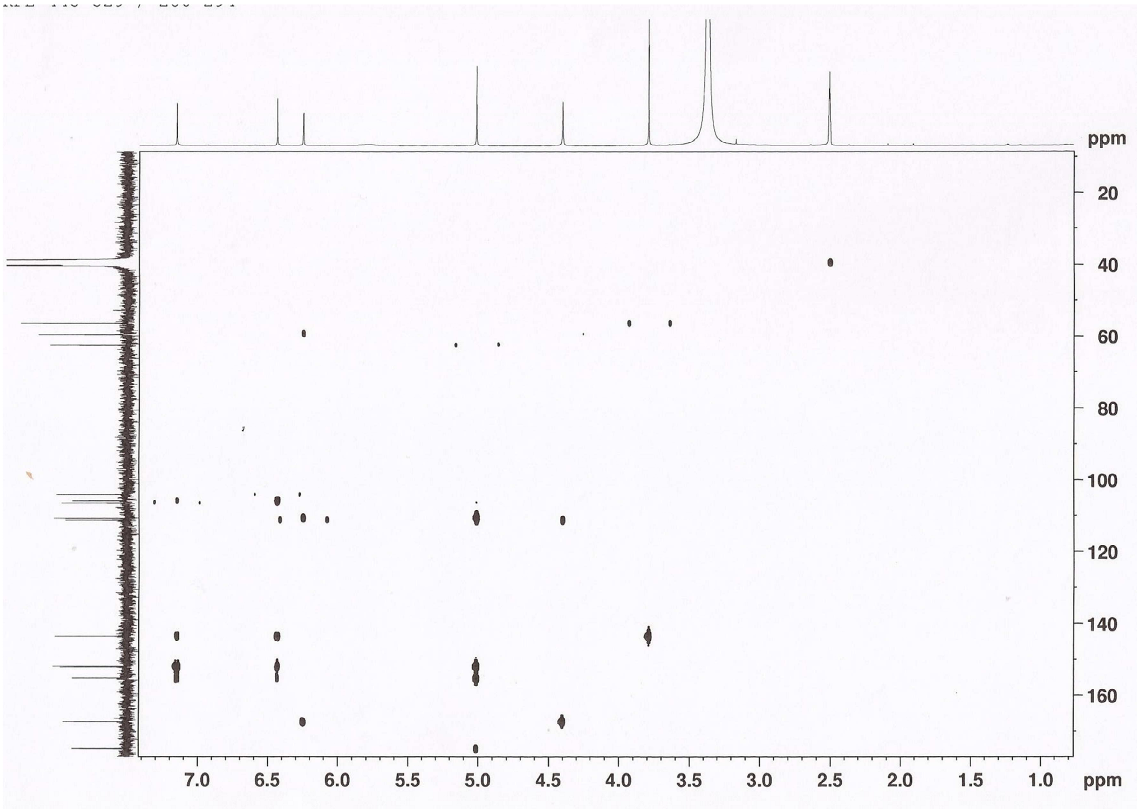

Figure S14. NOESY spectrum of **1c** (DMSO-d$_6$, 500.13 MHz).

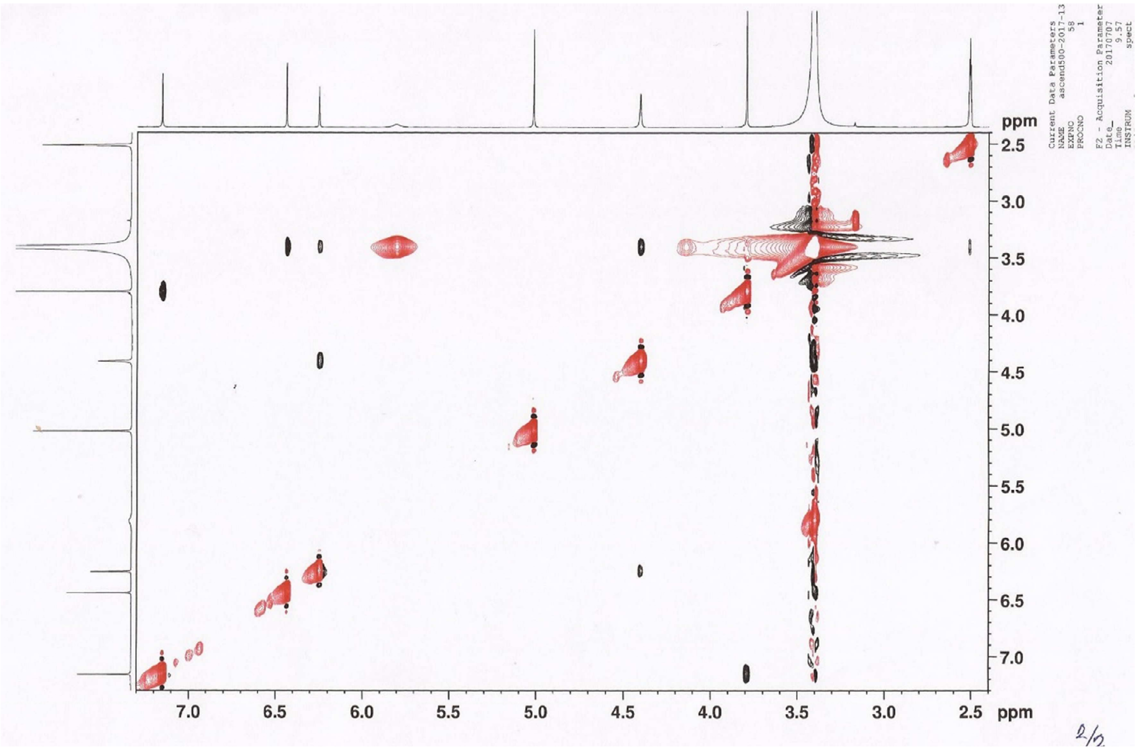

Figure S15. ^{1}H NMR spectrum of **1d** (DMSO-d$_6$, 500.13 MHz).

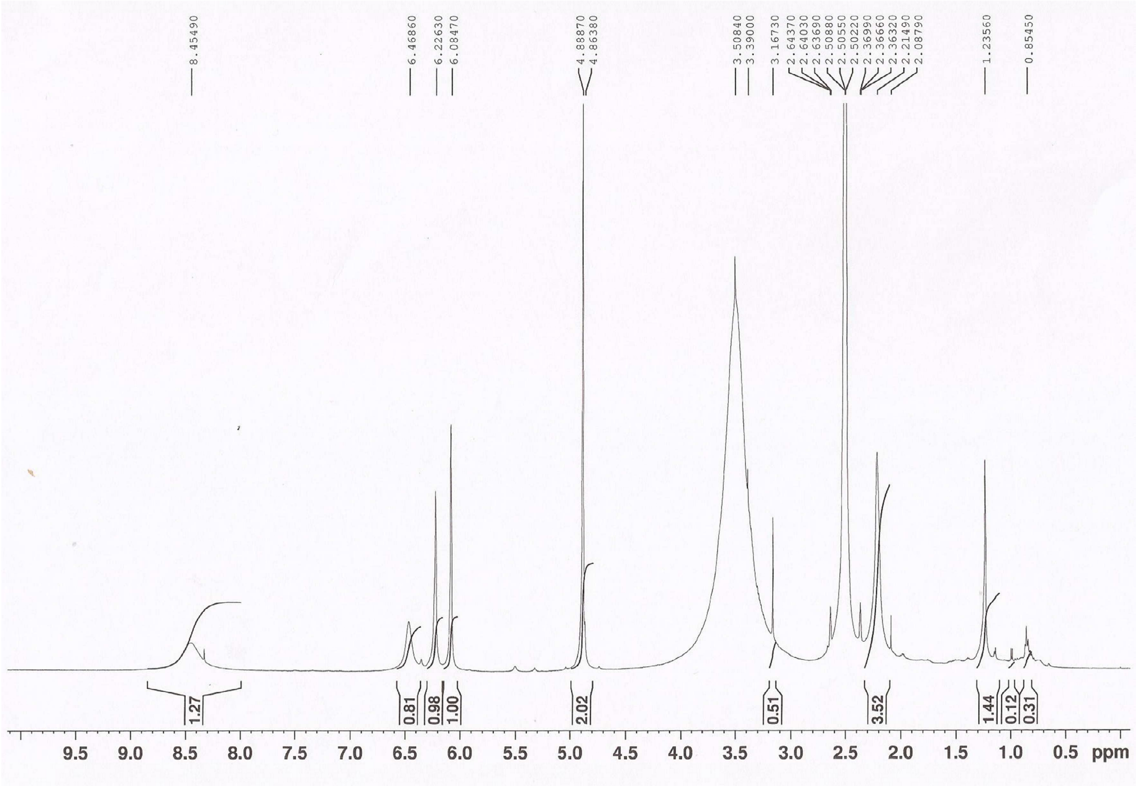

Figure S16. ^{13}C NMR spectrum of **1d** (DMSO-d$_6$, 125.4 MHz).

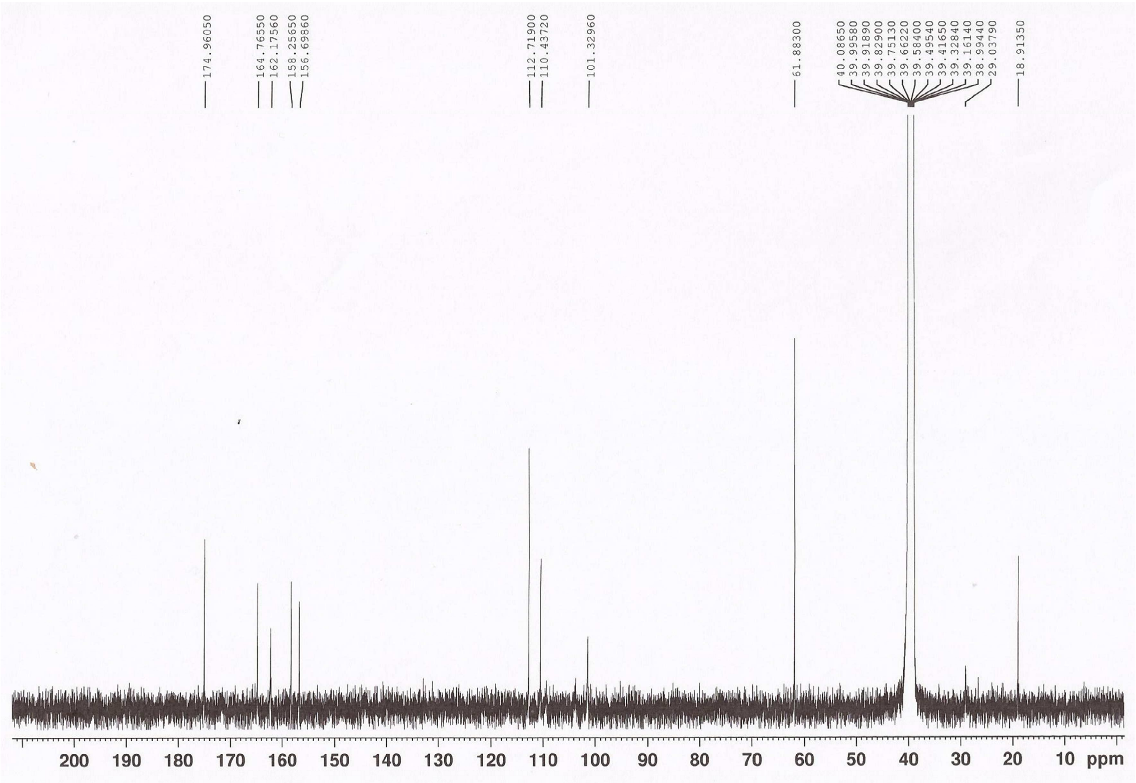

Figure S17. ^{1}H spectrum of **1e** (DMSO-d_6, 300.13 MHz).

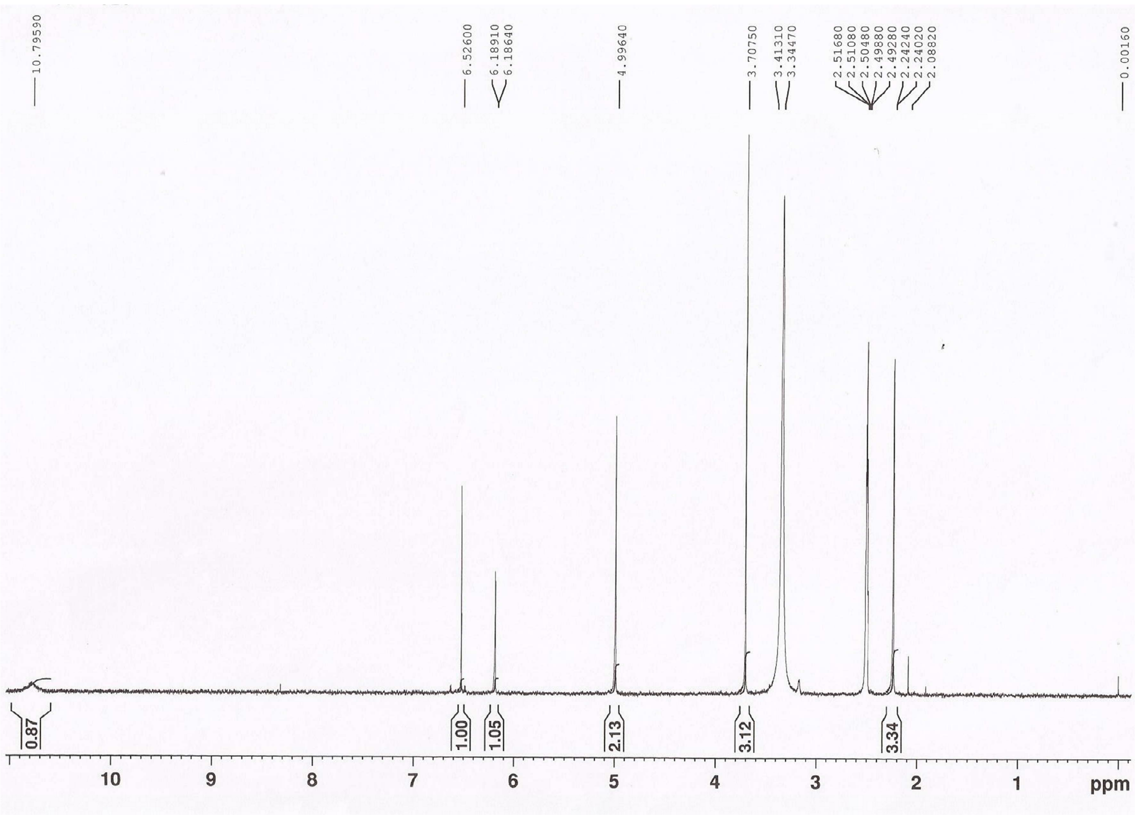

Figure S18. ^{13}C NMR spectrum of **1e** (DMSO-d_6, 75.4 MHz).

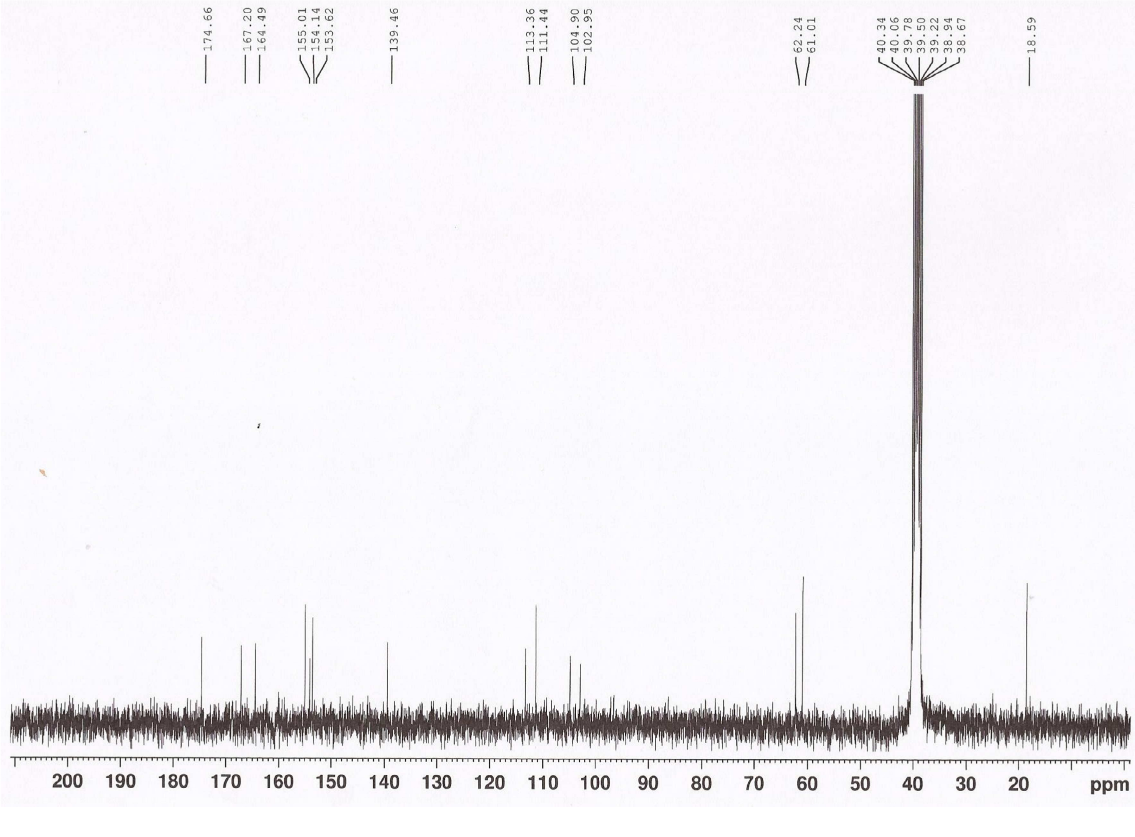

Figure S19. ^{1}H spectrum of **2a** (DMSO-d_6, 300.13 MHz).

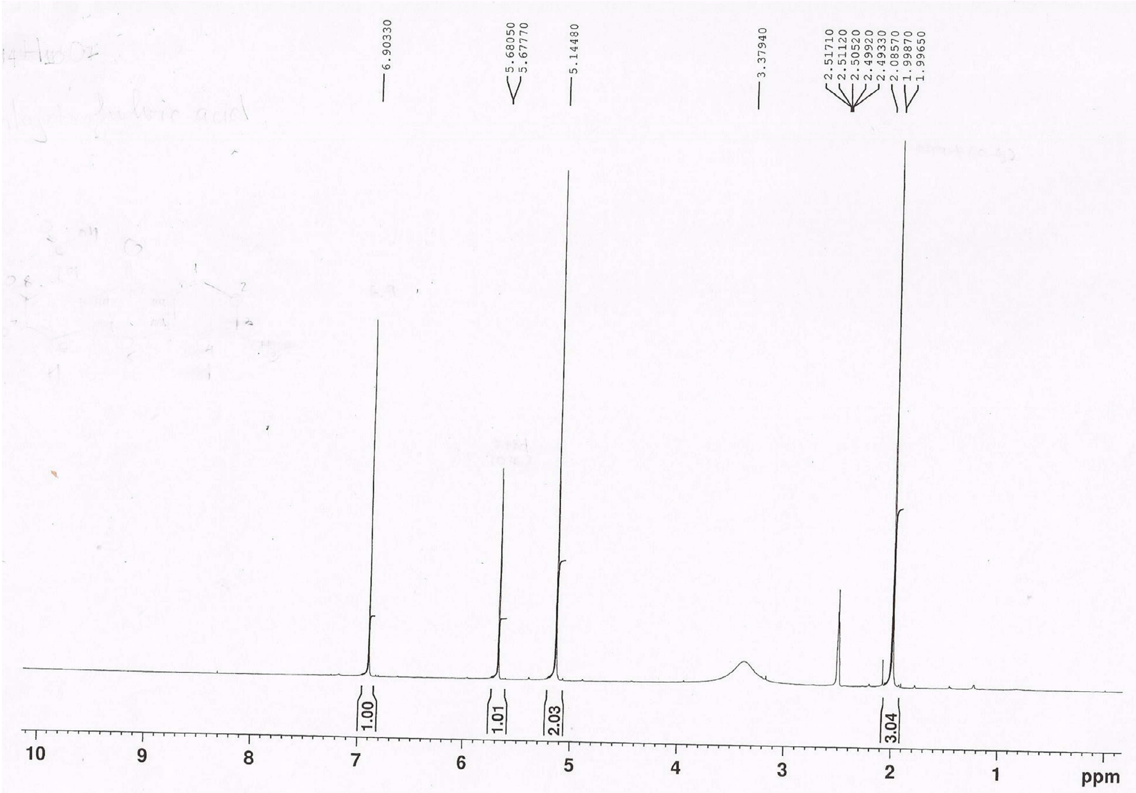

Figure S20. ^{13}C NMR spectrum of **2a** (DMSO-d_6, 75.4 MHz).

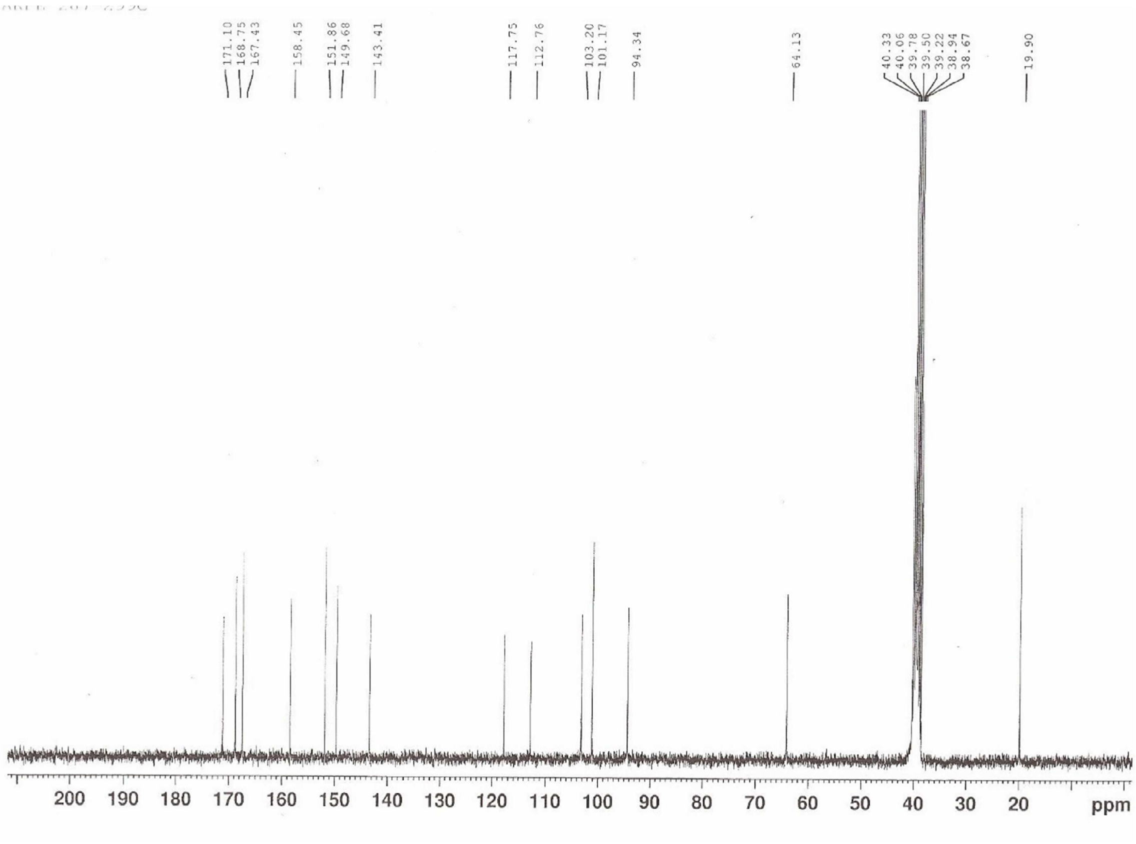

Figure S21. ^{1}H NMR spectrum of a mixture of **2b** and **3b** (DMSO-d_6, 500.13 MHz).

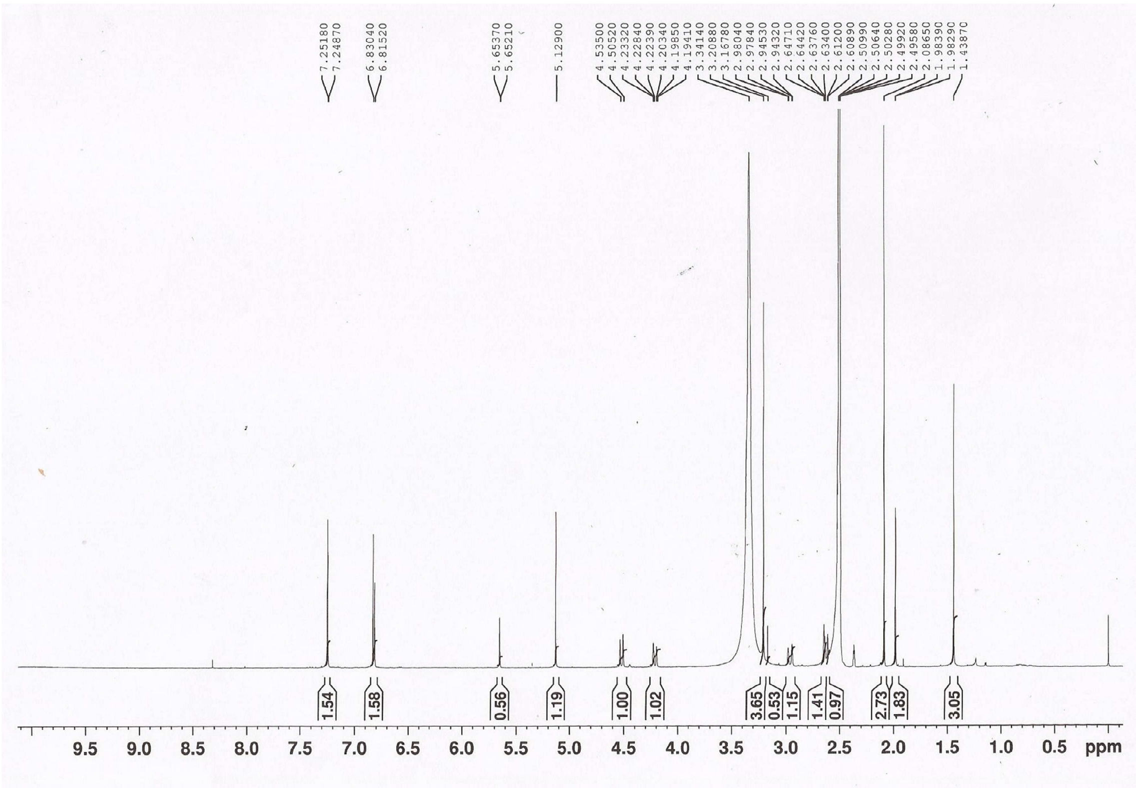

Figure S22. ^{13}C NMR spectrum of a mixture of **2b** and **3b** (DMSO-d_6, 125.4 MHz).

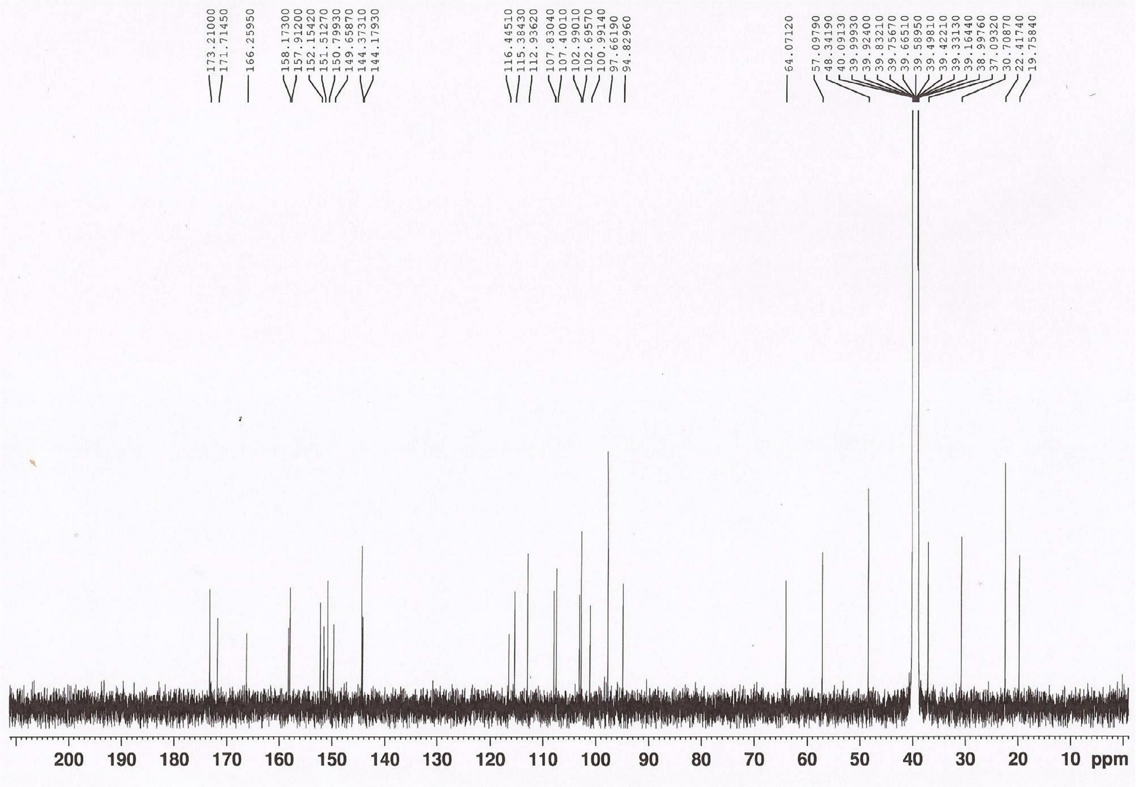

Figure S23. COSY spectrum of a mixture of **2b** and **3b** (DMSO-d$_6$, 500.13 MHz).

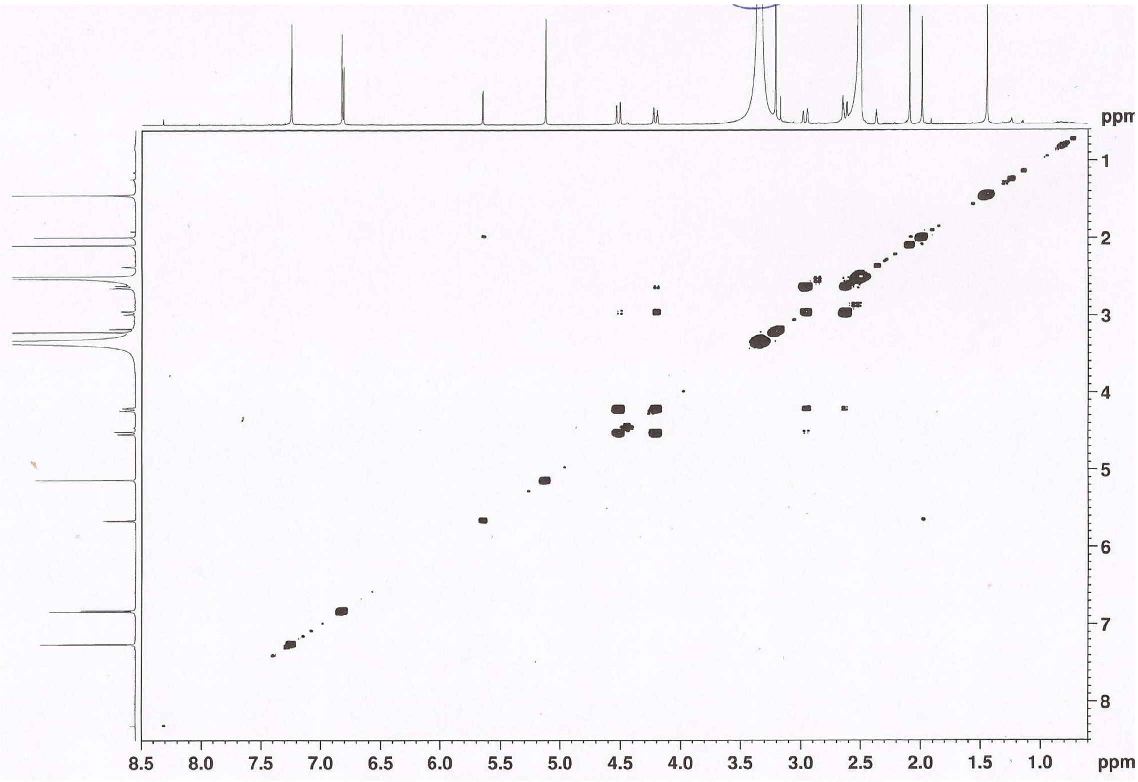

Figure S24. HSQC spectrum of a mixture of **2b** and **3b** (DMSO-d$_6$, 500.13 MHz).

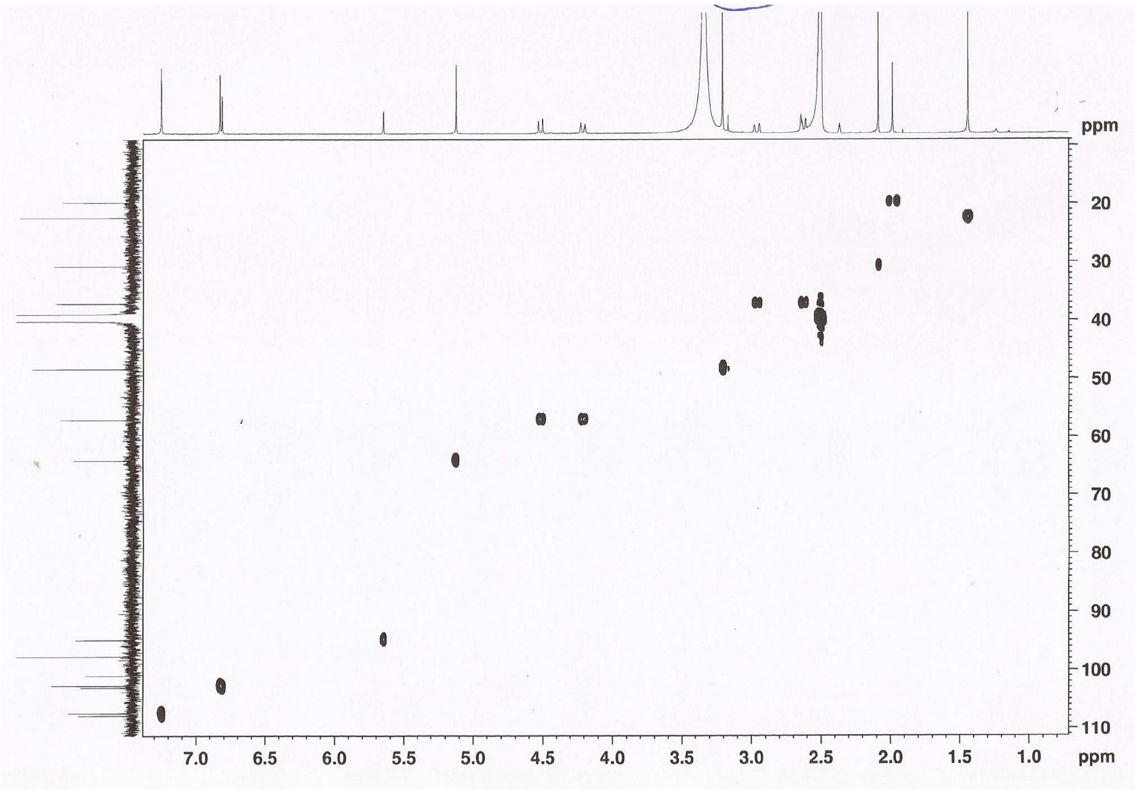

Figure S25. HMBC spectrum of a mixture of **2b** and **3b** (DMSO-d_6, 500.13 MHz).

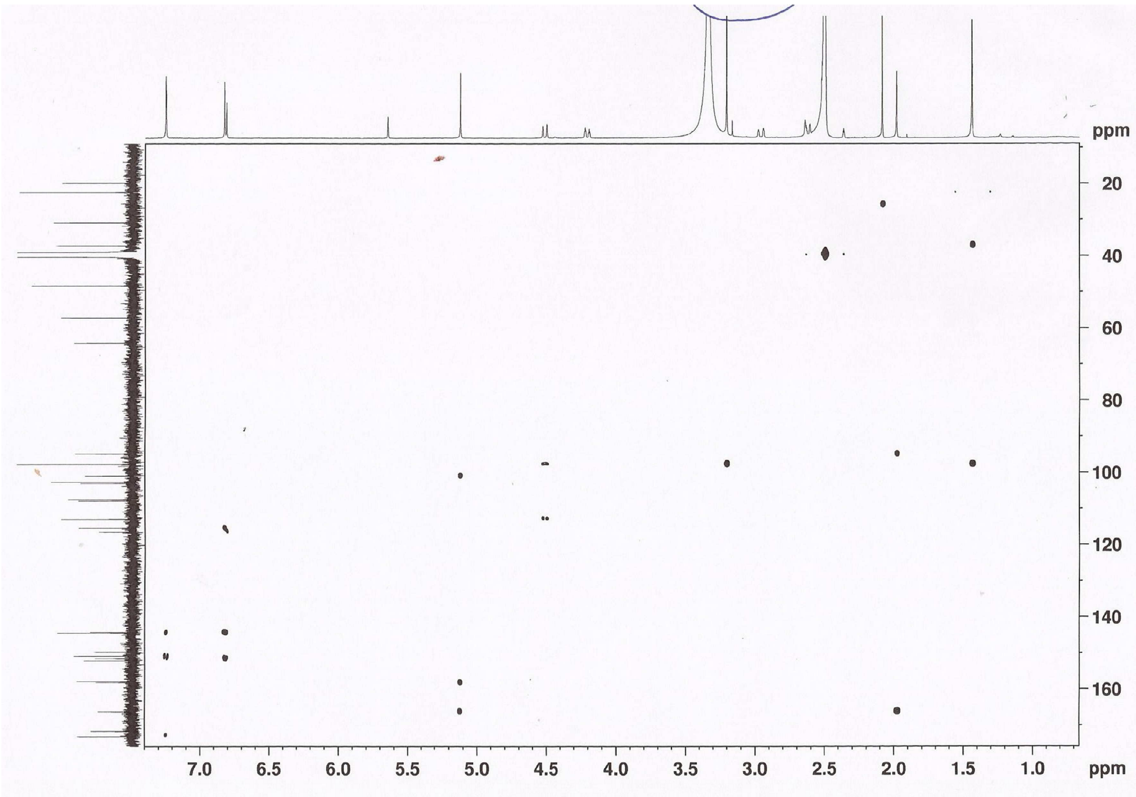

Figure S26. ^{1}H NMR spectrum of **2c** (DMSO-d_6, 500.13 MHz).

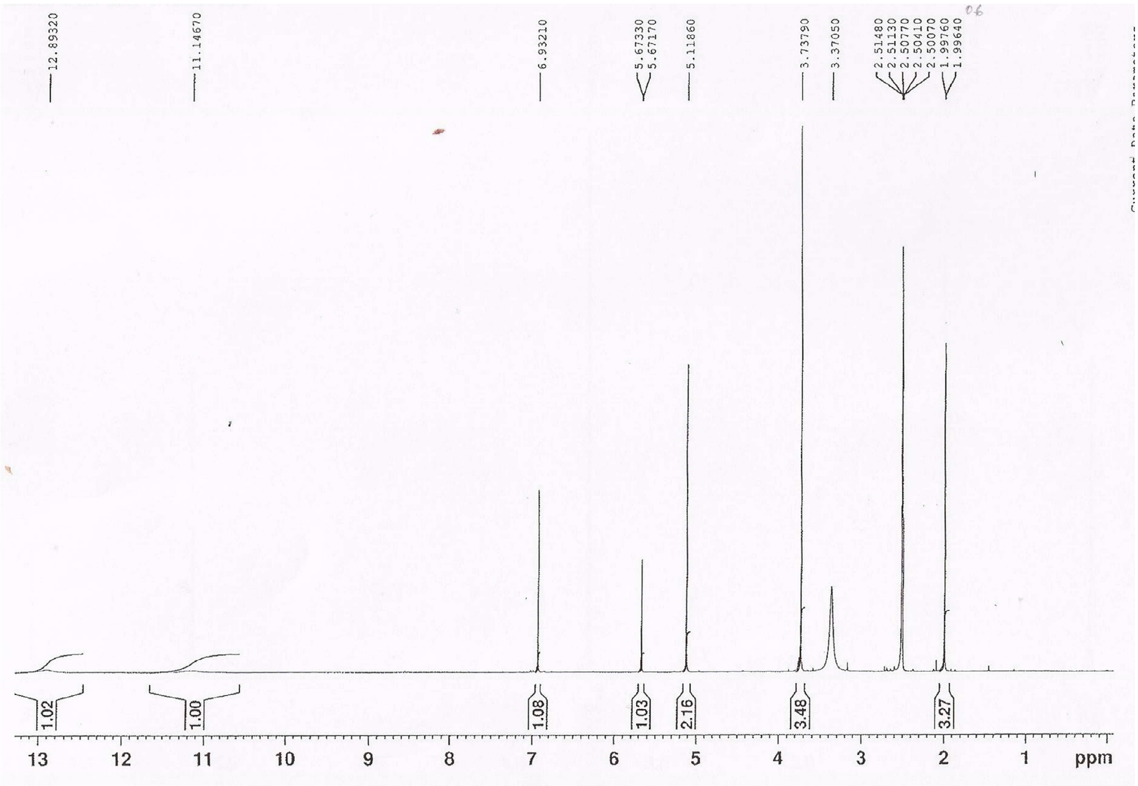

Figure S27. ^{13}C NMR spectrum of **2c** (DMSO-d$_6$, 125.4 MHz).

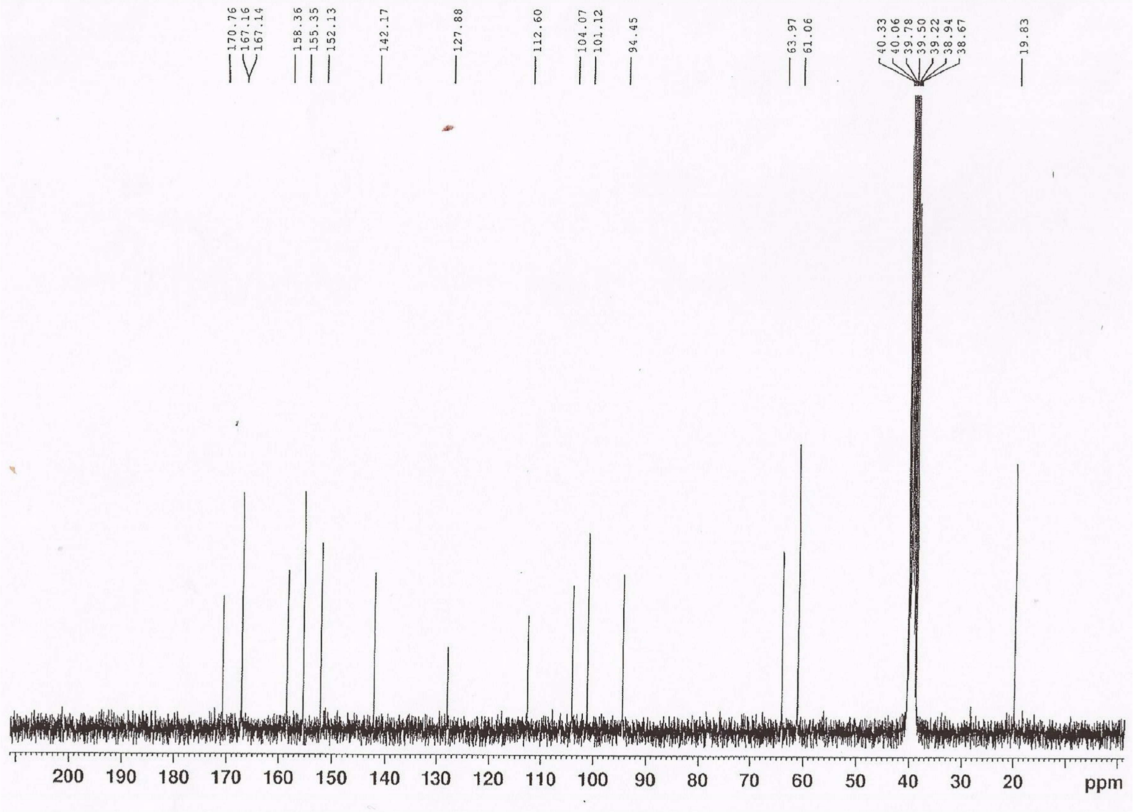

Figure S28. ^{1}H NMR spectrum of **3a** (DMSO-d$_6$, 300.13 MHz).

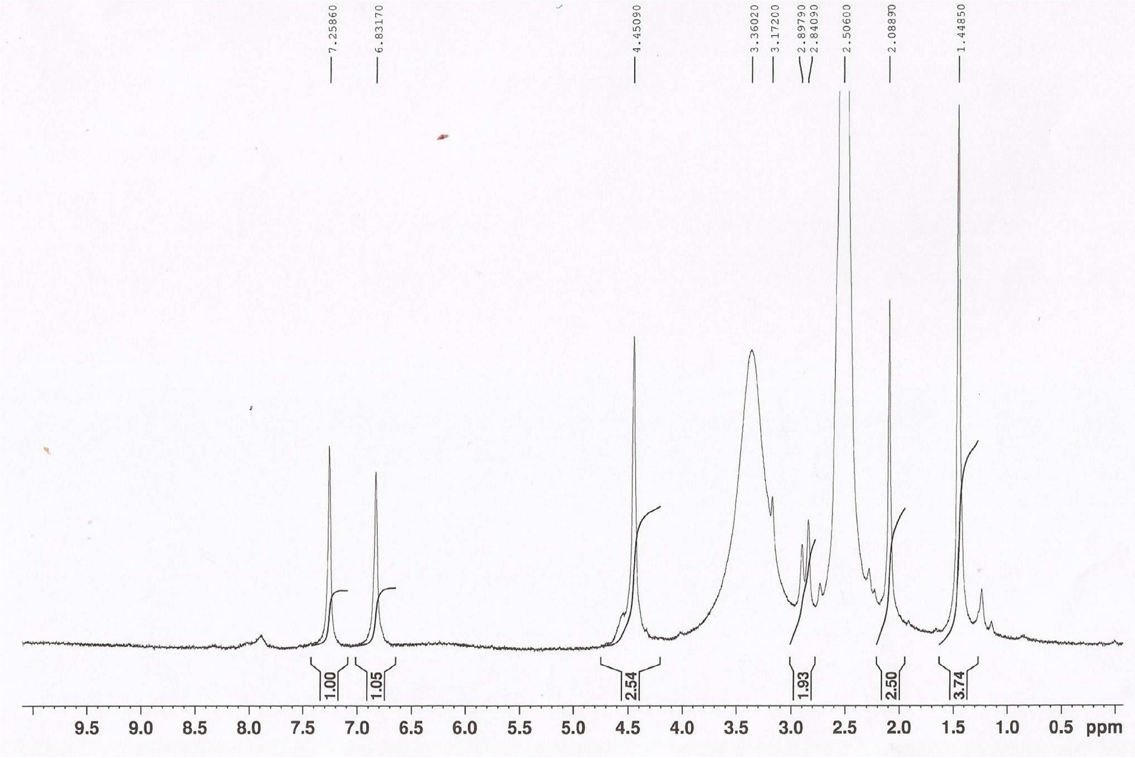

Figure S29. ^{13}C NMR spectrum of **3a** (DMSO-d$_6$, 75.4 MHz).

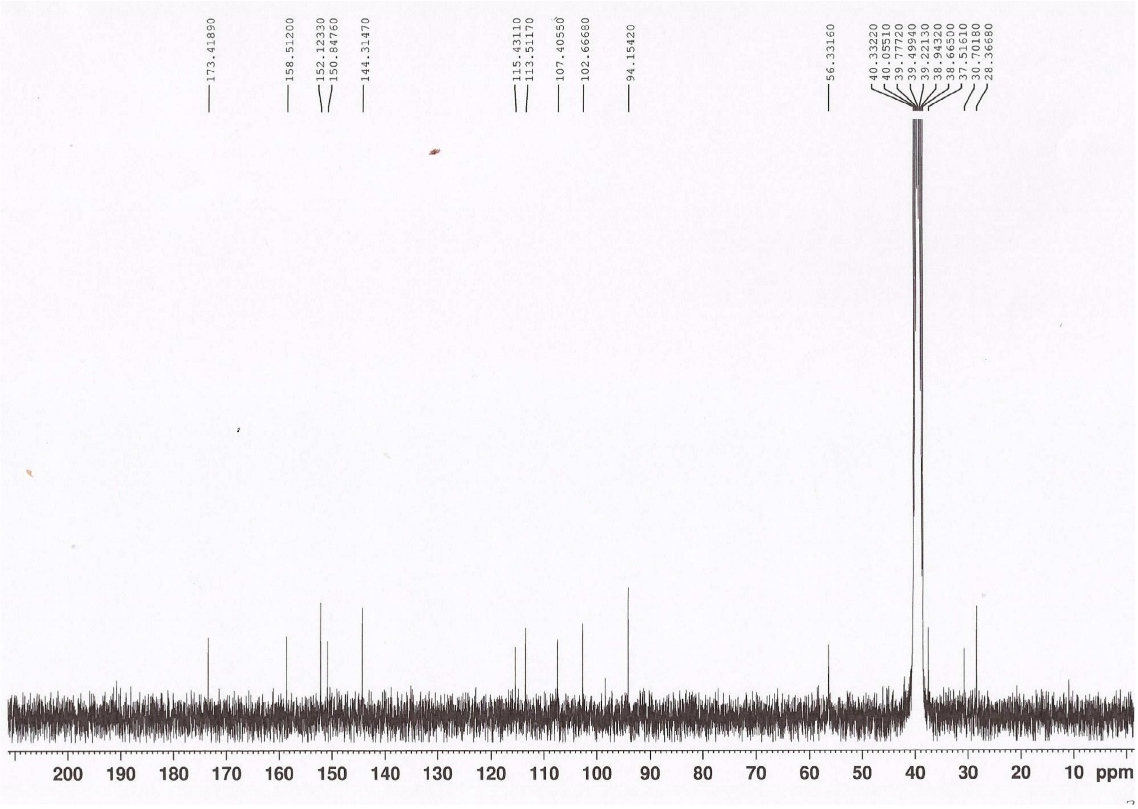

Figure S30. ^{1}H NMR spectrum of **4** (DMSO-d$_6$, 300.13 MHz).

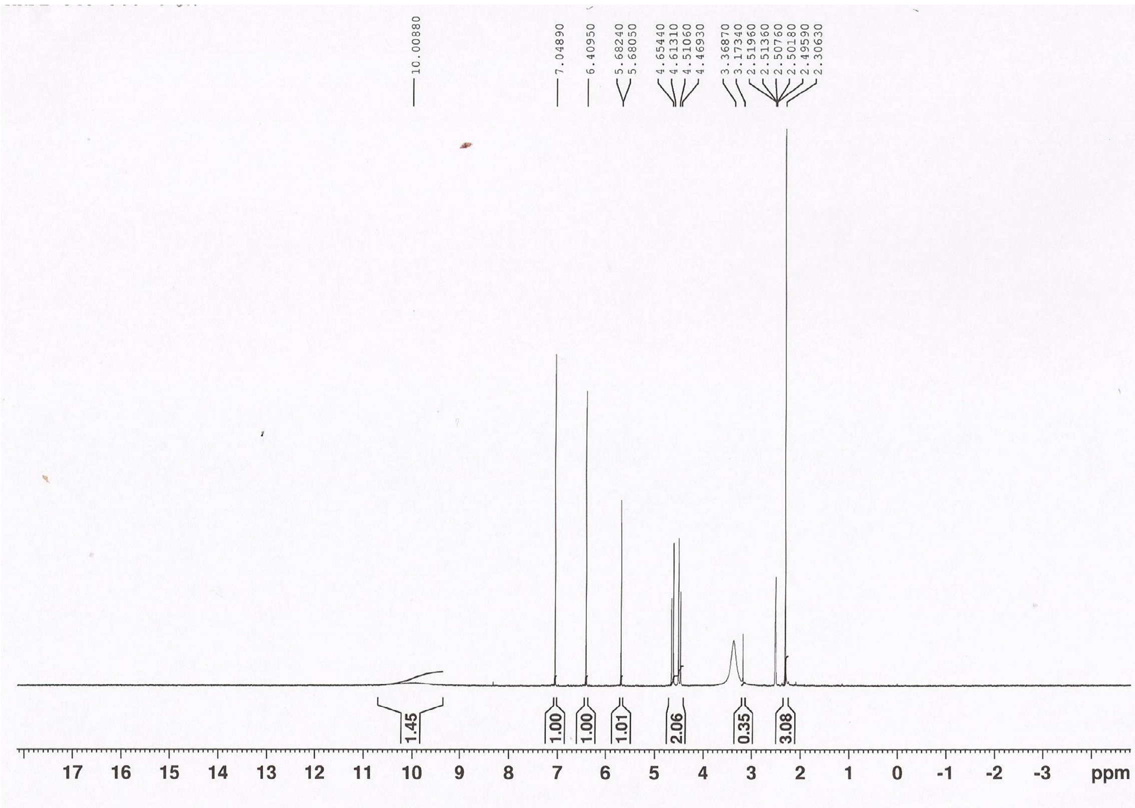

Figure S31. ^{13}C NMR spectrum of **4** (DMSO-d$_6$, 75.4 MHz).

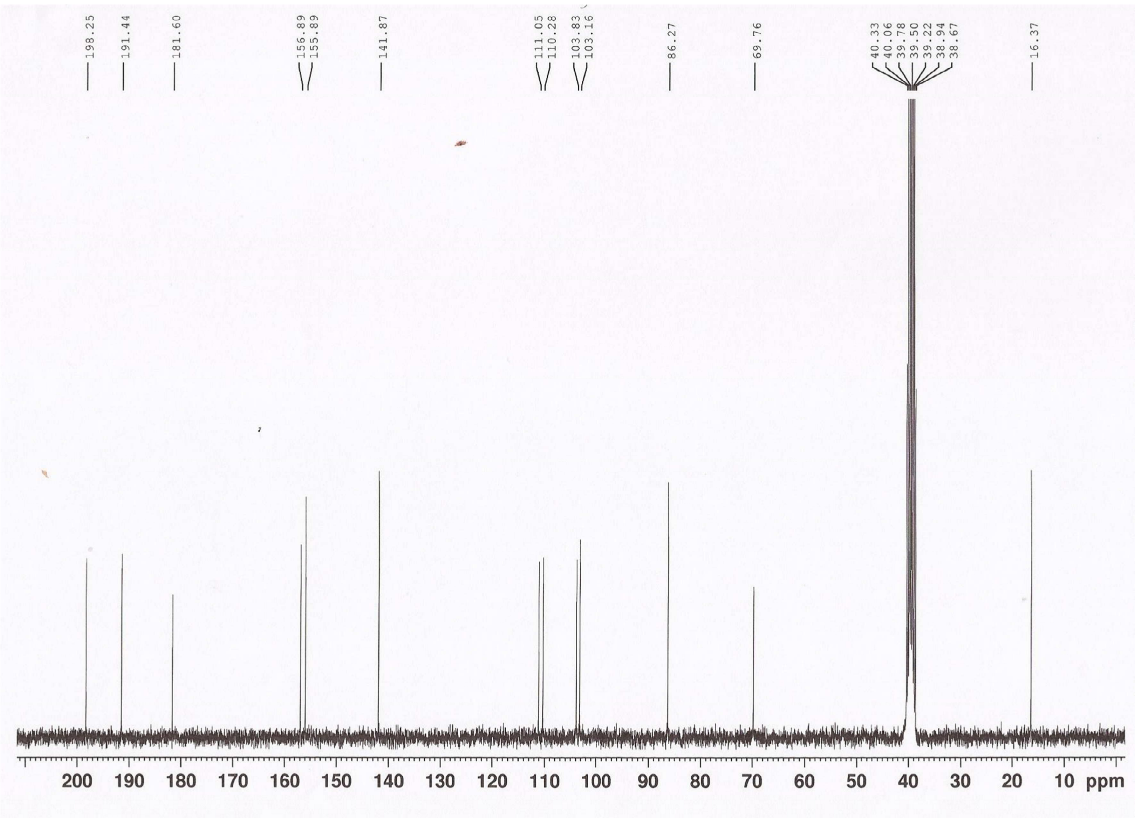

Figure S32. COSY spectrum of **4** (DMSO-d$_6$, 300.13 MHz).

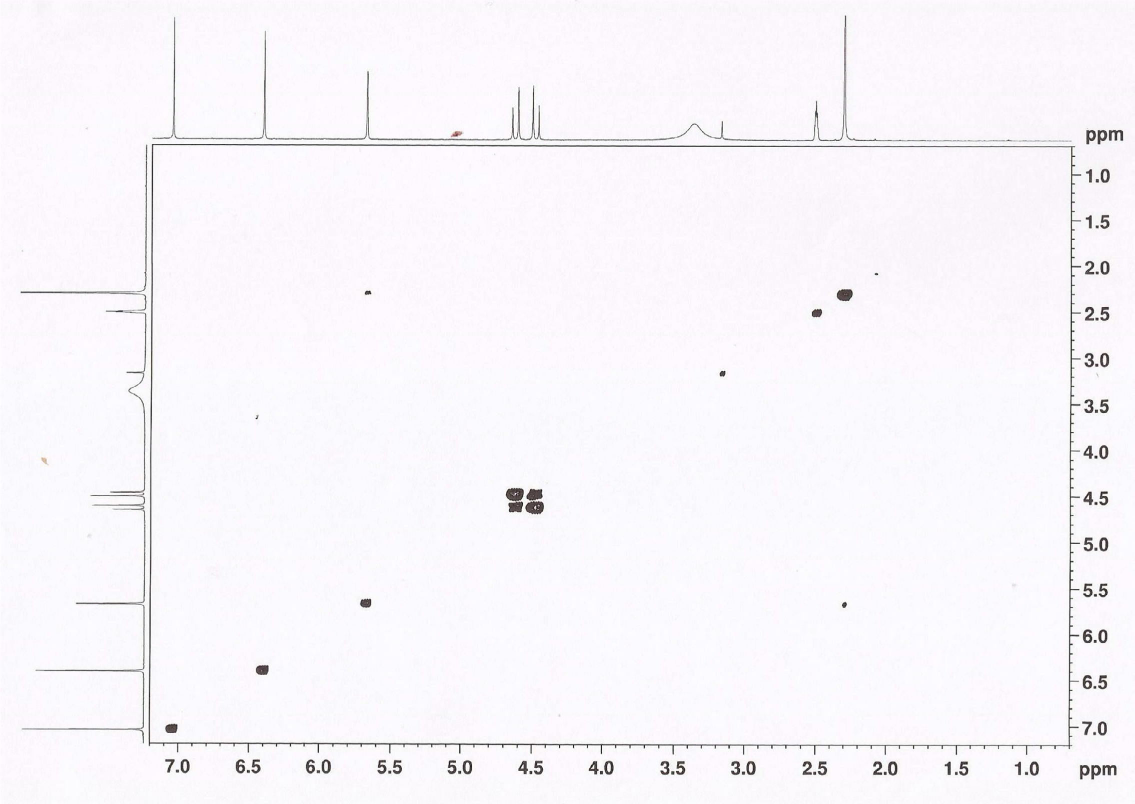

Figure S33. HSQC spectrum of **4** (DMSO-d$_6$, 300.13 MHz).

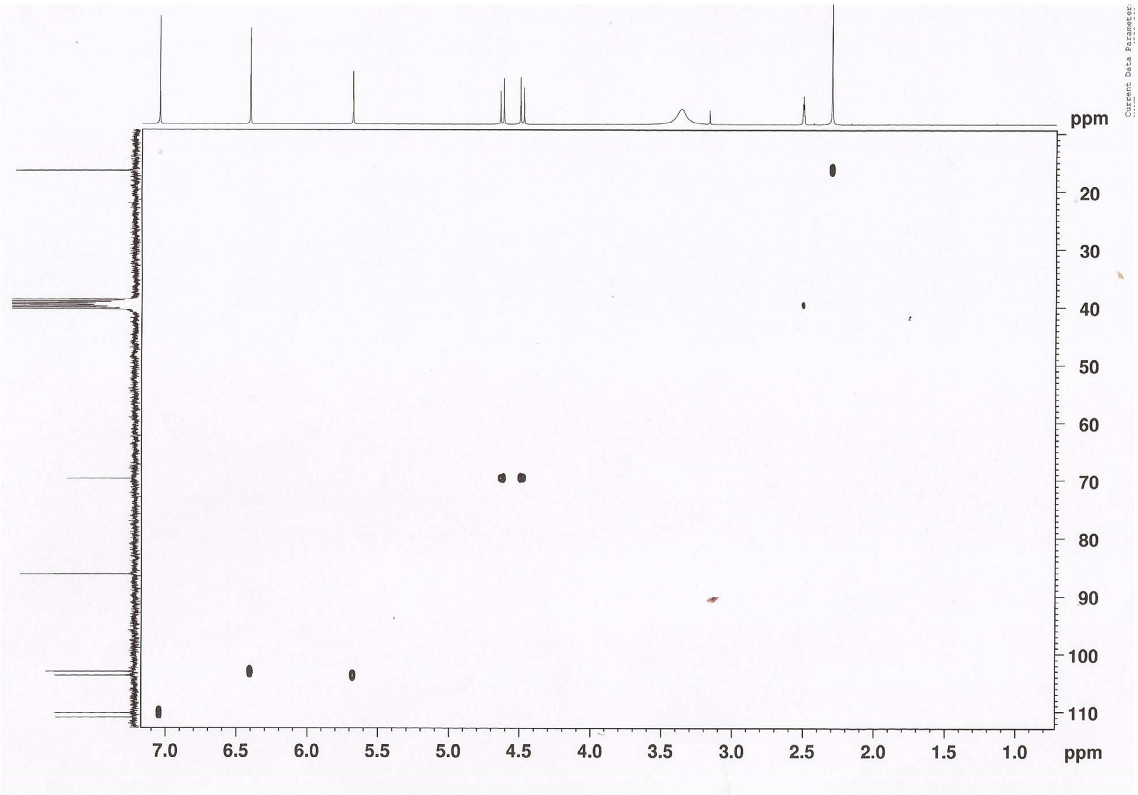

Figure S34. HMBC spectrum of **4** (DMSO-d$_6$, 300.13 MHz).

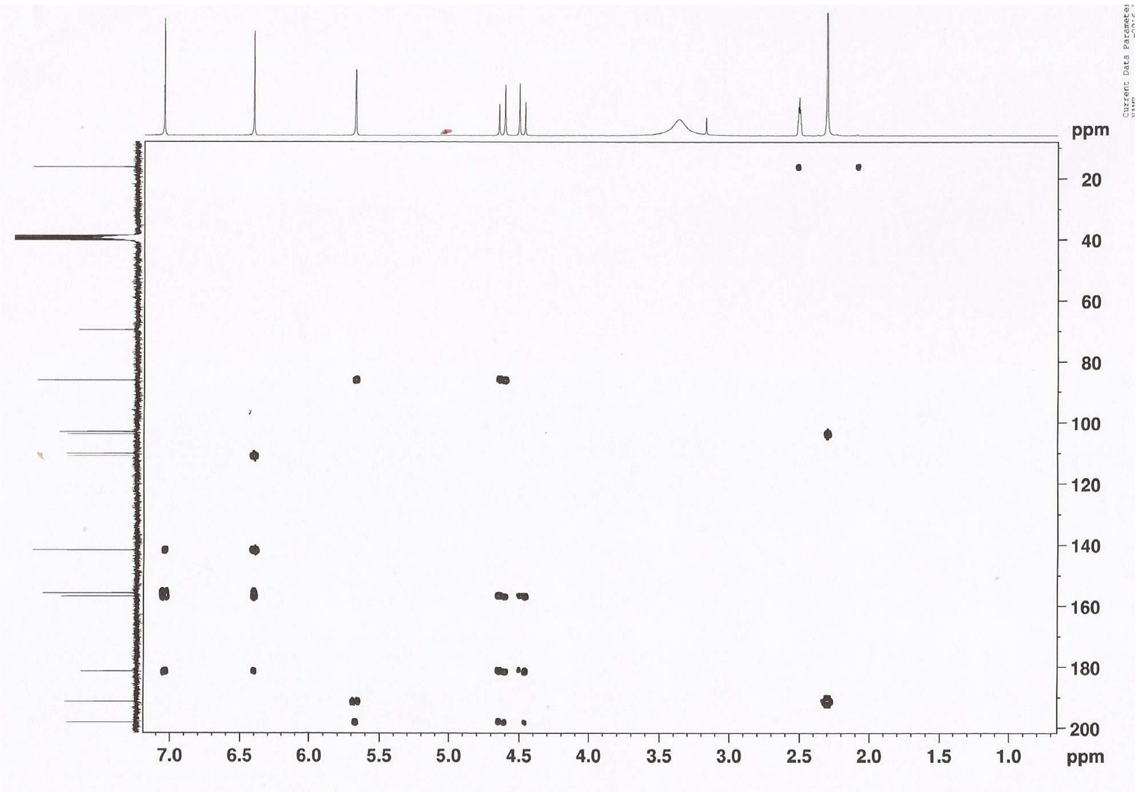

Figure S35. ^{1}H NMR spectrum of **5** (DMSO-d_6, 500.13 MHz).

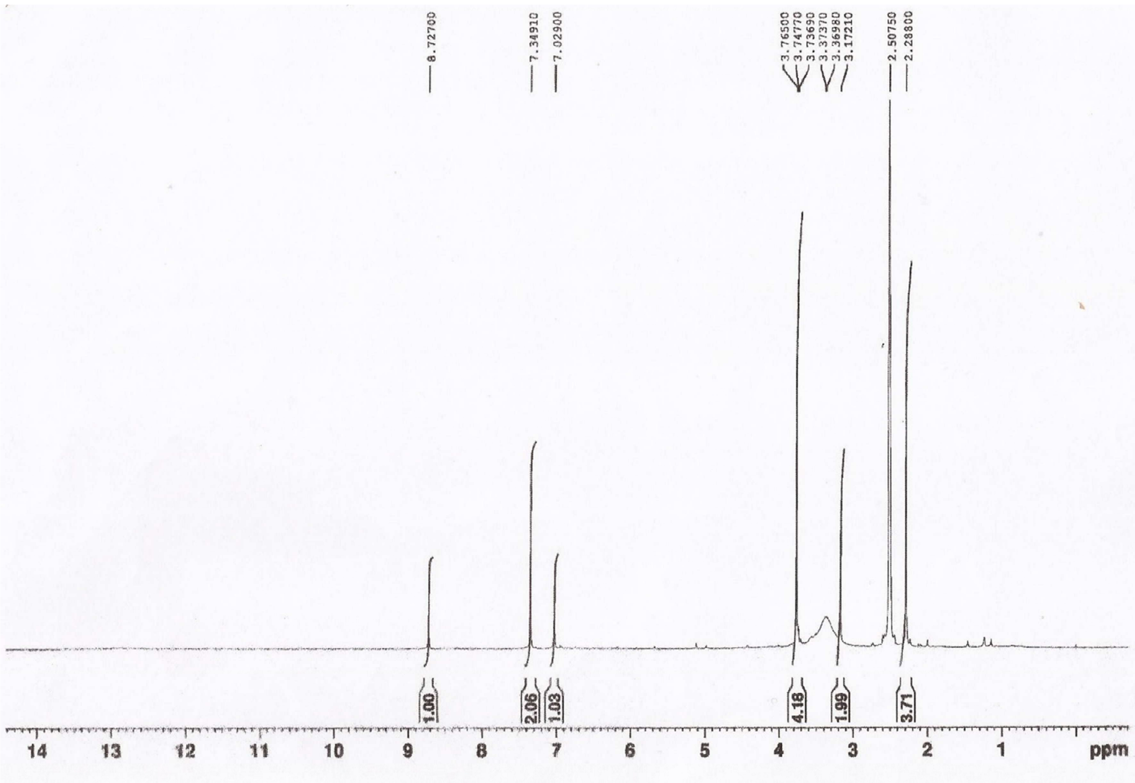

Figure S36. ^{13}C NMR spectrum of **5** (DMSO-d_6, 125.4 MHz).

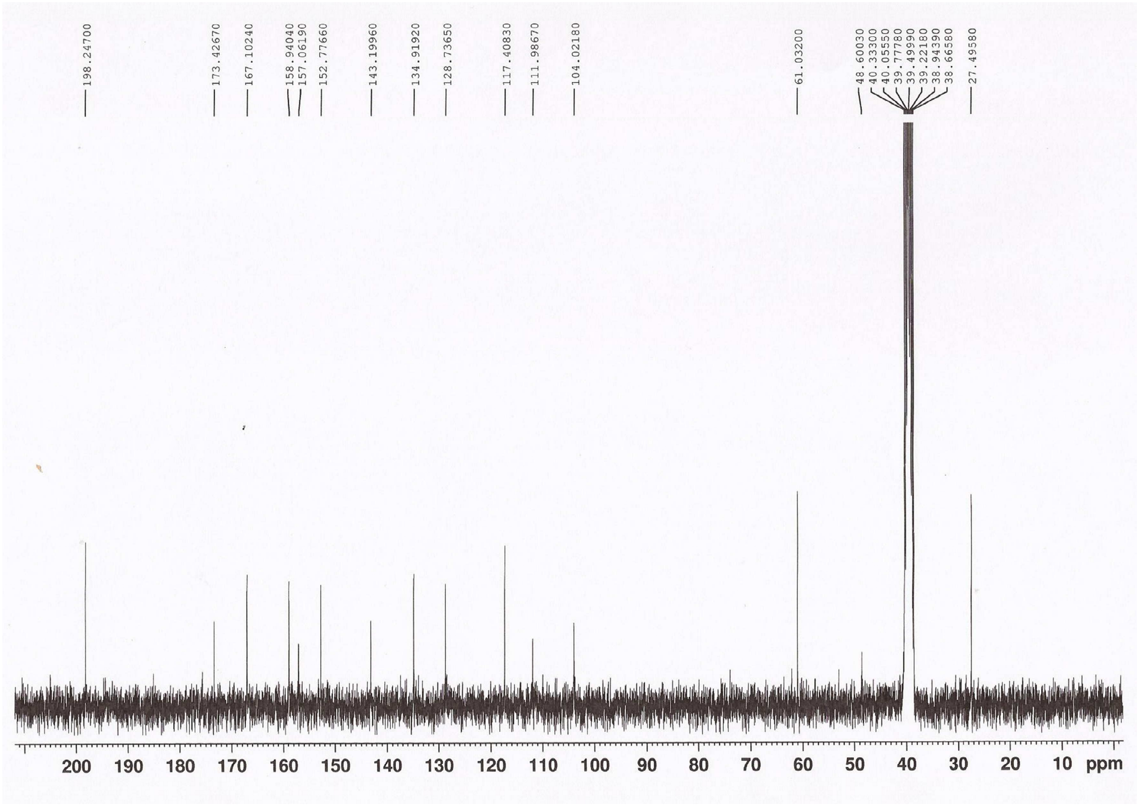

Figure S37. HSQC spectrum of **5** (DMSO-d$_6$, 500.13 MHz).

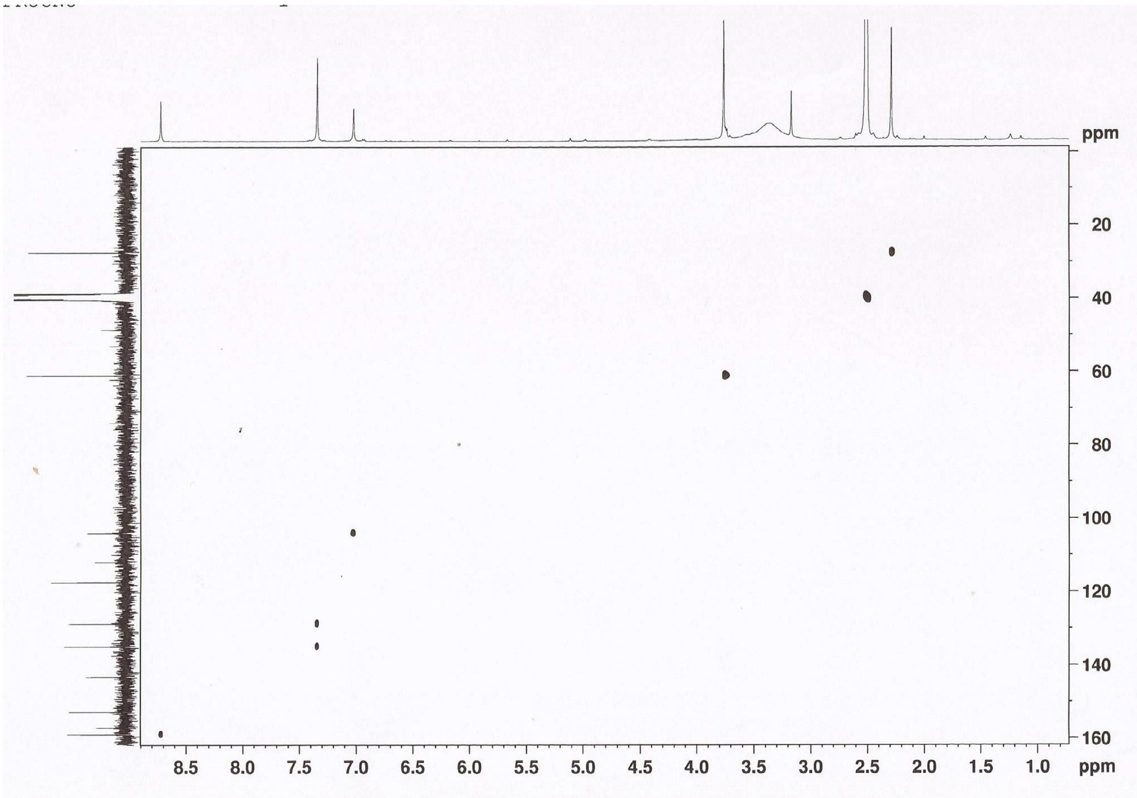

Figure S38. HMBC spectrum of **5** (DMSO-d$_6$, 500.13 MHz).

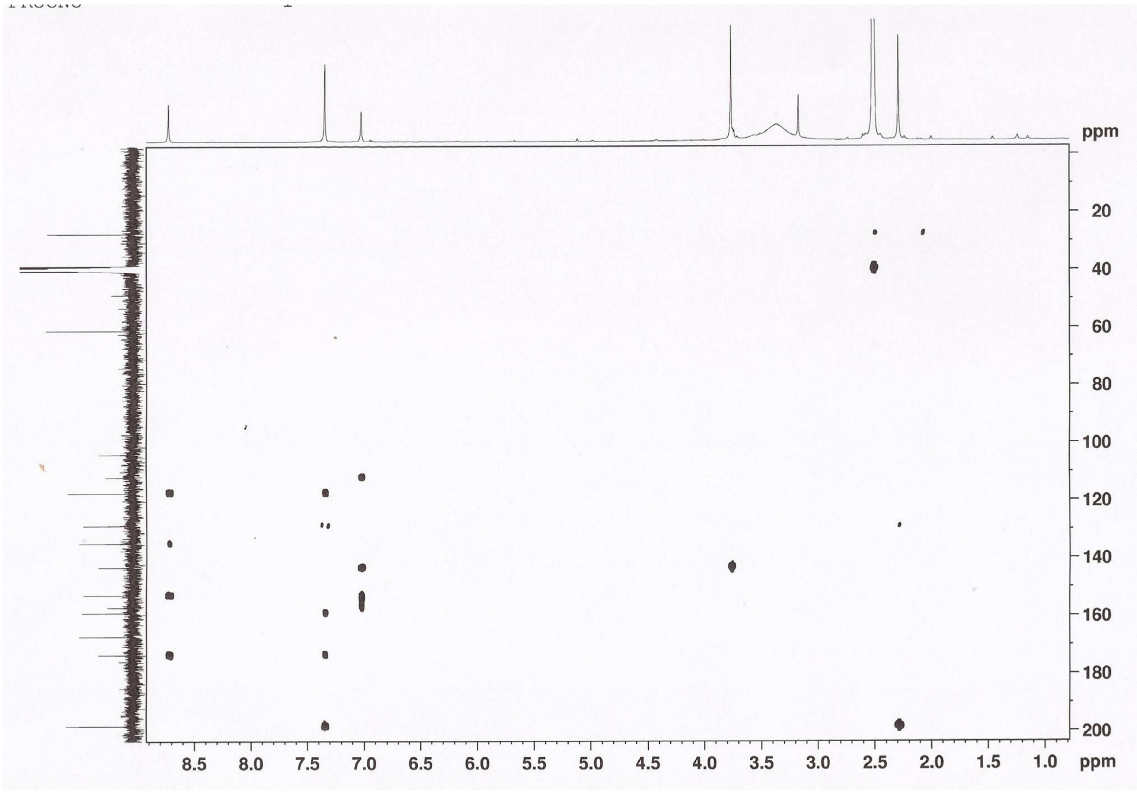

Figure S39. ^{1}H NMR spectrum of **6** (DMSO-d_6, 500.13 MHz).

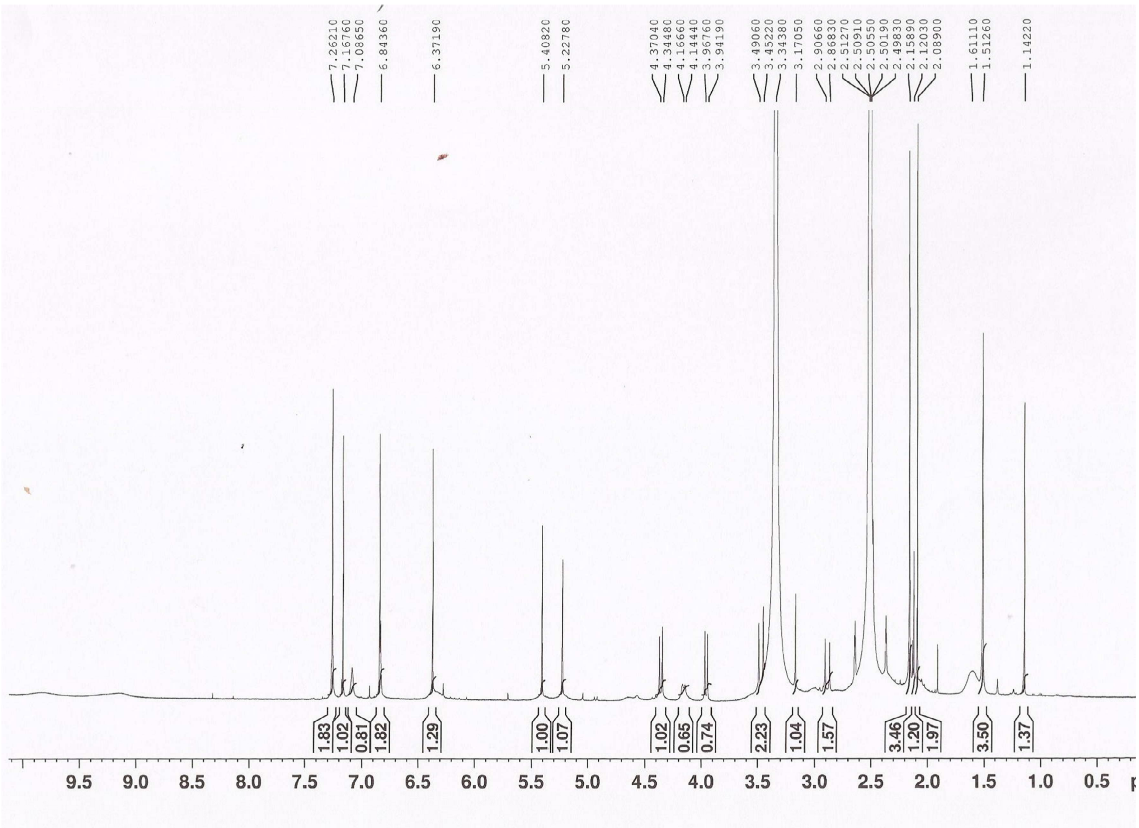

Figure S40. ^{13}C NMR spectrum of **6** (DMSO-d_6, 125.4 MHz).

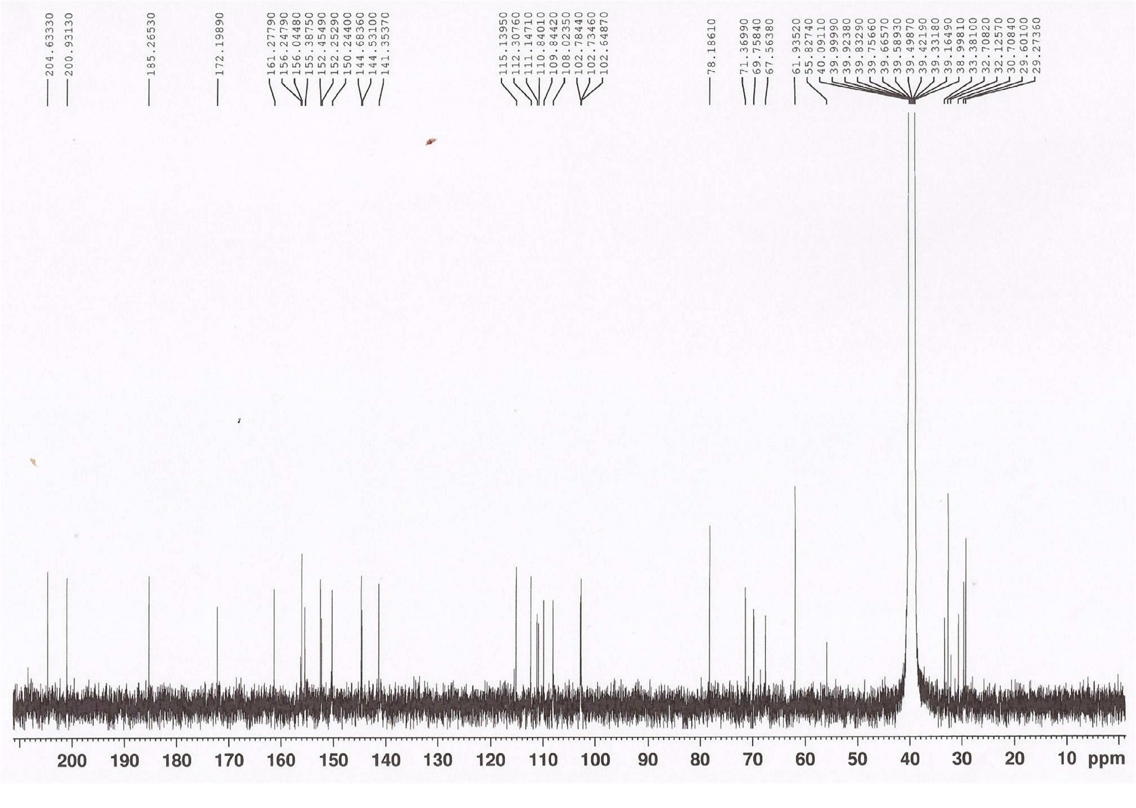

Figure S41. COSY spectrum of **6** (DMSO-d$_6$, 500.13 MHz).

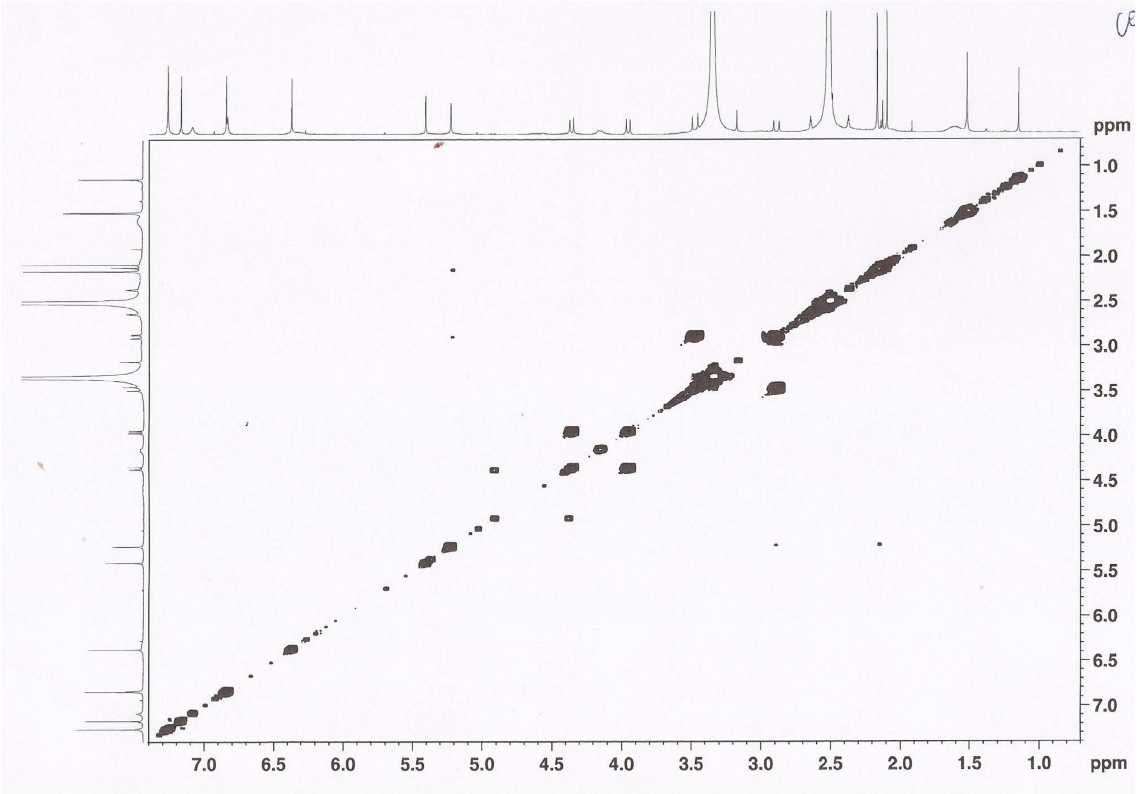

Figure S42. HSQC spectrum of **6** (DMSO-d$_6$, 500.13 MHz).

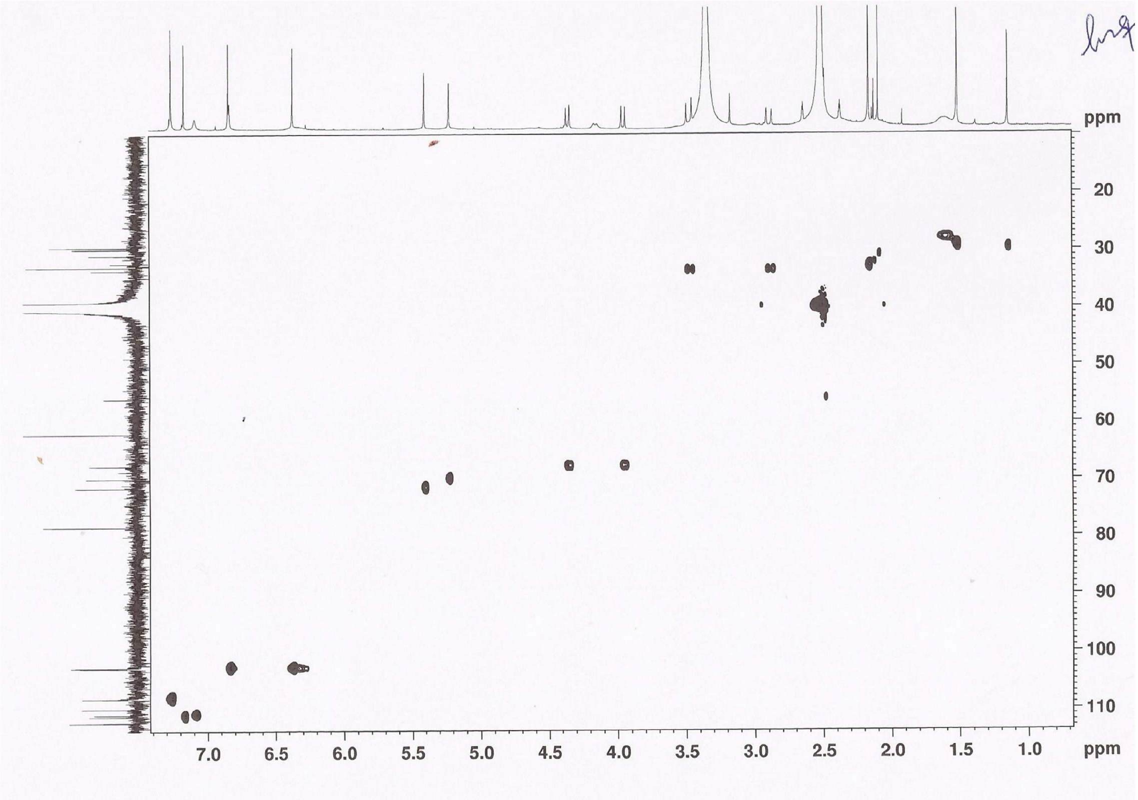

Figure S43. HMBC spectrum of **6** (DMSO-d$_6$, 500.13 MHz).

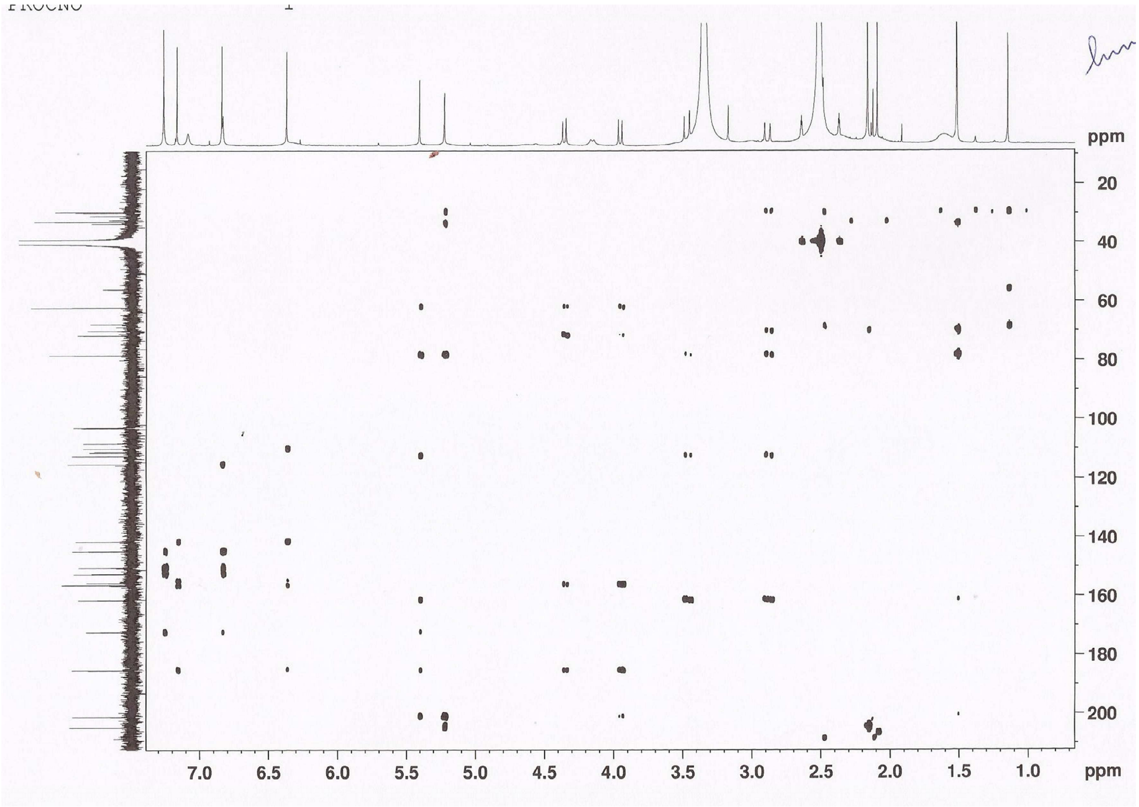

Figure S44. ROESY spectrum of **6** (DMSO-d$_6$, 500.13 MHz).

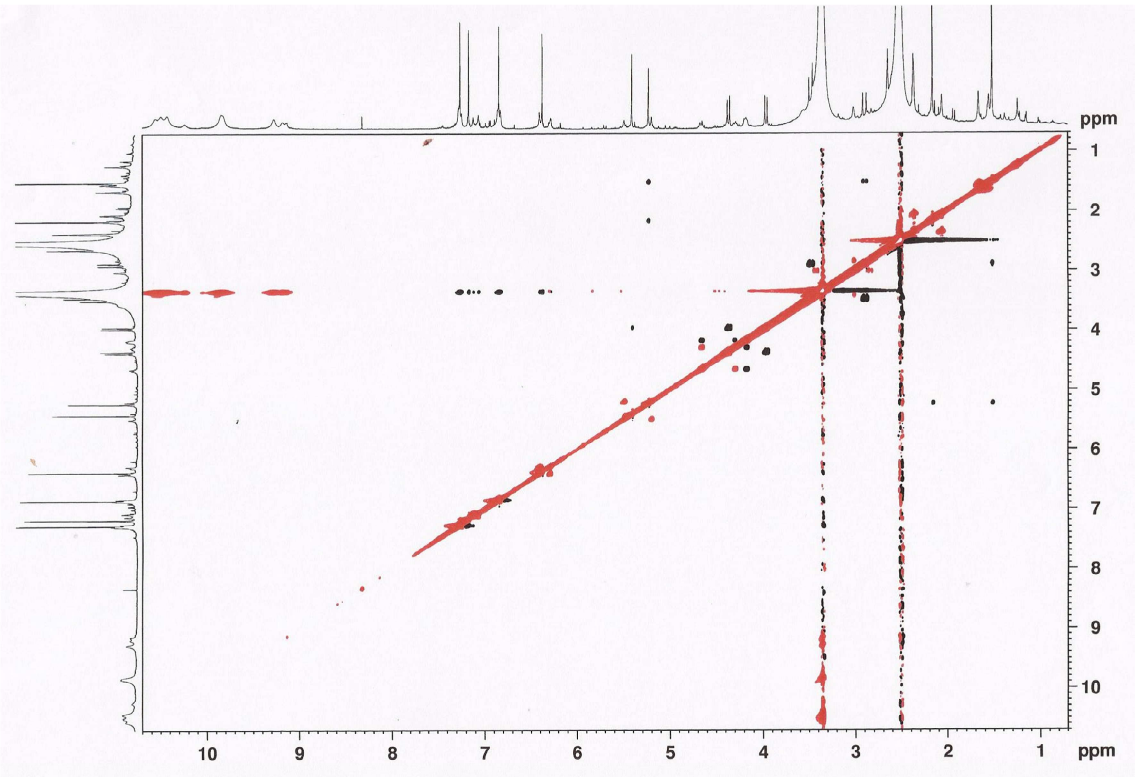

Figure S45. ^{1}H NMR spectrum of **7** (DMSO-d_6, 500.13 MHz).

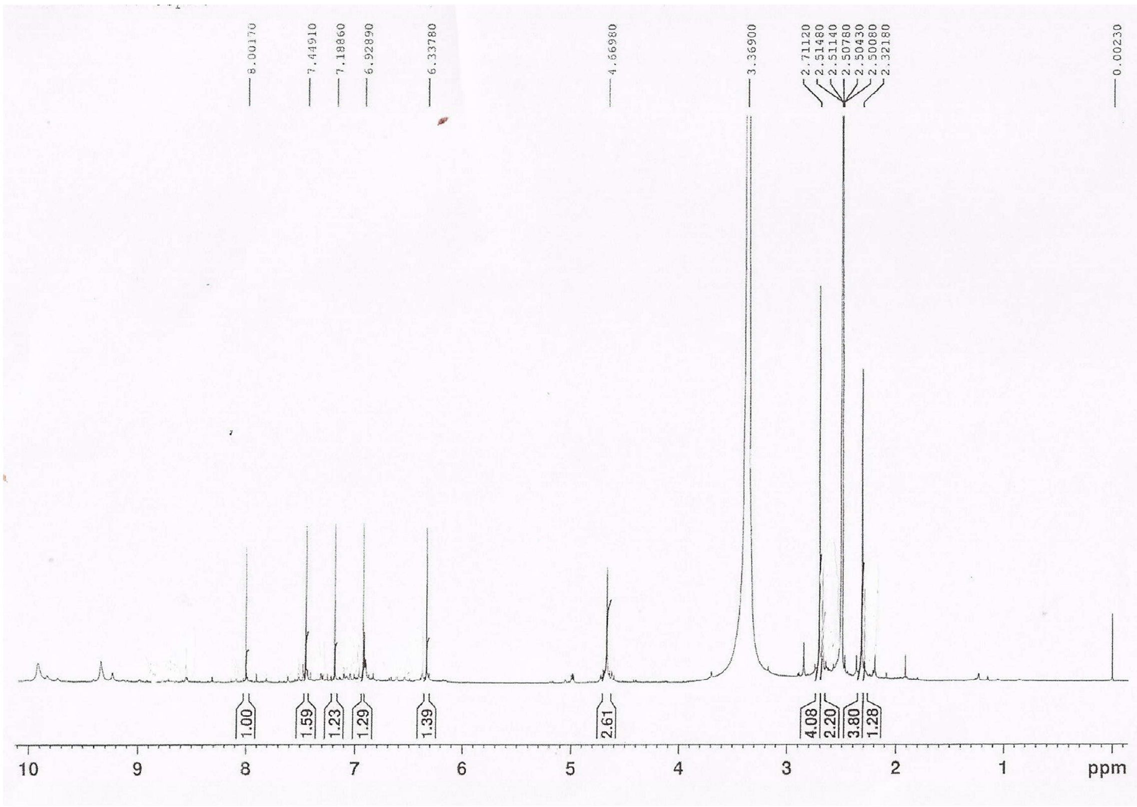

Figure S46. ^{13}C NMR spectrum of **7** (DMSO-d_6, 125.4 MHz).

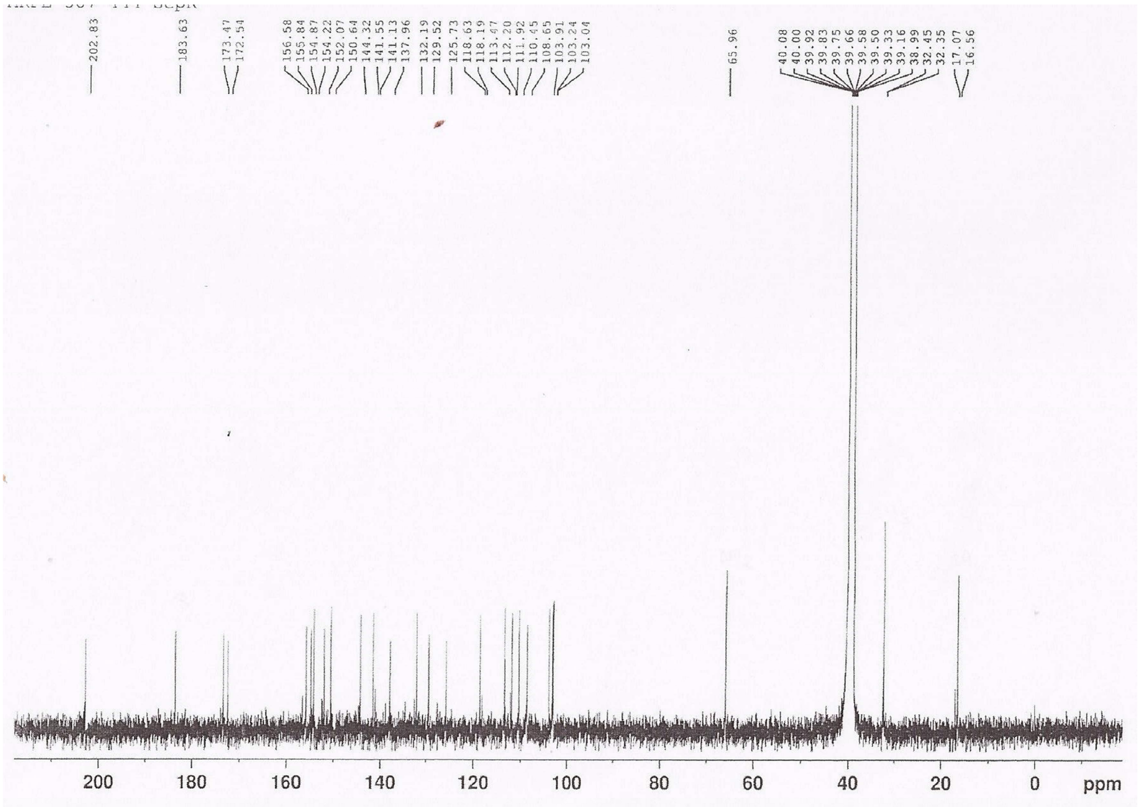

Figure S47. HSQC spectrum of **7** (DMSO-d$_6$, 500.13 MHz).

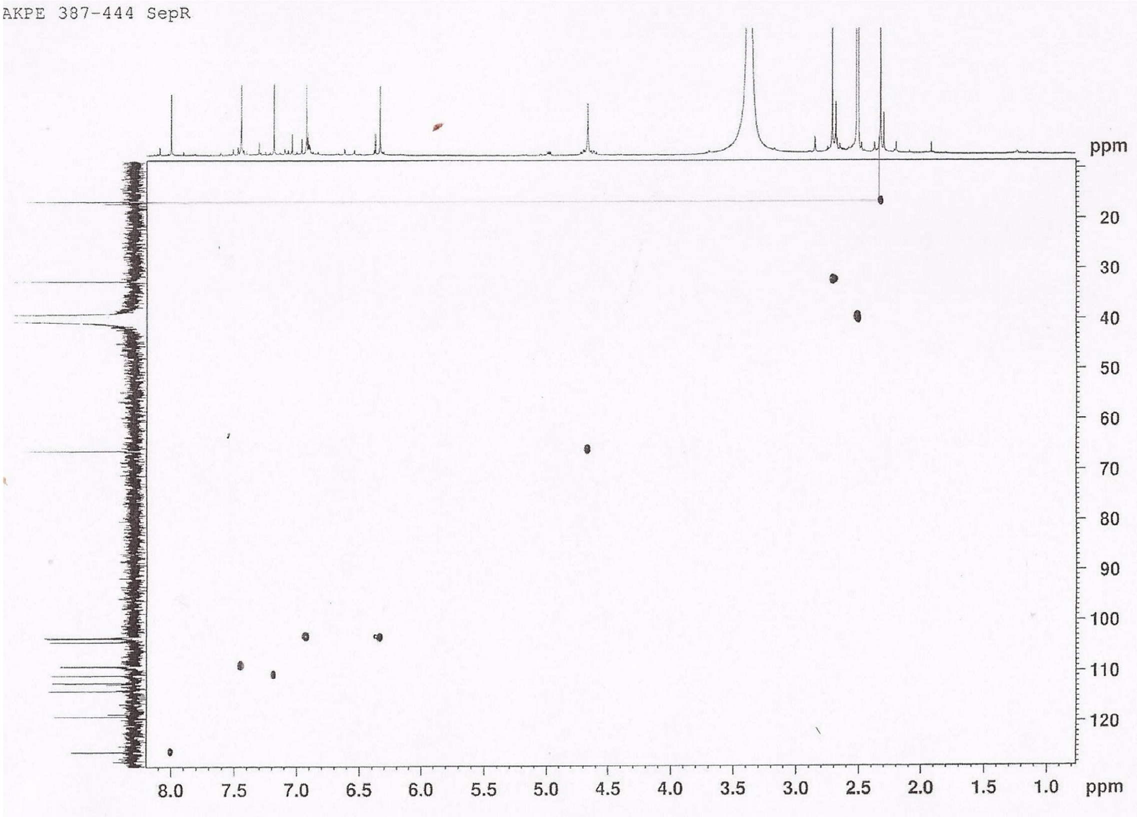

Figure S48. HMBC spectrum of **7** (DMSO-d$_6$, 500.13 MHz).

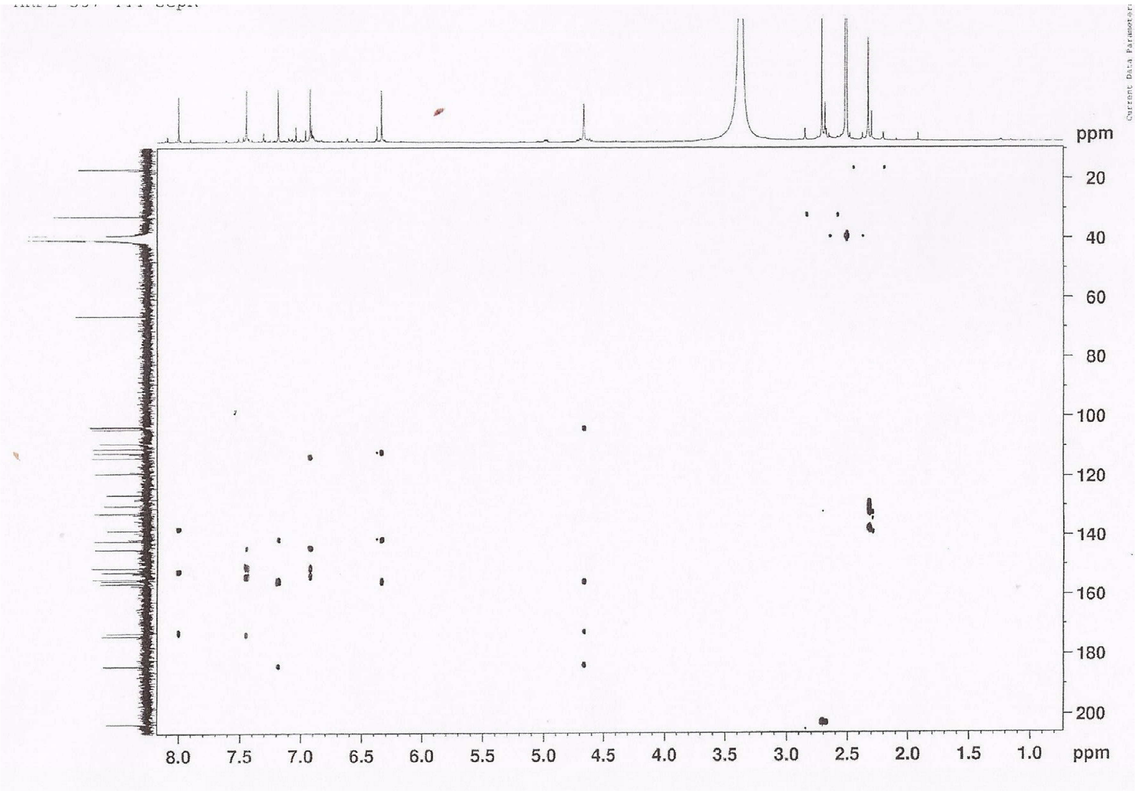

Figure S49. ^{1}H NMR spectrum of **8** (DMSO-d$_6$, 300.13 MHz).

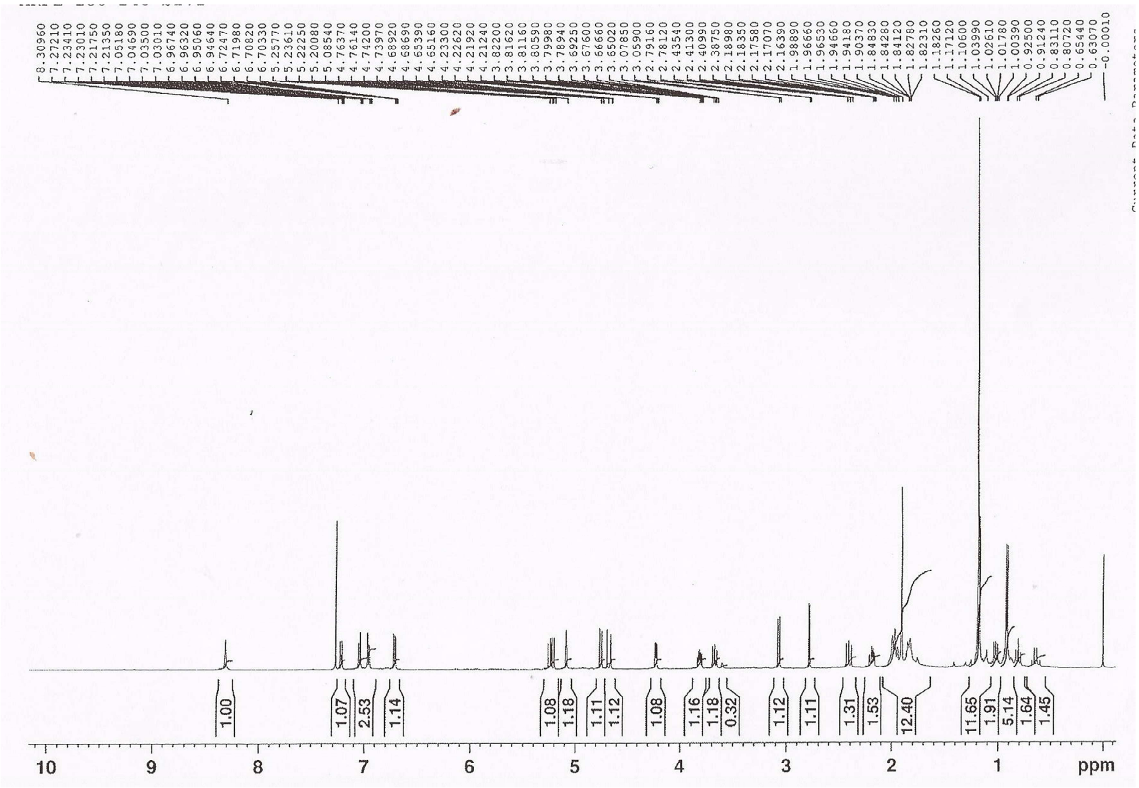

Figure S50. ^{13}C NMR spectrum of **8** (DMSO-d$_6$, 75.4 MHz).

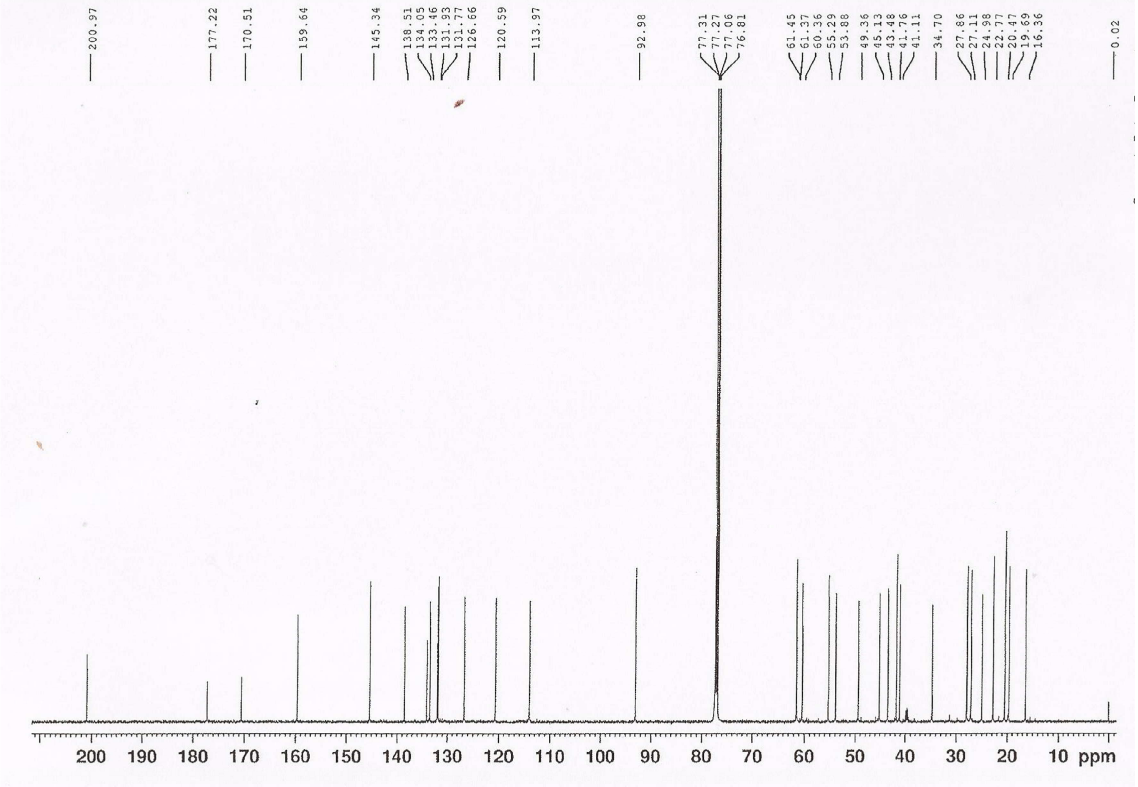

Figure S51. ORTEP view of **8**.

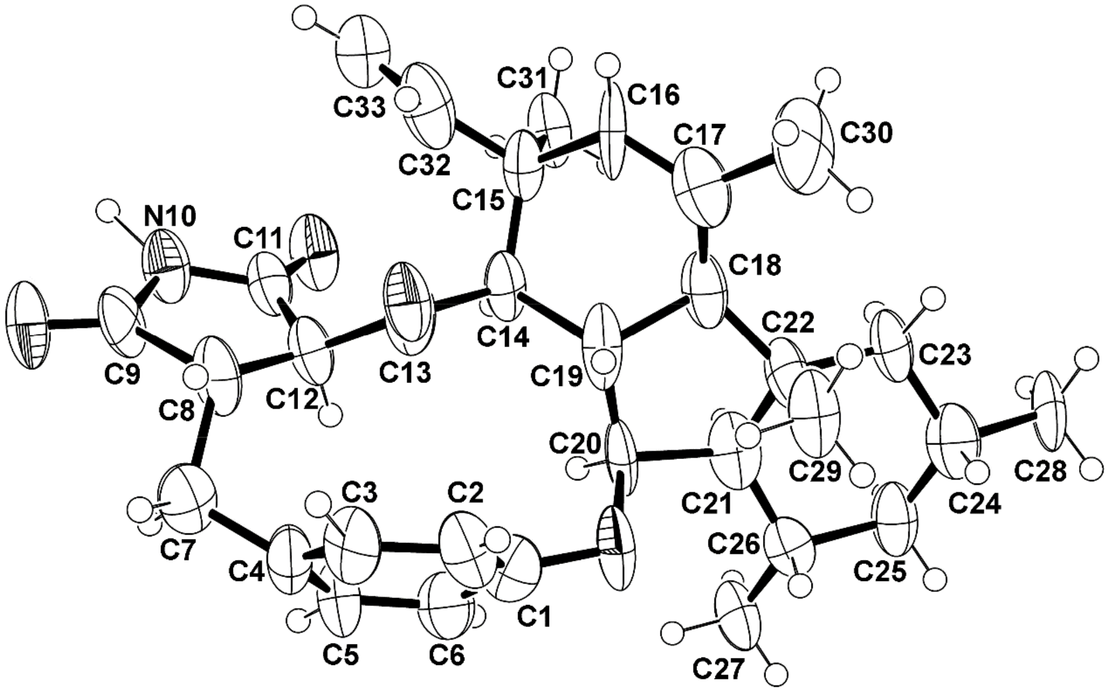

Figure S52. ^{1}HNMR spectrum of **9** (DMSO-d$_6$, 500.3 MHz).

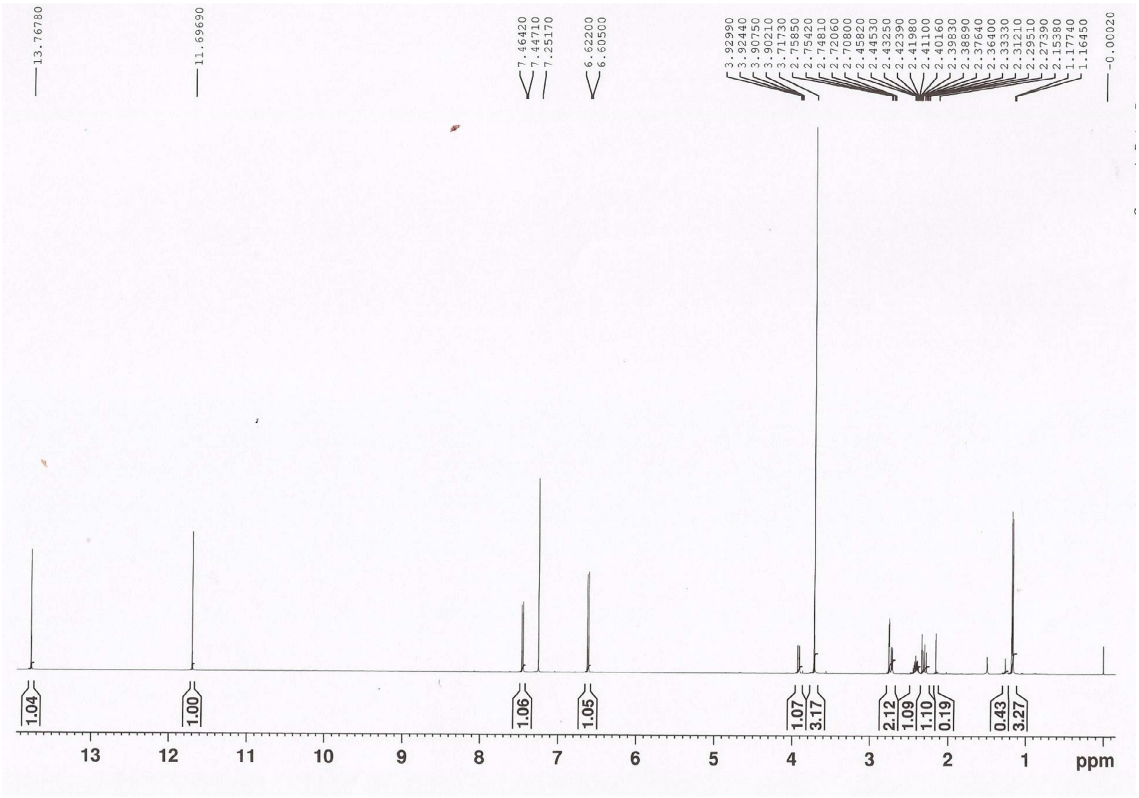

Figure S53. ^{13}CNMR spectrum of **9** (DMSO-d$_6$, 125.4 MHz).

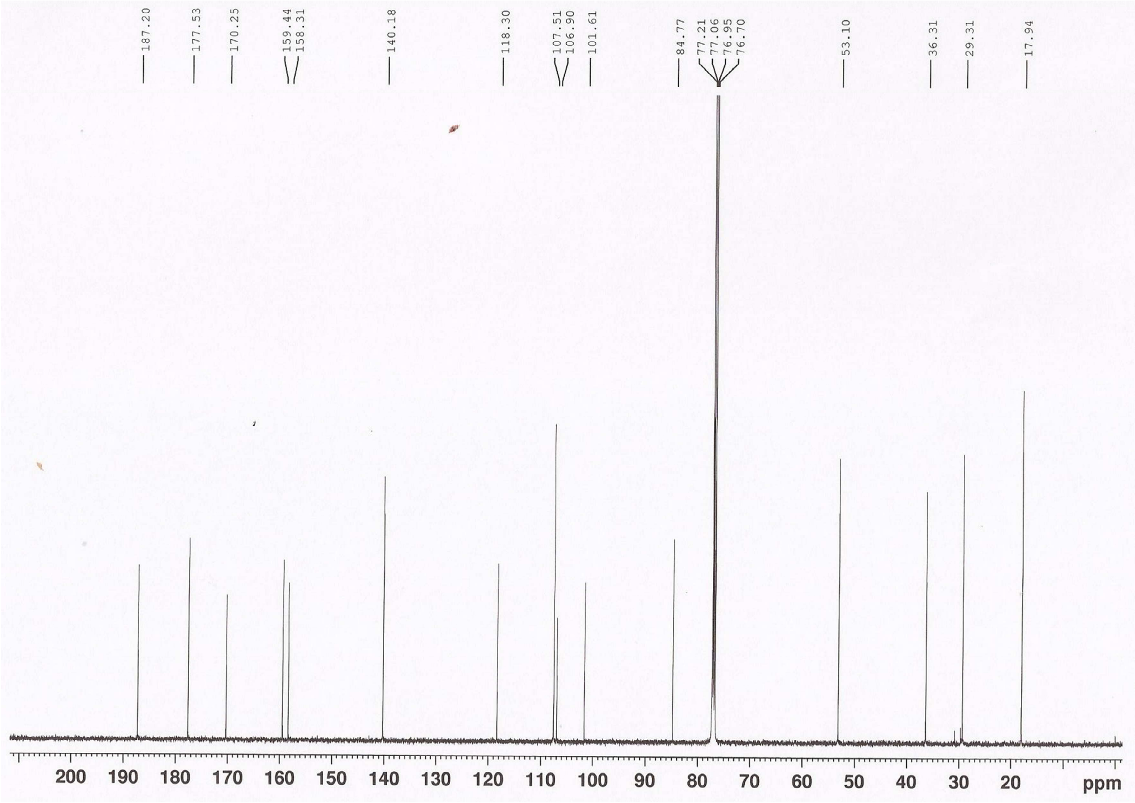

Supplement: Supplementary file 1 [file marinedrugs-16-00289-s001.pdf]
